# Fifty years after: A taxonomic revision of the amphibian species from the Ecuadorian biodiversity hotspot Abra de Zamora, with description of two new *Pristimantis species*

**DOI:** 10.1371/journal.pone.0238306

**Published:** 2020-09-10

**Authors:** Paul Székely, Juan Sebastián Eguiguren, Leonardo Ordóñez-Delgado, Diego Armijos-Ojeda, Diana Székely

**Affiliations:** 1 Museo de Zoología, Universidad Técnica Particular de Loja, Loja, Ecuador; 2 Laboratorio de Ecología Tropical y Servicios Ecosistémicos - EcoSs Lab, Departamento de Ciencias Biológicas, Universidad Técnica Particular de Loja, Loja, Ecuador; 3 Biotechnology Center (BIOTEC), Center for Molecular and Cellular Bioengineering (CMCB), Technische Universität Dresden (TUD), Dresden, Germany; 4 Programa de Doctorado en Conservación de Recursos Naturales, Escuela Internacional de Doctorado, Universidad Rey Juan Carlos, Móstoles, Madrid, Spain; 5 Faculty of Natural and Agricultural Sciences, Ovidius University Constanța, Constanța, Romania; Universitat Trier, GERMANY

## Abstract

Abra de Zamora is an important biodiversity hotspot in southern Ecuador. Between 1938 and 2010, eleven species of frogs were described from here: *Lynchius flavomaculatus*, *Gastrotheca psychrophila*, *Pristimantis balionotus*, *P*. *colodactylus*, *P*. *cryptomelas*, *P*. *percultus*, *P*. *versicolor*, *P*. *vidua*, *Telmatobius cirrhacelis*, *P*. *andinognomus*, and *Atelopus podocarpus*. Unfortunately, many of these species were not re-encountered after their original description, and for the majority DNA samples were not available, making their phylogenetic position unknown. In this study, we assess the current state of the amphibians from Abra de Zamora, by: i. redescribing the species which were first reported from the area, by contributing genetic delimitation (for *L*. *flavomaculatus*, *P*. *balionotus*, *P*. *colodactylus*, *P*. *percultus*, and *P*. *vidua*), release call (*L*. *flavomaculatus*) and advertisement call descriptions (for *P*. *balionotus*, *P*. *vidua* and *P*. *versicolor*); ii. presenting an updated amphibian species list of Abra de Zamora, with the description of two additional *Pristimantis* species; iii. updating the distribution of these species, including data collected in similar montane habitats from surrounding areas; and iv. amending recommendations regarding their conservation status.

## Introduction

Despite its small size, Ecuador is one of the most biodiverse countries [1], showing the highest density of species (number of species per area unit) in the case of amphibians [2] and reptiles [3], as well as a remarkable proportion of endemic species [4]. Several areas in Ecuador are recognized as genuine hotspots of amphibian diversity from where numerous species were described, such as Parque Nacional Yasuní in western Amazon [5] or Parque Nacional Sangay [6] and the upper basin of the Pastaza River [7] in Tropical Andes. One such a biodiverse hotspot, with a comparatively much smaller extension, is Abra de Zamora.

**Fig 1:**
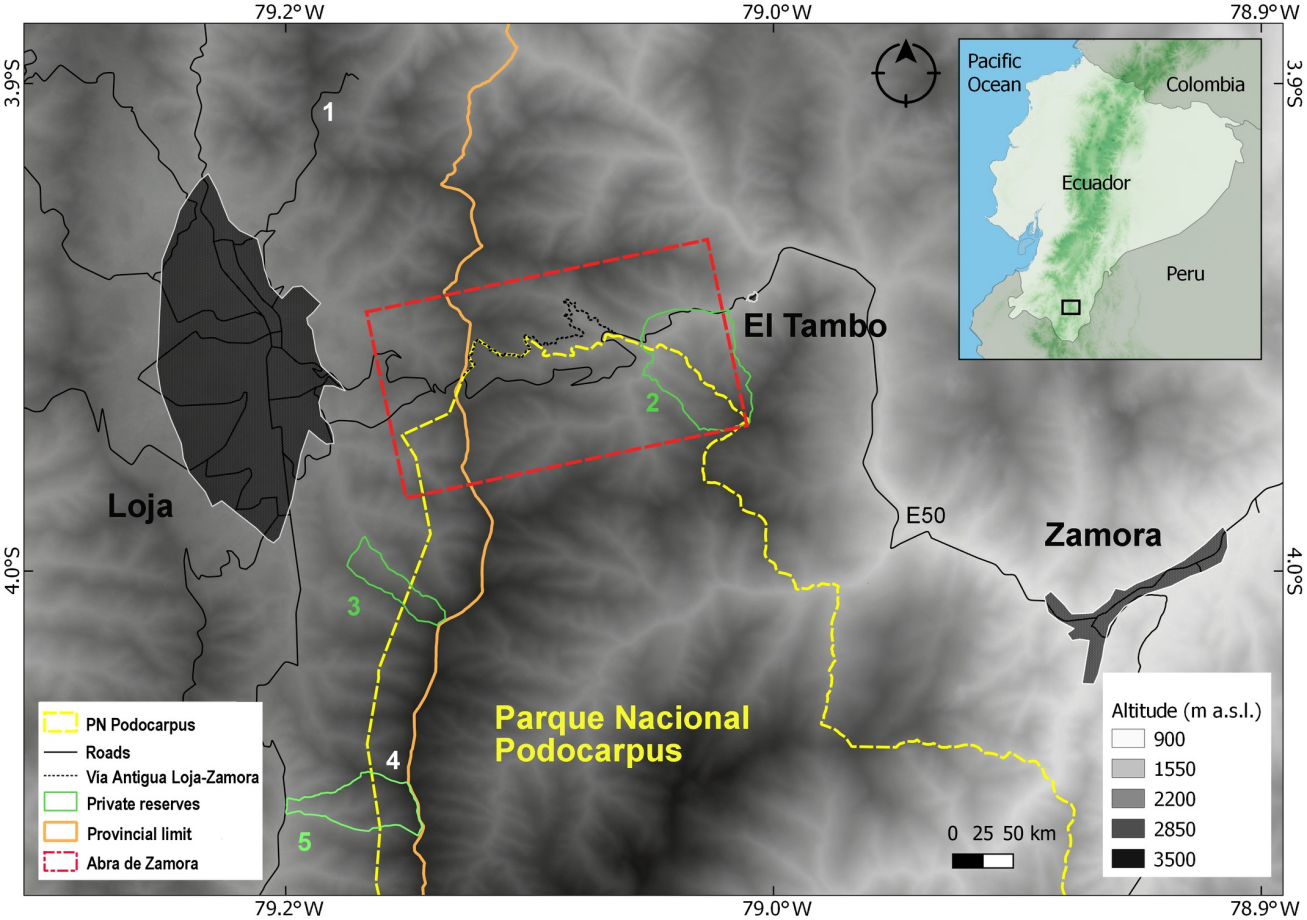
Abra de Zamora and nearby study sites. 1. Huacapamba, 2. Reserva Biológica San Francisco, 3. Reserva Madrigal del Podocarpus, 4. Parque Nacional Podocarpus (Cajanuma sector), 5. Reserva El Cristal.

**Fig 2:**
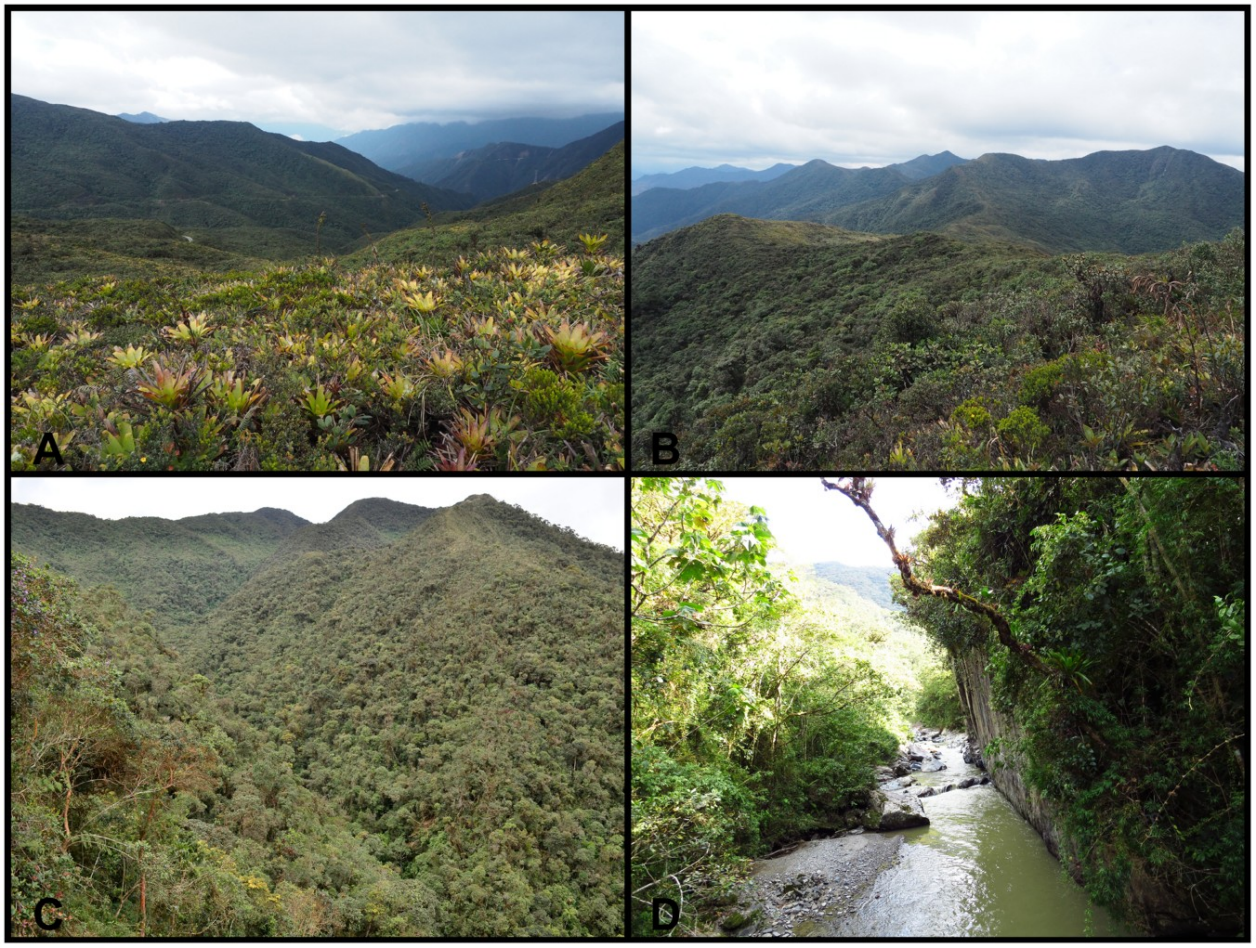
Abra de Zamora. **A**. General view of the higher part of Abra de Zamora (2800 m a.s.l.) with subpáramo ecosystem in the foreground. These terrestrial bromeliads are the type of microhabitat where *Gastrotheca psychrophila* specimens were collected in the ‘60s and '70s. In the background the old road that connected Loja with Zamora (Vía Antigua Loja-Zamora) is visible. **B**. Subpáramo ecosystem of the higher part (2750 m a.s.l.), habitat of *Pristimantis balionotus*, *P*. *colodactylus*, *P*. *matildae* sp. nov., *P*. *samaniegoi* sp. nov., and *P*. *versicolor*. **C**. Evergreen upper montane forest ecosystem (2600 m a.s.l.), habitat of *P*. *andinognomus*, *P*. *atratus*, *P*. *cryptomelas*, *P*. *samaniegoi* sp. nov., and *P*. *versicolor*. **D**. Evergreen lower montane forest ecosystem (1850 m a.s.l.), habitat of *Bolitoglossa* sp., *Rhinella margaritifera*, *Gastrotheca testudinea*, *P*. *galdi* and an undescribed *Pristimantis* species.

The name “Abra de Zamora” was used by William E. Duellman and John D. Lynch in their '70s publications [8, 9], to designate the “ridge (or crest) between Loja and Zamora Chinchipe provinces in the Cordillera Oriental in southern Ecuador” or just the area “13–15 kilometers east (by road) of Loja City, Ecuador”. The term Abra is used in orography to refer to the depression in a line of mountain peaks, which generally, for logistical reasons, is used to build the roads that cross the mountain range [10]; this is also the case of the road connecting two Ecuadorian cities from the tropical Andean region, Loja and Zamora. However, this name was not used by the local residents, who refer to the area between the two provinces closer to Loja as “El Tiro” (the Tiro Pass), and to the lower parts, close to El Tambo sector as “Los Trigales” (the Wheat Fields). Duellman and Lynch conducted their pioneer expeditions in search of amphibians on the old road that connected Loja with Zamora at that time (or Vía Antigua Loja-Zamora); this road was used only until the mid '80s, when a new road (the E50 highway) was constructed on the other side of the valley, and is still in use today (Figs 1 and 2A).

Between 1938 and 2010, eleven species of frogs were described from Abra de Zamora. The first species, *Lynchius flavomaculatus*, was described in 1938 by Hampton Wildman Parker [11], with specimens collected by the Ecuadorian naturalist, Clodoveo Carrión Mora. In 1974, William E. Duellman described *Gastrotheca psychrophila* [8], based on specimens collected in 1968 and 1971. John D. Lynch described six *Pristimantis* species from Abra de Zamora in 1979 [9], with specimens collected in 1968, 1971 and 1975: *P*. *balionotus*, *P*. *colodactylus*, *P*. *cryptomelas*, *P*. *percultus*, *P*. *versicolor* and *P*. *vidua*. Linda Trueb described *Telmatobius cirrhacelis* in 1979, using specimens collected in 1975 [12]. Paratypes collected from Abra de Zamora were used for the description of an additional two species: *P*. *andinognomus* [13], with specimens collected in 2003, and *Atelopus podocarpus* [14], with specimens collected in 1968 by Lynch and 1975 by Duellman.

Due to its location (Huancabamba depression, Andes), Abra de Zamora has been a center of endemism and probably a center of amphibian diversification [15]. From this list of eleven species originally described from this area, six species are strictly endemic to the area. An additional five species are currently undescribed, and similarly have not yet been encountered outside the area. This information emphasizes the importance of the region as a center of endemism, and suggests its role as a center of amphibian diversification, particularly for some groups of Terrarana. The advancement of molecular techniques allows precise species identification, which is especially important in areas of diversification and for cryptic species [16].

Unfortunately, many of these species were not encountered after their original description, and DNA samples were available only for a couple of them (*G*. *psychrophila*, *P*. *andinognomus*, *P*. *cryptomelas* and *P*. *versicolor*) which made their phylogenetic relationships impossible to assess. After almost 50 years of the important work of Duellman and Lynch we evaluated the presence and conservation status of the amphibian populations from this important hotspot. Through an intense fieldwork encompassing Abra de Zamora and several other potentially similar habitats in southern Ecuador, our study targets the following aspects: i. redescribe the species which were described from Abra de Zamora, including their genetic delimitation and advertisement call; ii. present the updated amphibian species list of Abra de Zamora with the description of two additional *Pristimantis* species; iii. search for their presence in similar montane habitats in surrounding areas from southern Ecuador, in an attempt to more precisely evaluate their distribution; and iv. update recommendations regarding their conservation status.

## Materials and methods

### Ethics statement

This study was carried out in strict accordance with the guidelines for use of live amphibians and reptiles in field research compiled by the American Society of Ichthyologists and Herpetologists, the Herpetologists’ League and the Society for the Study of Amphibians and Reptiles. Research permit was issued by the Ecuadorian Ministry of Environment (MAE-DNB-CM-2015-0016). This study was evaluated and approved by the Ethics Committee of Universidad Técnica Particular de Loja (UTPL-CBEA-2016-001).

### Specimen collection and study site

We focused our study on all the species described from Abra de Zamora. Even though there are no exact limits to the area, as a starting point, we used Abra de Zamora as referenced by Duellman and Lynch—the area between Loja and El Tambo longitudinally crossed by Vía Antigua Loja-Zamora and the E50 highway, which connects Loja and Zamora cities (Fig 1). Based on that, we defined our study site as an approximately 50 km^2^ area which includes all points of collection for amphibian species described from Abra de Zamora, along with neighboring favorable habitats. Almost half of this area is inside the Parque Nacional Podocarpus, basically delimiting the park’s northern border. Parque Nacional Podocarpus was declared officially in 1982 and comprises an area of 146,289 ha. The national park has a very irregular topography covering altitudes from 900 to 3650 m, with large areas of diverse natural habitats [17] and it is an important part of the Tropical Andes hotspots, the richest in biodiversity on the planet [18, 19]. The study site has an altitudinal range between 1700 and 3000 m a.s.l. and consists of *evergreen upper montane forest* (on the western slope, near Loja, up to 2700 m), *subpáramo* or *evergreen elfin forest* (on the crest, between 2700 and 3000 m), *evergreen upper montane forest* (on the eastern slope, between 2700 and 2100 m), and *evergreen lower montane forest* (on the eastern slope, near El Tambo, bellow 2100 m [20]) (Fig 2). In the study area, the average annual precipitation is around 4865 mm [21] and the average annual temperature around 7.3 °C [22].

Fieldwork was carried out between January 2016 and March 2020 (a total of 97 days) in Abra de Zamora and in several neighboring areas: Huacapamba (about 10 km to the north; 3.9061° S, 79.1909° W; datum WGS84), Reserva Madrigal del Podocarpus (about 9 km to the south; 4.0595° S, 79.1632° W) and the Cajanuma sector of Parque Nacional Podocarpus (about 13 km south to Abra de Zamora; 4.1163° S, 79.1719° W) (Fig 1). Additional specimens were collected from the wetland complex of Oña, Nabón, Saraguro and Yacuambi (3.5810° S, 79.0939° W), Bosque Protector Washapamba (3.6615° S, 79.2738° W), Ramos Urcu (at about 5 km west to San Lucas; 3.7111° S, 79.3019°), Reserva Tapichalaca (4.4859° S, 79.1477° W), and Reserva Cerro Plateado (4.6565° S, 78.8699° W) in Loja and Zamora Chinchipe provinces. We made intensive visual encounter surveys and auditory surveys both during the day and during the night (12h00–02h00).

Due to the heterogeneity of the surveyed ecosystems and sampling methods, we could not calculate the relative abundance or the survey effort. However, we use the following terms in ranking the relative abundance (adapted from [23, 24]): *common*: when the species presence was detected (seen or heard), in the proper habitat, in large or moderate numbers, on 50–100% of the sampling days/nights; *uncommon*: when the species presence was detected in moderate or small numbers, on 25–50% of the sampling days/nights; *rare*: when the species presence was detected in small numbers on less than 25% of the sampling days/nights.

Collected specimens were photographed alive, after which they were euthanized using 20% benzocaine, fixed in 10% formalin, and stored in 70% ethanol. Tissue samples for genetic analyses were preserved in 96% ethanol. Examined specimens (listed in the type-series and S1 Appendix) are housed in Museo de Zoología, Universidad Técnica Particular de Loja, Loja, Ecuador (MUTPL), Museo de Zoología de la Universidad del Azuay, Cuenca, Ecuador (MZUA), Museo de Zoología, Pontificia Universidad Católica del Ecuador, Quito, Ecuador (QCAZ) and Museo de Zoología, Universidad Tecnológica Indoamérica, Quito, Ecuador (MZUTI).

### Morphology

The description of qualitative and quantitative morphological characters, as well as the format of the description follows Duellman and Lehr [25]. Sex was determined by the presence of vocal slits and/or by gonadal inspection. Color data in life were based on field notes and digital photos. The specimens were weighted (body mass: BM) before euthanasia using a My Weigh Triton T3 portable scale with 0.01 g precision. Measurements were taken under a stereo microscope, with a Vernier caliper, and rounded to the nearest 0.1 mm. Specimens were measured for the following morphometric variables: (1) snout-vent length (SVL), distance from the tip of snout to posterior margin of vent; (2) head width (HW), widest portion of the head, measured at level of jaw articulation; (3) head length (HL), distance from the tip of snout to posterior angle of jaw articulation; (4) interorbital distance (IOD), distance between the inner margins of the orbits; (5) internarial distance (IND), distance between the inner edges of the narial openings; (6) upper eyelid width (EW), the perpendicular distance to the outer edge of the eyelid; (7) eye diameter (ED), distance between anterior and posterior borders of eye; (8) eye-nostril distance (EN), distance from posterior margin of nostril to anterior margin of eye; (9) tympanum diameter (TD), horizontal distance between peripheral borders of tympanic annulus; (10) femur length (FL), length of femur from vent to knee; (11) tibia length (TL), length of flexed leg from knee to heel; (12) foot length (FoL), distance from proximal margin of inner metatarsal tubercle to tip of Toe IV; (13) hand length (HaL), distance from proximal edge of palmar tubercle to the tip of Finger III. Measurements are given as mean ± SD.

### DNA extraction, amplification and sequencing

DNA extraction was performed directly from 96% ethanol-preserved liver tissue, using the Extract-N-Amp^™^ Tissue PCR Kit (Sigma-Aldrich, Merck KGaA, Darmstadt, Germany), followed by PCR reactions under the manufacturer’s protocol. Additionally, in the case of hard to identify and potentially endangered groups (especially *Gastrotheca* spp.), species identity was confirmed by non-destructive technique, using buccal swabs for DNA extraction [26]. Individuals were afterwards released at capture site. The *12S* and *16S* rRNA mitochondrial genes, and the nuclear *RAG-1* gene were amplified using the primers and annealing temperatures given in S1 Table. Success of PCR amplification was tested by gel electrophoresis, using a 1% agarose gel, stained with GelRed^™^ Nucleic Acid Gel Stain (Biotium Inc., Landing Parkway, CA). PCR products were purified using 1.1 volumes of PEG (20% Polyethylene glycol 8000, 2.5 M NaCl), followed by incubation at 37°C for 15 min. Samples were then centrifuged at 14,000 rpm at room temperature for 15 min, and afterwards the supernatant was discarded. The DNA pellet was washed twice with ice cold 80% ethanol, spinning the samples at maximum speed in the centrifuge for 2 min between ethanol washings. The ethanol was discarded, and the DNA pellet was dried at room temperature for 5 min or until no trace of ethanol was visible in the microcentrifuge tube. DNA was then resuspended in 15 μL sterile ddH_2_O. Amplicons were sent for sequencing at Macrogen Sequencing Service (Macrogen Inc., Seoul, Korea), using the corresponding primers for each gene. The newly generated DNA sequences were deposited in GenBank (S2 Table).

### DNA sequence analyses

Molecular data were analyzed using sequences of two mitochondrial genes (*12S* and *16S* rRNA) and one nuclear gene (*RAG-1*) from 123 individuals of 56 species from 55 different localities from Ecuador and Peru (S2 Table). We used all the GenBank-available sequences for *Lynchius* [27, 28], *Pristimantis orestes* group [16, 29], *Huicundomantis* subgenus of *Pristimantis* [30], and 129 new sequences (for 13 species) generated by our study (S2 Table). As outgroups we used *Oreobates amarakaeri*, *O*. *cruralis*, *Phrynopus bracki*, *P*. *juninensis*, *Niceforonia brunnea*, *N*. *dolops*, *Bryophryne cophites*, *Barycholos pulcher*, *Noblella heyeri*, and *Strabomantis anomalus* (for the *Lynchius* tree), *Pristimantis unistrigatus*, *P*. *ceuthospilus*, *P*. *imitatrix*, *P*. *diadematus*, *P*. *rhodoplichus*, *P*. *melanogaster*, *P*. *wiensi*, and *P*. *galdi* (for the *P*. *orestes* group) and the same *Pristimantis* species + *P*. *simonsii*, *P*. *orestes* and *P*. *colodactylus* for the *Huicundomantis* subgenus.

The sequences were edited, assembled and aligned [MAFFT algorithm, 31] using the program Geneious Prime (Biomatters Ltd.). The edited alignments of *12S*, *16S* and *RAG-1* sequences were concatenated into a single matrix, which was then used for the phylogenetic analyses. The phylogenetic analyses were based on a 2925 bp dataset for *Lynchius* (1028 bp *12S*, 1285 bp *16S* and 612 bp *RAG-1*), a 2683 bp dataset for *Pristimantis orestes* group (964 bp *12S*, 1077 bp *16S* and 642 bp *RAG-1*) and a 2463 bp dataset for *Huicundomantis* (905 bp *12S*, 915 bp *16S* and 643 bp *RAG-1*). The aligned matrices are available at https://doi.org/10.5281/zenodo.3952997.

Molecular phylogenetic relationships were inferred using Maximum Likelihood (ML) and Bayesian Inference (BI). We used PartitionFinder v. 2.1.1 [32] to select the best-fit models of sequence evolution and the best partition scheme with the AICc (for ML) and BIC (for BI) models of selection. ML analyses were conducted in GARLI v. 2.1 [33] performing four independent searches (two with the “streefname” set to random and two set to stepwise), with 250 replicates each, and with the “genthreshfortopoterm” set to 100,000. Node support was assessed with non-parametric bootstrapping [34] with 1,000 pseudoreplicates. The 50% majority rule consensus for the bootstrap trees was obtained with Geneious Prime (Biomatters Ltd.). BI analyses were conducted with MrBayes 3.2.6 [35], the Markov chain Monte Carlo runs being performed twice, independently, for 50 million generations, with a sampling frequency of 1000. Convergence of the runs was assessed from the average split frequency of standard deviations (*p* < 0.001) and by checking the potential scale reduction factors (PSRF ~ 1.0) for all model parameters. The first 25% of the trees were discarded as burn-in and the remaining ones were used to generate a 50% majority rule consensus tree, as well as to estimate the Bayesian posterior probabilities. Throughout the text, we considered that a node has “strong support” when its bootstrap value was > 70 and its Bayesian posterior probability was > 0.95, “moderate support” for 50–70 and 0.90–9.95 and “weak support” or non-resolved for lower values of 50 or 0.90, respectively. Uncorrected *p*-genetic distances for gene 16S were estimated with software MEGA6 [36] (S3 Table).

### Call recordings and analysis

We analyzed 16 calls of 15 individuals belonging to five species: release call of *L*. *flavomaculatus* and advertisement calls of *P*. *vidua*, *P*. *samaniegoi* sp. nov., *P*. *balionotus* and *P*. *versicolor*. The calls were recorded in the field or in laboratory (*L*. *flavomaculatus*) using an Olympus LS-11 Linear PCM Recorder and a RØDE NTG2 condenser shotgun microphone at 44.1 kHz sampling frequency and 16-bit resolution, in WAV file format. Air temperature and humidity were measured with a data logger (Lascar Electronics, model EL-USB-2-LCD, accuracy: ± 0.5°C; ± 5%). All analyzed call recordings are deposited in original form, full length at Fonoteca UTPL (record IDs are provided in S4 Table). Acoustic analysis was conducted using Raven Pro 1.6 (Center for Conservation Bioacoustics 2019). We measured the temporal parameters from the oscillograms and the spectral parameters from spectrograms obtained with the Hanning window function, DFT: 512 samples, 3 dB filter bandwidth: 124 Hz, and a 50% overlap.

The terminology and procedures for measuring call parameters follow Cocroft and Ryan [37], Toledo et al. [38] and Köhler et al. [39] and a call-centered approach was used to distinguish between a call and a note [sensu 39]. The following temporal and spectral parameters were measured and analyzed: (1) *call duration*: time from the beginning to the end of a call; (2) *inter-call interval*: the interval between two consecutive calls, measured from the end of one call to the beginning of the consecutive call; (3) *call rate*: number of calls per minute, measured as the time between the beginning of the first call and the beginning of the last call; (4) *note duration*: the duration of a single note within a call, measured from beginning to the end of the note; (5) *inter-note interval*: the interval between two consecutive notes within the same call, measured from the end of one note to the beginning of the consecutive note; (6) *note rate*: number of notes per second, measured as the time between the beginning of the first note and the beginning of the last note; (7) *number of pulses*: the number of pulses per note; (8) *pulse rate*: number of pulses per second, measured as the time between the beginning of the first pulse and the beginning of the last pulse; (9) *dominant frequency*: the frequency containing the highest sound energy, measured along the entire call; and (10) *the 90% bandwidth*, reported as *frequency 5%* and *frequency 95%*, or the minimum and maximum frequencies, excluding the 5% below and above the total energy in the selected call.

### Nomenclatural acts

The electronic edition of this article conforms to the requirements of the amended International Code of Zoological Nomenclature, and hence the new names contained herein are available under that Code from the electronic edition of this article. This published work and the nomenclatural acts it contains have been registered in ZooBank, the online registration system for the ICZN. The ZooBank LSIDs (Life Science Identifiers) can be resolved and the associated information viewed through any standard web browser by appending the LSID to the prefix "http://zoobank.org/". The LSID for this publication is: urn:lsid:zoobank.org:pub:32448ACE-C45F-4D84-9C2A-670E0C29B389. The electronic edition of this work was published in a journal with an ISSN, and has been archived and is available from the following digital repositories: PubMed Central and LOCKSS.

## Results

Class Amphibia Linnaeus, 1758

Order Anura Fischer von Waldheim, 1813

Family Bufonidae Gray, 1825

Genus ***Atelopus*** Duméril and Bibron, 1841

***Atelopus podocarpus*** Coloma, Duellman, Almendáriz, Ron, Terán-Valdez, and Guayasamin, 2010

**Common English name**. Podocarpus Harlequin Frog

**Common Spanish name**. Jambato de Podocarpus

**Etymology**. The specific name is a noun in apposition and refers to the Podocarpus National Park.

**Holotype**. QCAZ 6801, adult female, from Lagunas del Compadre (ca. 3300 m), Parque Nacional Podocarpus, Provincia Loja, Ecuador, collected on 1 December 1994 by Jenny Rudston.

**Paratypes**. EPN 1223–26, from Lagunas del Compadre (3400 m), Parque Nacional Podocarpus, Provincia Loja, Ecuador, no collection date stated on field notes, but possibly collected on September 1981, by Luis H. Albuja V.; EPN 2143–45, from Cerro Toledo (2900 m), Provincia Loja, Ecuador, collected on 11 September 1981 by Luis H. Albuja V.; EPN 3235, 3237, Mirador path nearby the Centro Administrativo Cajanuma of the Parque Nacional Podocarpus (2850–2990 m), Provincia Loja, Ecuador, obtained on 16–18 November 1986 by Ana Almendáriz; KU 120389, from 15 km east of Loja (2700 m), Provincia Zamora Chinchipe, Ecuador, obtained on 10 June 1968 by John D. Lynch; KU 165004–5, from 13 km east of Loja, Abra de Zamora, Provincia Loja, Ecuador, obtained by William E. Duellman; KU 178418, from Provincia Zamora Chinchipe: 13.7 km east of Loja, Provincia Loja, Ecuador; KU 201115–7, from 3.7 km south of Saraguro (2800 m), collected on 08 March 1984 by J. E. Simmons; QCAZ 2838 (cleared and double stained skeleton), from Centro Administrativo Cajanuma (2800 m), Parque Nacional Podocarpus, obtained on 25 June 1987 by Luis A. Coloma, Mario García Saltos, and Renato León.

**Remarks**. Despite numerous searching efforts carried out by different teams from various institutions (including us), in the last several years, in Abra de Zamora and nearby areas, the species was not re-encountered. The last sighting of *Atelopus podocarpus* dates from 1994, from Parque Nacional Podocarpus (Lagunas del Compadre), and the species is considered probably extinct.

Family Hemiphractidae Peters, 1862

Genus ***Gastrotheca*** Fitzinger, 1843

***Gastrotheca psychrophila*** Duellman, 1974

*Gastrotheca* (*Duellmania*) *psychrophila*: Dubois, 1987

*Gastrotheca* (*Gastrotheca*) *psychrophila*: Duellman, 2015

**Common English name**. Ridge Marsupial Frog

**Common Spanish name**. Rana marsupial del Abra de Zamora

**Etymology**. The specific name is derived from the Greek *psychros*, meaning “cold,” and the Greek *philos*, meaning “having an affinity for”; the name was used in allusion to the cold climate at the type locality [40].

**Holotype**. KU 120760, an adult female, from the ridge between Loja and Zamora, 2850 m, 13–14 km E (by road) of Loja, Provincia Loja, Ecuador; obtained on 10 June 1968 by John D. Lynch.

**Paratypes**. Collected at the type locality: KU 120761, 10 June 1968, John D. Lynch; KU 141586, 21 May 1971, Richard R. Montanucci; KU 142631–7, 21–23 July 1971, William E. Duellman.

**Remarks**. Despite intensive searches carried out since 2016, we were not able to find this species. We managed to survey an area of almost 100 km^2^ around the type locality, but unfortunately, we were not able to find any *G*. *psychrophila* individuals. The results are even more worrisome as our intensive searches in the type locality failed to find any traces of marsupial frog presence (no tadpoles or calls), although if the ecosystem remained practically unchanged since the species was found in 1968 (Fig 2A). Two other *Gastrotheca* species were encountered, relatively close to the type locality: *G*. *elicioi* (on the western slope) and *G*. *testudinea* (on the eastern slope), but both well below *G*. *psychrophila*’s altitudinal range of 2700–2800 m. The identity of the encountered *Gastrotheca* species was confirmed through non-destructive DNA tissue sampling (using buccal swabs). In the light of these current findings, we are considering *G*. *psychrophila* probably extinct.

Superfamily Brachycephaloidea Günther, 1858

Family Strabomantidae Hedges, Duellman, and Heinicke, 2008

Subfamily Pristimantinae Pyron and Wiens, 2011

Genus ***Lynchius*** Hedges, Duellman, and Heinicke, 2008

### Phylogeny

**Fig 3:**
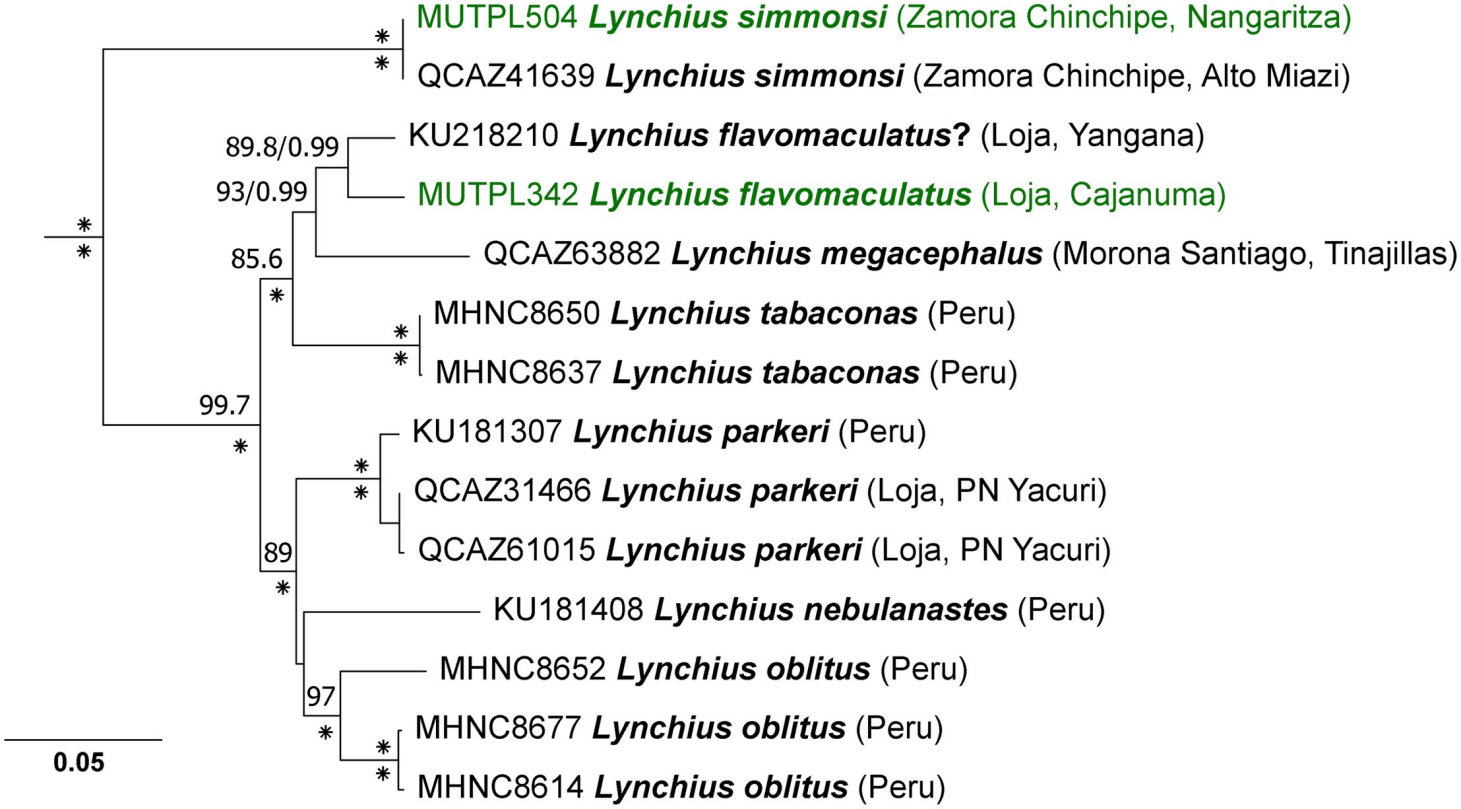
Maximum likelihood phylogram of the *Lynchius* species based on 2925 base pairs of concatenated DNA from *12S*, *16S*, and *RAG-1* gene fragments. Bootstrap values (%) and Bayesian posterior probabilities (decimal) are shown except when they are below 50 (bootstrap) or 0.5 (posterior probability); asterisks indicate support values of 100% or 1 (posterior probabilities). Outgroup is not shown; the tree was routed with *Strabomantis anomalus*. With green are marked the newly generated sequences for the present study. The collection number, species name, province and short locality name (for the Ecuadorian samples) or country (for the Peruvian samples) of the samples are shown next to each terminal (associated data listed in S2 Table).

For *Lynchius*, PartitionFinder under AICc (for ML) identified five partition schemes as the best strategy (best model in parentheses): *12S* (GTR+I+G), *16S* (GTR+I+G), *RAG-1* 1^st^ position (TRNEF+G), *RAG-1* 2^nd^ position (K81UF+G) and *RAG-1* 3^rd^ position (GTR+G). Under BIC (for BI) PartitionFinder identified three partitions: *12S* and *16S* (GTR+I+G), *RAG-1* 1^st^ position (K80+G) and *RAG*-1 2^nd^ and 3^rd^ positions (HKY+G). The phylogenetic trees constructed by Bayesian inference and Maximum likelihood showed the same topology, with the only difference being the position of *L*. *nebulanastes*, which remained non-resolved in both trees (Fig 3). The phylogram of our analysis showed exactly the same topology with the one constructed by Sanchez-Nivicela et al. [28].

The *L*. *flavomaculatus* sequence (KU 218210) used in all published studies (e.g. [27, 28, 41, 42]), was obtained from a specimen collected in 1990, from 19.4 km south of Yangana, Loja province, which is located more than 60 km south from the type locality, Abra de Zamora. In June 2018 we encountered an adult female (MUTPL 342) in Parque Nacional Podocarpus (Cajanuma entrance), from about 13 km south of the type locality, and in the same type of ecosystem as of the type locality (shrub páramo). The uncorrected *p*-genetic distance for the gene *16S* between the two samples is 3.1% (S3 Table). Based on the conservative 3% threshold of the genetic distances in the *16S* gene for species delineation [43], together with observed morphological differences between several similar species from the nearby areas (pers. obs.) we consider the specimen from Yangana as belonging to a different, currently undescribed species. Thus, it seems that the previously known *L*. *flavomaculatus* is in fact a complex of cryptic species, composed of at least four different species in Ecuador: one species from Yangana, *L*. *flavomaculatus* sensu stricto from the type locality (Abra de Zamora), one species from San Lucas and Saraguro (we are preparing its description) and one species nearby Plan de Milagro, Morona Santiago province. In addition, very likely the specimen collected in Peru [25] is a different, currently undescribed species.

***Lynchius flavomaculatus*** (Parker, 1938)

(Fig 4)

**Fig 4:**
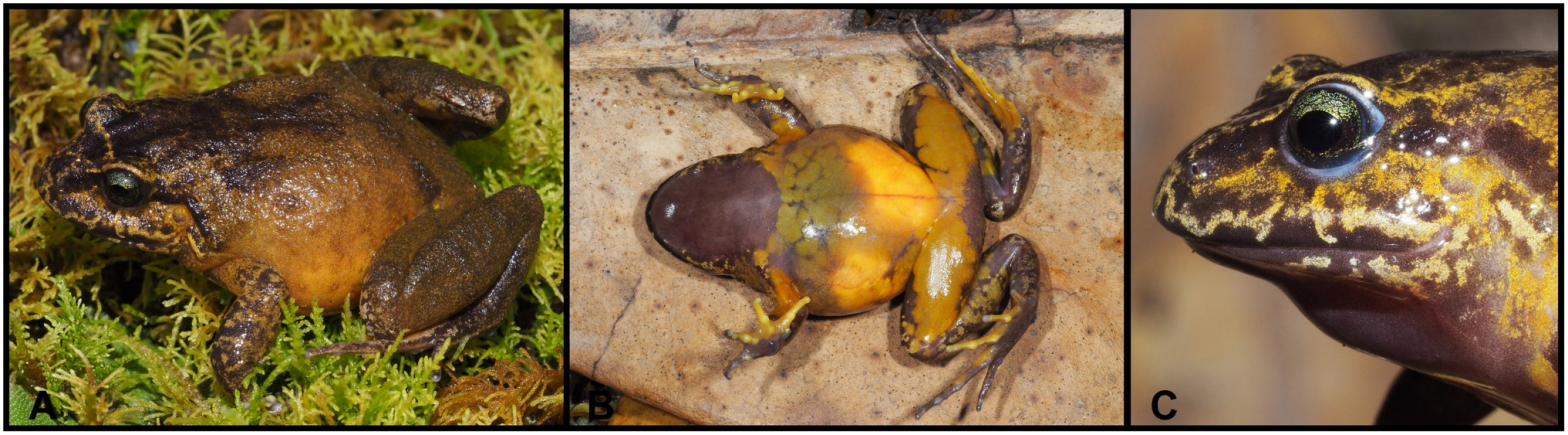
*Lynchius flavomaculatus* (MUTPL 342, adult female) in life. **A**. Dorsolateral view; **B**. Ventral view; **C**. Profile view of the head.

*Eleutherodactylus flavomaculatus* Parker, 1938

*Niceforonia flavomaculata*: Lynch, 1969

*Phrynopus flavomaculatus*: Lynch, 1975

*Eleutherodactylus flavomaculatus*: Lehr, 2006

‘‘*Eleutherodactylus*” *flavomaculatus*: Heinicke, Duellman, and Hedges, 2007

*Lynchius flavomaculatus*: Hedges, Duellman, and Heinicke, 2008

**Common English name**. Yellow-spotted Andes Frog

**Common Spanish name**. Rana andina de manchas amarillas

**Etymology**. The specific name is derived from the Latin adjective *flavus*, meaning “yellow” and the Latin noun *maculatus*, meaning “spots” and refers to the yellow spots on the venter [25].

**Holotype**. BMNH 1947.2.16.11 (formerly 1935.11.3.16), adult female from 15 km East of Loja City, Ecuador, 3000 m, collected by C. Carrión.

**Paratypes**. BMNH 1947.2.16.12–14 (formerly 1935.11.3.17–20), a male and three immature females from the type locality.

**Diagnosis**. *Lynchius flavomaculatus* is a large species distinguished by the following combination of traits: (1) skin on dorsum shagreen (trait more visible in life) and of the flanks smooth; dorsolateral folds absent; low middorsal fold present (trait more visible in life); skin on venter smooth; discoidal and thoracic folds absent; (2) tympanic membrane and annulus distinct; large supratympanic fold obscuring upper and posterodorsal edges of tympanum; one large, elongated postrictal tubercle present; (3) snout short, rounded in dorsal view, slightly inclined anteroventrally in profile; canthus rostralis weakly concave in dorsal view, angular in profile; (4) upper eyelid without tubercles; low, cranial crests present; (5) dentigerous processes of vomers prominent, triangular, with 4 to 5 teeth; (6) males with vocal slits on each side of the tongue and no nuptial pads; (7) fingers long and slender; Finger I longer than Finger II; tips of digits narrow, not expanded, rounded; circumferential grooves absent; fingers lacking lateral fringes; (8) subarticular tubercles prominent, round; supernumerary palmar tubercles large, rounded, smaller than subarticular tubercles; palmar tubercle partially divided into a much larger (inner) and a smaller (outer) tubercles; thenar tubercle prominent, elliptical; (9) ulnar tubercles absent; (10) heel lacking tubercles; outer edge of tarsus lacking tubercles; inner edge of tarsus lacking tubercles or fold; (11) inner metatarsal tubercle elliptical, about 2x round outer metatarsal tubercle; subarticular tubercles prominent, subconical; supernumerary plantar tubercles absent; (12) toes long and slender; Toe V slightly shorter than Toe III; tips of digits narrow, not expanded, rounded; circumferential grooves absent; toes without lateral fringes; webbing absent; (13) in life, dorsum mottled tan, reddish or dark brown, dull greenish gray or yellow; flanks yellow with or without brown spots; venter mostly yellow, with or without gray on the throat; throat light gray with yellow blotches; yellow blotches on groin and anterior surfaces of thighs; iris pale green, with a brown median horizontal streak, and fine black reticulations; (14) SVL 34.6–44.2 mm in adult females (*n* = 31) and 24.0–35.2 mm in adult males (*n* = 27) [27] (data regarding the SVL contains individuals from several locations, and probably include specimens belonging to other, cryptic, currently undescribed species than *L*. *flavomaculatus*).

**Advertisement call**. Unknown.

**Fig 5:**
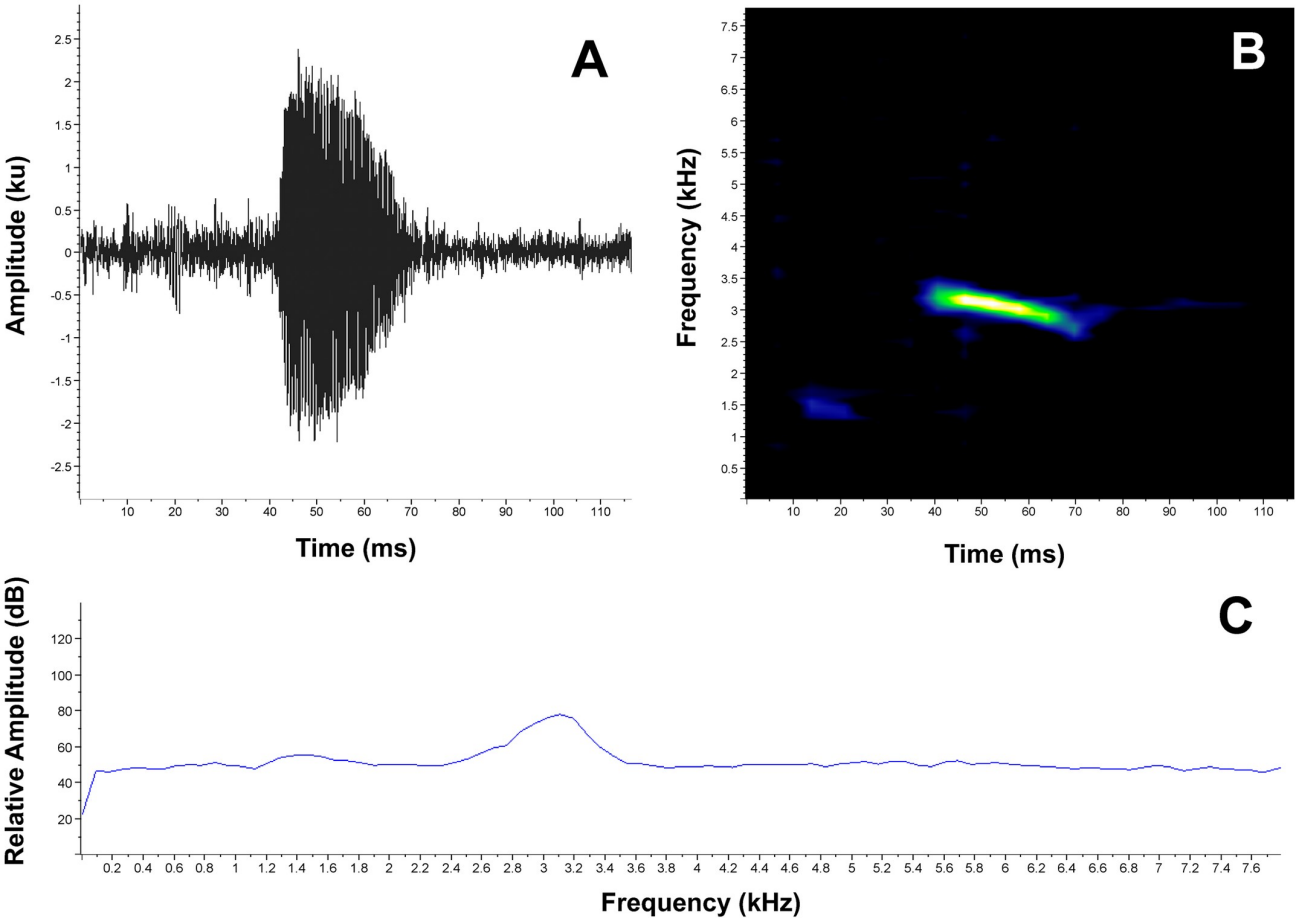
Release call of *Lynchius flavomaculatus* (MUTPL 342, FUTPL-A 246). **A**. Oscillogram of a single call; **B**. Spectrogram of a single call; **C**. Power spectrum of a single call.

**Release call**. When caught and manipulated, the female MUTPL 342 emitted low, short, screeching calls (Fig 5), which we interpreted as release calls (calls usually emitted during amplexus or after being touched by other males, when the amplexus is not desired by the amplected individual, [38]), and not distress calls (calls emitted during subjugation by a potential predator). We base this observation on the fact that the distress call of a similar, undescribed, *Lynchius* species is very different (long, loud shrieking sounds; pers. obs.). We recorded several of these calls in the laboratory, from a 20 cm distance, while holding the female by the sides of its body, in the axillary and inguinal regions, simulating an amplexus (S4 Table). The calls are single-pulsed notes (tonal sounds) characterized by a duration of (range and mean ± SD in parenthesis) 0.026–0.041 s (0.033 ± 0.006, *n* = 6), a dominant frequency of 2239.5–3100.8 Hz (2555.3 ± 343.088, *n* = 6) and a 90% bandwidth range of 2153.3–2842.4 Hz (2426.1 ± 295.4, *n* = 6) to 2325.6–3186.9 Hz (2641.4 ± 343.088, *n* = 6). The fundamental frequency and harmonics are not recognizable.

**Fig 6:**
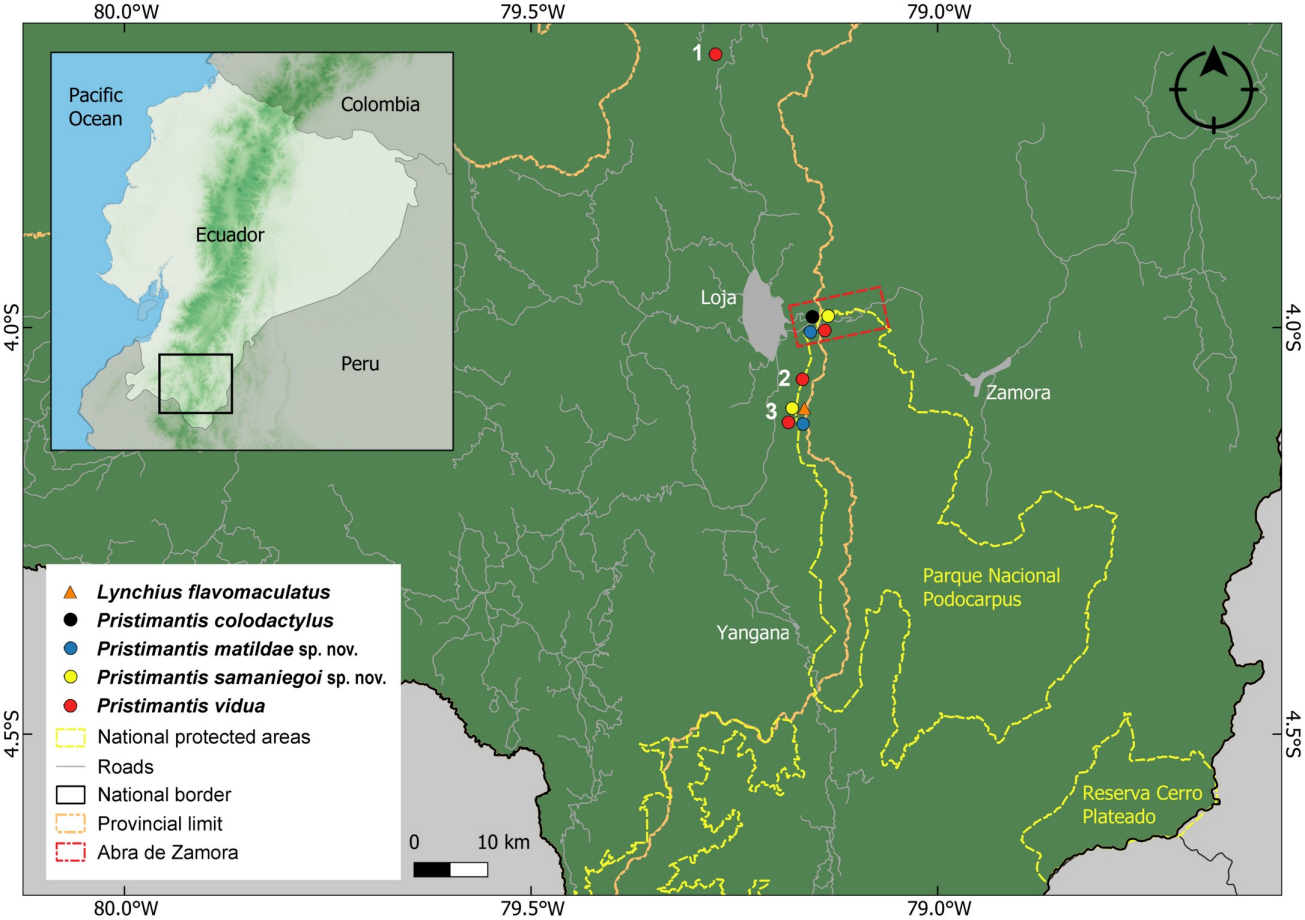
Distribution of *Lynchius flavomaculatus* and of the species from the *Pristimantis orestes* species group described from Abra de Zamora. Records are based on specimens (and DNA sequences) deposited at the Museo de Zoología, Universidad Técnica Particular de Loja (MUTPL). 1. Bosque Protector Washapamba, 2. Reserva Madrigal del Podocarpus, 3. Parque Nacional Podocarpus (Cajanuma sector).

**Distribution**. Currently, the presence of *L*. *flavomaculatus* is confirmed (with DNA samples) only from Parque Nacional Podocarpus (Cajanuma entrance), in a shrub páramo ecosystem [44], from about 13 km south to the type locality, Abra de Zamora, in Loja province (Fig 6). Despite intensive searches, we did not find any individuals in the type locality. Based on the available data we conclude that the specimen KU 218210 collected at 19.4 km south of Yangana, Loja province, is most likely a different, undescribed species. Also, the specimens collected at 8–9 km north to San Lucas or 10 km south of Saraguro, in Loja province [27], actually belong to a currently undescribed new species. As for the specimens from Morona Santiago province and Peru [25, 27] they represent very probable different, undescribed species, taking in consideration the large distances from the type locality. The species was encountered at an altitudinal range between 3000 and 3250 m a.s.l. in shrub páramo ecosystems.

**Natural history**. This species is considered terrestrial, usually found beneath rocks or logs in páramo and subpáramo habitats [45]. We found the female MUTPL 342 emerging from a thick moss bed, which is likely a preferred microhabitat for *Lynchius* species. From the type locality, there were collected 44 specimens between 1968 and 1984 (22 specimens in 1968, 7 in 1971, 14 in 1975, and 1 in 1984) by J.D. Lynch, R.R. Montanucci and W.E. Duellman, according to the Kansas University Herpetology Collections database (https://biodiversity.ku.edu/herpetology/collections). However, our ongoing fieldwork since 2016 in the type locality did not yield any specimens. It is possible that the population is experiencing a decline, but conclusive conclusions are hard to reach due to the species low detectability. The female MUTPL 342 had 31 large unpigmented eggs (SVL 42.5 mm, BM 8.21 g).

**Conservation status**. *Lynchius flavomaculatus* is currently listed as Data Deficient in view of the absence of recent information on its extent of occurrence, population status and ecological requirements [46]. Based on the available (revised) information we consider *L*. *flavomaculatus* to be Endangered following B1ab(iv)c(iii)+2ab(iv)c(iii) IUCN criteria [47] because: (1) its Extent of occurrence (EOO) and Area of occupancy (AOO) are estimated to be less than 50 km^2^; (2) it is known from only two close locations; and (3) we suspect a decline in the number of locations or subpopulations.

**Remarks**. The diagnosis provided herein is based on the thorough revision of Motta et al. [27] and one adult female specimen, MUTPL 342, collected from about 13 km south to the type locality. Our diagnosis concurs with most of the morphological features described; however we identified some differences, which we attribute to the fact that the specimens examined by the authors [27] belong to at least four different cryptic species. Also, the opportunity to examine a live specimen helped to complete the diagnosis by adding descriptions of characters which are evident in life, but lost in preserved specimens. The most important difference is the lack of discoidal and thoracic folds in the specimen collected from Cajanuma. Duellman and Lehr [25] list the discoidal fold as absent, but Motta et al. [27] consider that discoidal and thoracic folds are present. Specimen MUTPL 342 lacked both folds (in life as well as in preservative; Fig 4B). On the other hand, all the specimens collected by us nearby Saraguro and San Lucas have evident discoidal and thoracic folds, so it might be possible that this feature is characteristic for the individuals of this currently undescribed species. Similarly, we observed the skin on dorsum as shagreen, with a more granular texture on the back and dorsal surfaces of hindlimbs, but without tubercles (Fig 4A), and thus, in accordance to Duellman and Lehr [25], we did not observed any tubercles on the upper eyelids, either in life or in preservative (Fig 4C).

Genus ***Pristimantis*** Jiménez de la Espada, 1870

***Pristimantis orestes* species group** [sensu 6, 16, 48]. Three species of this species group were described from Abra de Zamora. We add our description of an additional two new species further below.

### Phylogeny

**Fig 7:**
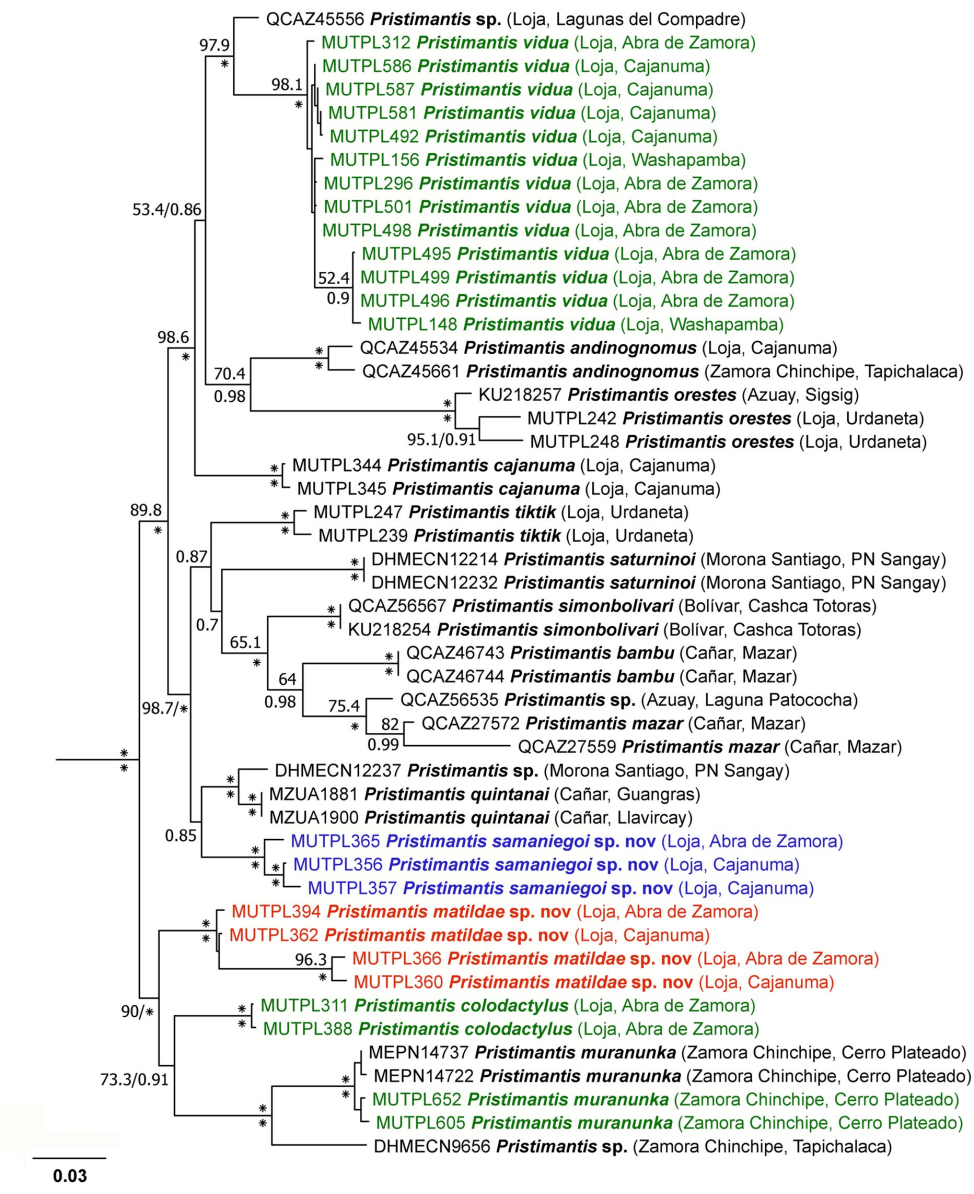
Maximum likelihood phylogram of the *Pristimantis orestes* species group based on 2683 base pairs of concatenated DNA from *12S*, *16S*, and *RAG-1* gene fragments. Bootstrap values (%) and Bayesian posterior probabilities (decimal) are shown except when they are below 50 (bootstrap) or 0.5 (posterior probability); asterisks indicate support values of 100% or 1 (posterior probabilities). Outgroup is not shown; the tree was routed with *Pristimantis galdi*. With green are marked the newly generated sequences for the present study; with blue and red are marked the newly described species. The collection number, species name, province and short locality name of the samples are shown next to each terminal; all samples are from Ecuador (associated data listed in S2 Table).

For the *Pristimantis orestes* species group, PartitionFinder under AICc (for ML) identified four partition schemes as the best strategy (best model in parentheses): *12S* (GTR+I+G), *16S* (GTR+I+G), *RAG-1* 1^st^ and 2^nd^ positions (F81+G), and *RAG-1* 3^rd^ position (TVM+G). Under BIC (for BI) PartitionFinder identified three partitions: *12S* and *16S* (GTR+I+G), *RAG-1* 1^st^ and 2^nd^ positions (F81+G), and *RAG-1* 3^rd^ position (K80+G). The phylogenetic trees constructed by Bayesian inference and Maximum likelihood showed exactly the same topology, but with overall stronger support in the case of the Bayesian inference (Fig 7).

Once again, we recovered the *P*. *orestes* species group as monophyletic, with strong support (bootstrap values = 100%; posterior probabilities = 1) in both ML and Bayesian analyses (Fig 7). Our analysis confirmed the membership of *P*. *vidua*, *P*. *colodactylus* and two new species, i.e. *P*. *samaniegoi* sp. nov. and *P*. *matildae* sp. nov., to the *P*. *orestes* species group. In general, the phylogenetic tree in our analysis showed the same topology with the one constructed by Urgilles et al. [16], but the addition of the new species to the group changed the kinship between some of the species. Also, differences appeared in the position of some unresolved branches such as *P*. *tiktik* or *P*. *saturninoi* (Fig 7).

Our phylogenetic tree reveals three, strongly supported, large subgroups of species: the *P*. *orestes* subgroup (with *P*. *andinognomus*, *P*. *orestes*, *P*. *vidua*, an undescribed species and *P*. *cajanuma*), the closely related *P*. *simonbolivari* subgroup (with *P*. *tiktik*, *P*. *saturninoi*, *P*. *simonbolivari*, *P*. *bambu*, *P*. *mazar*, *P*. *quintanai*, *P*. *samaniegoi* sp. nov and two undescribed species) and the morphologically distinct, bromeliad specialist, *P*. *colodactylus* subgroup (with *P*. *matildae* sp. nov., *P*. *colodactylus*, *P*. *muranunka* and an undescribed species).

***Pristimantis andinognomus*** Lehr and Coloma, 2008

**Common English name**. Andean Dwarf Robber Frog

**Common Spanish name**. Cutín nomo

**Etymology**. The specific name is derived from the New Latin noun *Andinus* meaning “Andes” and the New Latin noun *gnomus* meaning “dwarf” and refers to the small size of the species, which at the time of description was the smallest species of the genus [13].

**Holotype**. QCAZ 16695, an adult male, from Reserva Tapichalaca, on the road between Yangana and Valladolid (2667 m), Provincia Zamora-Chinchipe, Ecuador, collected by Italo G. Tapia and Queti M. Tapia on 17 September 2001.

**Paratypes**. 20 males: QCAZ 16669, 16672–74, 16685–88, 16690–93, 26326, 26329, 26331, 26343, 26347, 26352, 26964–65, 19 females: QCAZ 16670–71, 16675–78, 16680–84, 16689, 16694, 16697, 26325, 26330, 26351, 27033, 29248, 24 subadults: QCAZ 16670–72, 16674–78, 16680–94, 16697 collected at the type locality along with the holotype by Italo G. Tapia and Queti M. Tapia on 17 September 2001; QCAZ 26325–26, 26329–31, 26343, 26347, 26351–52, from Abra de Zamora (2800 m), boundary between Provincias Loja and Zamora Chinchipe, collected by Italo G. Tapia, Erik R. Wild, Andrés Merino-Viteri, and Luis A. Coloma on 16 August 2003; QCAZ 26964–65, 27033, from Reserva Tapichalaca (2400 m), on road between Yangana and Valladolid, collected by David Kizirian and Juan C. Santos on 30 October 2000; QCAZ 29248, collected by Carlos Carrión-Bonilla and Paolo Piedrahita-Piedrahita on 18 February 2001.

**Remarks**. It seems that the population of *Pristimantis andinognomus* from Abra de Zamora is a different species from the one from the type locality, Reserva Tapichalaca, and it needs a detailed revision. This species is common in the higher parts of Abra de Zamora, and, together with *P*. *versicolor*, represents the most abundant frog species in the area. The calls of the males were registered during each night of fieldwork at the site, regardless of weather conditions, and sometimes even during the day. More data is needed to clarify this species phylogenetic relationships and current distribution.

***Pristimantis vidua*** (Lynch, 1979)

(Fig 8)

**Fig 8:**
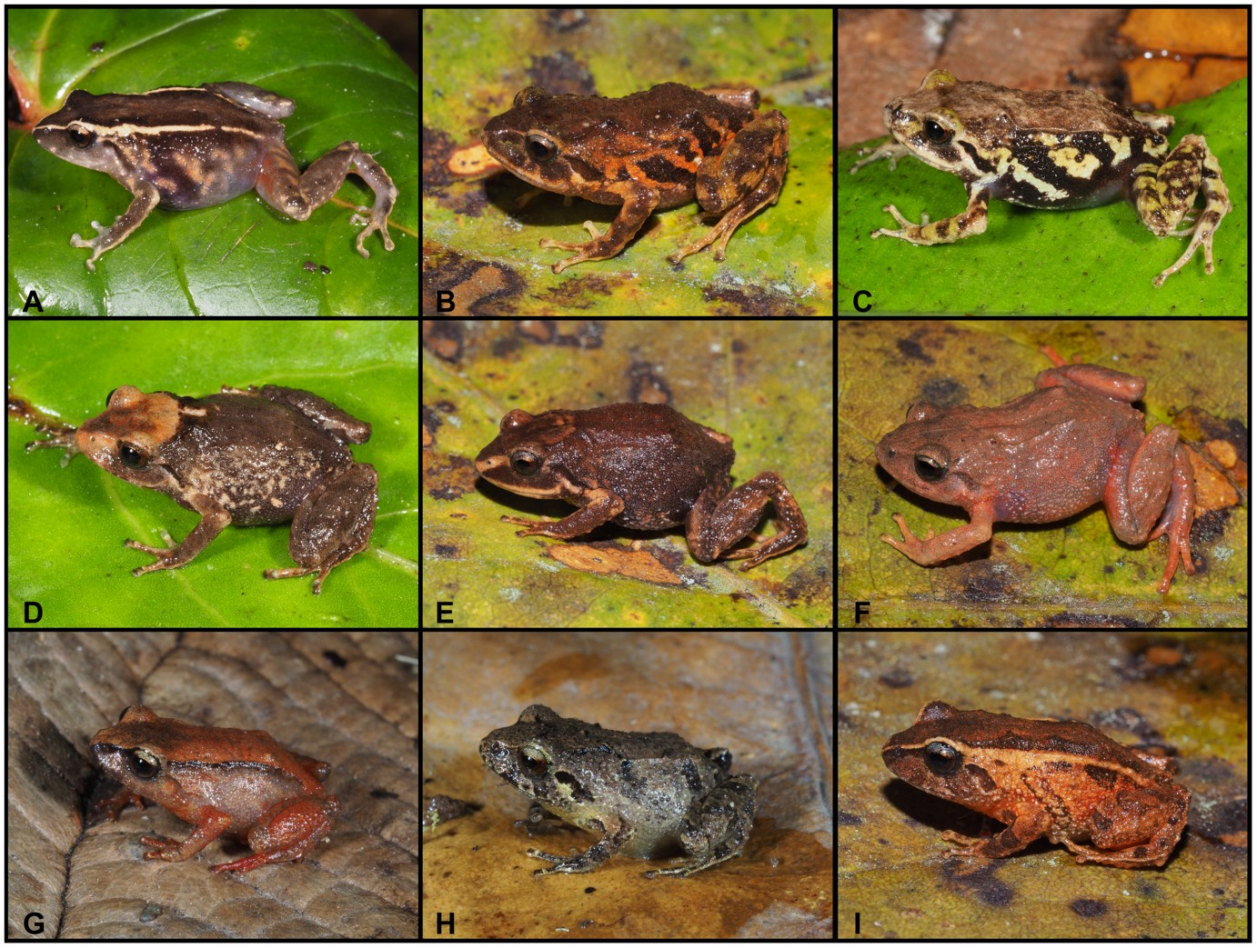
Color variation of *Pristimantis vidua* in life. **A–F** females: **A**. MUTPL 312, **B**. MUTPL 164, **C**. MUTPL 586, **D**. MUTPL 590, **E**. MUTPL 153, **F**. MUTPL 150; **G–I** males: **G**. MUTPL 495, **H**. MUTPL 496, **I**. MUTPL 148. Abra de Zamora (A, G, H), Bosque Protector Washapamba (B, E, F, I), Parque Nacional Podocarpus—Cajanuma (C, D). Most common coloration of this species is presented in A, G and H.

*Eleutherodactylus vidua* Lynch, 1979

*Eleutherodactylus* (*Eleutherodactylus*) *vidua*: Lynch, 1996; Lynch and Duellman, 1997

*Pristimantis vidua*: Heinicke, Duellman, and Hedges, 2007

*Pristimantis* (*Pristimantis*) *vidua*: Hedges, Duellman, and Heinicke, 2008

**Common English name**. Mountain Crest Robber Frog

**Common Spanish name**. Cutín viuda

**Etymology**. Latin, “a widow”, used as a noun in apposition. The choice of this name reflected the belief of the author that this species might be all-female [9].

**Holotype**. KU 120082, adult female obtained 15 km E Ciudad Loja, Provincia Zamora-Chinchipe, Ecuador, 2800 m, on 10 June 1968 by John D. Lynch.

**Paratypes**. KU 120083–88, KU 120090–91, topotypes; KU 141994, 15 km E Loja, Provincia Zamora-Chinchipe, Ecuador, 2710 m; KU 120089, 13–14 km E Loja, Provincia Loja, Ecuador, 2850 m; KU 120092, 8–9 km N San Lucas, Provincia Loja, Ecuador, 3000–3100 m.

**Diagnosis**. *Pristimantis vidua* is a small species distinguished by the following combination of traits: (1) skin on dorsum finely tuberculated (in life the tuberculated texture of the skin is more evident); skin on venter areolate; discoidal fold visible; dorsolateral folds present; low middorsal fold present; (2) tympanic membrane absent; ventral part of tympanic annulus visible, concealed by skin; its length about 43% of the length of eye; supratympanic fold present; (3) snout short, rounded in dorsal view and in profile; canthus rostralis weakly concave in dorsal view, rounded in profile; (4) upper eyelid bearing several small tubercles, similar in size and shape with the ones from the dorsum (trait more visible in life), about 61% IOD in females and 69% IOD in males; cranial crests absent; (5) dentigerous processes of vomers prominent, oblique, round or slightly ovoid, separated medially by distance lower than width of processes; each processes bearing 3 to 9 teeth; (6) males with a large subgular vocal sac and vocal slits; nuptial pads absent; (7) Finger I shorter than Finger II; discs on fingers slightly expanded, rounded; circumferential grooves present; fingers lacking lateral fringes; (8) subarticular tubercles prominent, round; supernumerary palmar tubercles evident; palmar tubercle completely divided into a larger (inner) and a smaller (outer) tubercles; thenar tubercle oval, smaller than the inner palmar tubercle; (9) small, inconspicuous, ulnar tubercles present (trait more visible in life); (10) heel with 2 or 3 larger and several smaller tubercles (trait more visible in life); outer edge of tarsus bearing a row of small tubercles; inner tarsal tubercles coalesced into a long tarsal fold; (11) inner metatarsal tubercle broadly ovoid, about 1.5 to 2x rounded outer metatarsal tubercle; supernumerary plantar tubercles present; (12) Toe V slightly longer than Toe III; discs on toes slightly expanded, rounded, about same size as those on fingers; circumferential grooves present; toes lacking lateral fringes; webbing absent; (13) in life, dorsum tan to brown or grey, mottled with darker brown or bearing dark dorsolateral stripes; venter cream or pinkish white; no markings in axilla, groin or on concealed limb surfaces; iris whitish gray, with a reddish broad median horizontal streak, that seems to cover all the lower part of the iris, and with fine black reticulations; (14) SVL 17.9–26.8 mm in adult females (22.4 ± 2.35 SD, *n* = 16) and 16.1–19.5 mm in adult males (17.6 ± 1.28 SD, *n* = 11).

**Variation**. Morphometric variation is shown in Table 1. The females are more colorful and variable than the males (Fig 8). The female specimen MUTPL 312 (Fig 8A) had a coloration very similar with the holotype KU 120082 and paratype KU 120089 (Fig 13D in [9]), with light orange dorsolateral stripes and spots and pale dorsolateral folds that continue with stripes onto the snout. Many females have particular dark dorsolateral stripes on lighter background (orange, yellow or cream), as in the case of MUTPL 164 and 586 (Fig 8B and 8C). One female, MUTPL 590, had the head and snout distinctly yellowish beige, contrasting with the rest of the body which was dark brown (Fig 8D). Other females are dark brown with light upper lips and canthal stripes (MUTPL 153, Fig 8E), or completely pinkish beige, as MUTPL 150 (Fig 8F). Most of the encountered males are similar in color with MUTPL 495, with reddish brown middorsal band and members, dark dorsolateral folds and lighter flanks (Fig 8G), or alternatively have a uniform grey coloration, with some darker stripes and black supratympanic stripes, as in MUTPL 496 (Fig 8H). One male, MUTPL 148 had a different coloration, with a brown middorsal band, and yellowish dorsolateral stripes that continued onto the snout and yellowish flanks (Fig 8I).

**Table 1:** Body mass (in grams), measurements (in mm) and morphological proportions (in percentages) of adult females and males of the species from the *Pristimantis orestes* species group described from Abra de Zamora.

Character	*Pristimantis vidua*		*Pristimantis samaniegoi* sp. nov.		*Pristimantis colodactylus*		*Pristimantis matildae* sp. nov.	
	females (*n* = 16)	males (*n* = 11)	females (*n* = 4)	males (*n* = 3)	females (*n* = 2)	males (*n* = 1)	females (*n* = 4)	males (*n* = 3)
Body mass (BM)	0.97 ± 0.29 (0.54–1.61)	0.49 ± 0.10 (0.35–0.66)	0.63 ± 0.15 (0.44–0.80)	0.34 ± 0.04 (0.31–0.38)	0.61–0.62	0.31	0.84 ± 0.16 (0.66–0.97)	0.55 ± 0.06 (0.50–0.61)
Snout-vent length (SVL)	22.4 ± 2.35 (17.9–26.8)	17.6 ± 1.28 (16.1–19.5)	19.3 ± 1.58 (17.1–20.7)	16.0 ± 1.17 (14.7–16.9)	20.2–20.5	16.9	22.5 ± 1.12 (20.8–23.3)	20.4 ± 0.68 (19.9–21.2)
Head width (HW)	8.2 ± 0.85 (6.9–10.1)	6.3 ± 0.54 (5.7–7.2)	6.6 ± 0.37 (6.1–7.0)	5.3 ± 0.47 (4.8–5.7)	6.5–6.9	5.4	8.1 ± 0.27 (7.7–8.3)	6.9 ± 0.35 (6.7–7.3)
Head length (HL)	7.4 ± 0.90 (5.5–9.2)	5.6 ± 0.53 (4.6–6.4)	5.9 ± 0.28 (5.6–6.2)	4.7 ± 0.44 (4.2–5.0)	5.9–6.0	5.3	7.2 ± 0.15 (7.0–7.3)	6.5 ± 0.27 (6.2–6.7)
Interorbital distance (IOD)	2.6 ± 0.24 (2.2–3.1)	2.1 ± 0.13 (1.9–2.3)	2.4 ± 0.13 (2.2–2.5)	1.9 ± 0.17 (1.7–2.0)	2.3–2.4	1.8	2.5 ± 0.24 (2.3–2.8)	2.2 ± 0.10 (2.1–2.3)
Internarial distance (IND)	2.0 ± 0.17 (1.7–2.3)	1.7 ± 0.19 (1.4–2.0)	1.8 ± 0.22 (1.5–2.0)	1.5 ± 0.15 (1.4–1.7)	1.6	1.4	1.9 ± 0.10 (1.8–2.0)	1.9 ± 0.12 (1.8–2.0)
Upper eyelid width (EW)	1.6 ± 0.21 (1.3–1.9)	1.4 ± 0.18 (1.2–1.7)	1.6 ± 0.13 (1.4–1.7)	1.3 ± 0.06 (1.3–1.4)	1.4–1.5	1.3	1.9 ± 0.14 (1.7–2.0)	1.7 ± 0.15 (1.6–1.9)
Eye diameter (ED)	2.4 ± 0.27 (2.0–2.8)	2.0 ± 0.18 (1.8–2.3)	2.1 ± 0.10 (2.0–2.2)	1.7 ± 0.15 (1.6–1.9)	2.1	1.9	2.3 ± 0.08 (2.2–2.4)	2.2 ± 0.06 (2.1–2.2)
Eye-nostril distance (EN)	1.8 ± 0.26 (1.4–2.3)	1.3 ± 0.13 (1.1–1.5)	1.7 ± 0.08 (1.6–1.8)	1.4 ± 0.06 (1.3–1.4)	1.8–1.9	1.6	2.1 ± 0.05 (2.0–2.1)	1.8 ± 0.10 (1.7–1.9)
Tympanum diameter (TD)	1.0 ± 0.19 (0.8–1.4)	0.9 ± 0.14 (0.6–1.1)	-	-	-	-	-	-
Femur length (FL)	9.0 ± 0.55 (7.4–10.0)	7.2 ± 0.59 (6.3–8.0)	7.7 ± 0.42 (7.3–8.3)	6.3 ± 0.44 (5.8–6.60)	8.1–8.3	7.0	9.5 ± 0.66 (9.1–10.5)	8.9 ± 0.36 (8.6–9.30
Tibia length (TL)	9.5 ± 0.58 (8.4–10.4)	7.5 ± 0.39 (6.7–8.2)	8.3 ± 0.35 (7.9–8.7)	6.7 ± 0.35 (6.4–7.1)	8.6–9.0	7.6	10.5 ± 0.49 (10.2–11.2)	9.4 ± 0.56 (8.9–10.0)
Foot length (FoL)	8.9 ± 0.85 (7.6–10.2)	6.9 ± 0.55 (5.7–7.5)	7.7 ± 0.45 (7.3–8.3)	5.9 ± 0.25 (5.7–6.2)	7.2–7.5	6.1	9.1 ± 0.50 (8.7–9.8)	8.1 ± 0.38 (7.8–8.5)
Hand length (HaL)	5.6 ± 0.62 (4.4–6.9)	4.2 ± 0.27 (3.7–4.7)	4.7 ± 0.39 (4.2–5.1)	3.6 ± 0.31 (3.3–3.9)	4.3–4.4	3.7	5.7 ± 0.34 (5.3–6.1)	5.2 ± 0.12 (5.1–5.3)
HW/SVL	34.5–38.6	34.6–37.1	32.8–35.7	32.7–33.7	32.2–33.7	32.0	35.6–37.0	33.2–34.4
HL/SVL	26.6–36.9	28.6–33.9	30.0–32.7	28.6–29.7	29.2–29.3	31.4	31.3–33.7	31.2–32.7
HL/HW	76.4–98.6	80.7–96.8	87.9–92.4	87.5–89.1	87.0–90.8	98.1	88.0–90.9	91.8–98.5
EN/HL	22.4–30.3	20.8–26.1	27.4–30.4	28.0–31.0	30.0–32.2	30.2	28.6–28.8	27.3–28.4
ED/HL	30.1–40.0	32.2–40.0	33.9–36.1	34.7–38.1	35.0–35.6	35.8	31.4–32.9	32.8–33.9
EW/IOD	51.7–73.1	60.0–80.0	63.6–70.8	65.0–76.5	60.9–62.5	72.2	71.4–82.6	76.2–82.6
EN/ED	66.7–92.0	57.9–79.0	80.0–85.0	73.7–82.4	85.7–90.5	84.2	87.5–91.3	81.0–86.4
TD/ED	34.8–51.9	33.3–47.8	-	-	-	-	-	-
FL/SVL	35.9–46.0	36.3–46.6	38.3–42.7	38.5–40.0	39.5–41.1	41.4	40.4–45.1	43.2–43.9
TL/SVL	37.6–48.6	38.5–46.6	40.3–49.1	39.6–43.5	42.0–44.6	45.0	44.7–49.0	44.7–47.2
FoL/SVL	35.2–43.8	35.4–42.9	37.8–42.7	35.8–38.8	35.1–37.1	36.1	38.6–42.1	39.1–40.1
HaL/SVL	22.6–27.5	21.2–25.8	21.9–26.3	22.4–23.1	21.0–21.8	21.9	24.0–26.2	25.0–25.6

Values are given as mean ± SD (range). Female body mass includes eggs.

**Fig 9:**
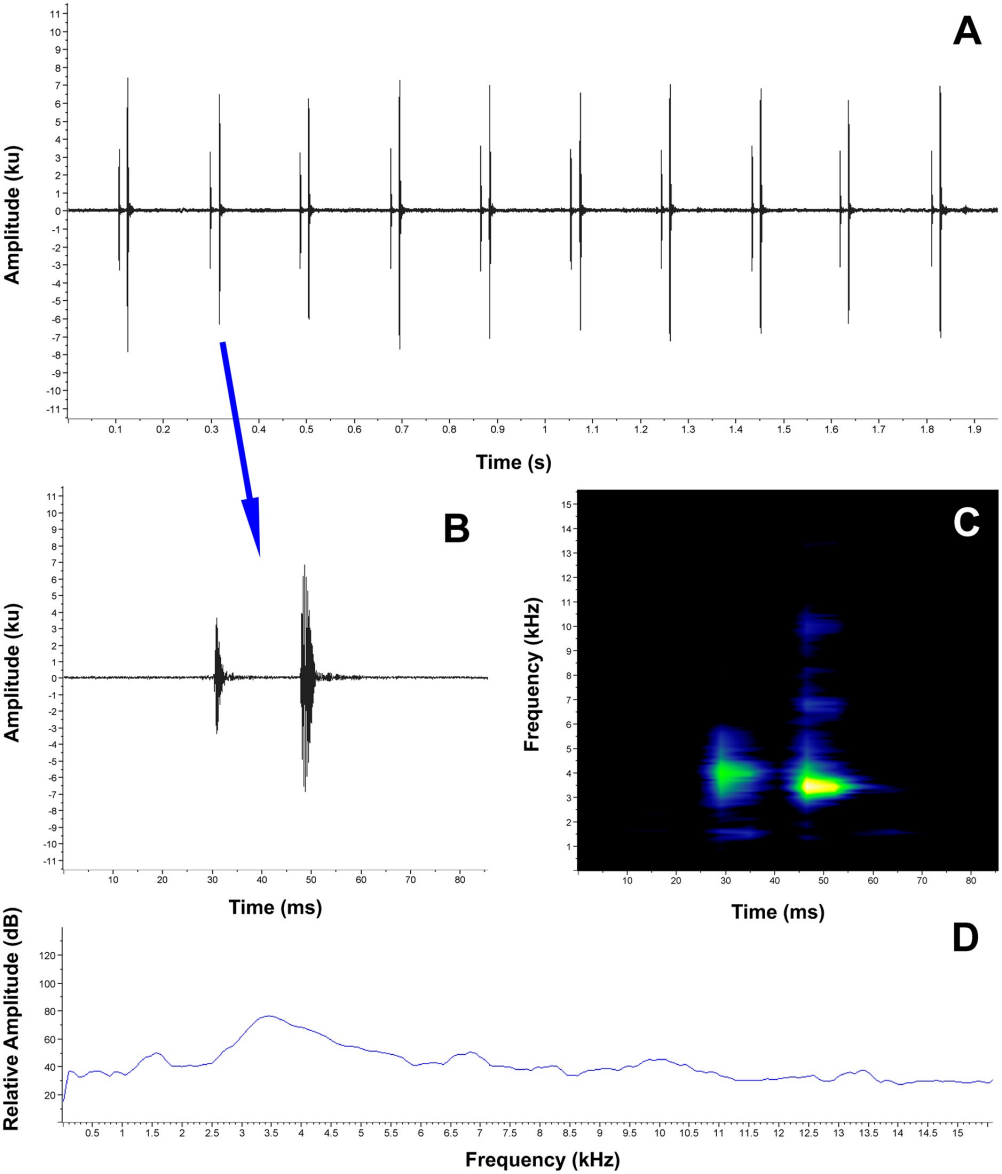
Advertisement call of *Pristimantis vidua* (FUTPL-A 239). **A**. Oscillogram of a 10 calls section of the call series; **B**. Oscillogram of a single call; **C**. Spectrogram of a single call; **D**. Power spectrum of a single call.

**Advertisement call**. The advertisement call of one male from Abra de Zamora was recorded in 2017 and of two males from Reserva Madrigal del Podocarpus in 2018. Descriptive statistics of the acoustic variables are provided in Table 2 (the detailed information of each of the separate recordings is presented in the S4 Table). *Pristimantis vidua* has an advertisement call characterized by a call series composed by clicking calls repeated for long periods of time, similar to the call of *P*. *tiktik* (Fig 9). The calls are composed only by two short, single-pulsed notes (tonal sounds). Because the males can call continuously for long periods of time, the call series duration is unknown. The calls are characterized by a mean duration of 0.022 s (SD = 0.004), a mean inter-call interval of 0.196 s (SD = 0.028) and a mean call rate of 291.3 calls/min (SD = 36.817). The mean dominant frequency of the call was 3302.2 Hz (SD = 124.359), with a mean 90% bandwidth of 3109.6–3605.6 Hz. The fundamental frequency is not recognizable, but 2 to 4 harmonics are usually visible.

**Table 2:** Quantitative description of the advertisement calls (mean ± SD, range and *n*) of the *Pristimantis tiktik*, *P*. *vidua*, *P*. *samaniegoi* sp. nov., *P*. *balionotus*, and *P*. *versicolor*.

	*Pristimantis tiktik* (11)	*Pristimantis vidua* (3)	*P*. *samaniegoi* sp. nov. (5)	*Pristimantis balionotus* (3)	*Pristimantis versicolor* (4)
Notes per call	1	2	usually 2	1–8 (usually 1 or 2)	1–5 (usually 1)
Call duration (s)	0.013 ± 0.004 (0.007–0.024) *n* = 330	0.022 ± 0.004 (0.013–0.029) *n* = 117	0.027 ± 0.008 (0.017–0.040) *n* = 116	0.069 ± 0.005 (0.061–0.078) *n* = 31	0.084 ± 0.006 (0.070–0.099) *n* = 109
Inter-call interval (s)	0.287 ± 0.029 (0.225–0.354) *n* = 319	0.196 ± 0.028 (0.157–0.259) *n* = 114	0.272 ± 0.027 (0.234–0.362) *n* = 112	6.075 ± 2.371 (3.499–15.677) *n* = 67	1.804 ± 0.315 (1.364–3.337) *n* = 136
Call rate (calls/min)	200.6 ± 18.905 (173.6–237.0) *n* = 11	291.3 ± 36.817 (249.0–316.4) *n* = 3	185.9 ± 25.398 (160.8–212.6) *n* = 5	10.0 ± 1.610 (8.6–11.8) *n* = 3	30.3 ± 3.138 (27.0–33.8) *n* = 4
Short notes duration (s)	N/A	0.004 ± 0.001 (0.002–0.006) *n* = 97	0.004 ± 0.001 (0.002–0.006) *n* = 116	N/A	N/A
Long notes duration (s)	N/A	0.009 ± 0.004 (0.003–0.017) *n* = 97	0.007 ± 0.001 (0.003–0.010) *n* = 116	N/A	N/A
Inter-note interval (s)	N/A	0.009 ± 0.005 (0.002–0.017) *n* = 97	0.015 ± 0.007 (0.006–0.028) *n* = 115	0.215 ± 0.011 (0.196–0.249) *n* = 39	0.208 ± 0.027 (0.168–0.325) *n* = 94
Dominant frequency (Hz)	3190.8 ± 99.873 (3014.6–3359.2) *n* = 330	3302.2 ± 124.359 (3100.8–3531.5) *n* = 273	3140.3 ± 144.886 (2842.4–3359.2) *n* = 294	2431.7 ± 59.371 (2325.6–2584.0) *n* = 69	1753.6 ± 80.323 (1636.5–2067.2) *n* = 139
Frequency 5% (Hz)	3059.5 ± 112.207 (2842.4–3273.1) *n* = 330	3109.6 ± 93.588 (2842.4–3273.1) *n* = 273	2994.4 ± 156.623 (2670.1–3186.9) *n* = 294	2331.8 ± 39.947 (2239.5–2411.7) *n* = 69	1542.3 ± 60.568 (1378.1–1636.5) *n* = 138
Frequency 95% (Hz)	3390.5 ± 86.571 (3186.9–3617.6) *n* = 330	3605.6 ± 170.730 (3273.1–3876.0) *n* = 273	3496.2 ± 284.2 (3186.9–4306.6) *n* = 294	2542.8 ± 45.772 (2497.9–2670.1) *n* = 69	3269.2 ± 283.50 (2842.4–4478.9) *n* = 138

The call of *P*. *tiktik* is presented with comparative purposes as it is very similar to the *P*. *vidua* and *P*. *samaniegoi* sp. nov. calls. In the case of *P*. *balionotus* and *P*. *versicolor* the call duration is presented for the one-noted calls and the Inter-note interval is for the multi-noted calls. The number of recorded individuals is given in brackets after species name.

Compared to the call of *P*. *tiktik*, which is single-noted, the call of *P*.*vidua* is composed of two notes: a first short one, and a second, slightly longer. Also, its call is very similar to the advertisement call of *P*. *samaniegoi* sp. nov. (the differences between the two calls are presented further below, in the description of the *P*. *samaniegoi* sp. nov. call).

**Distribution**. *Pristimantis vidua* presence is confirmed (with DNA samples) from Parque Nacional Podocarpus (Cajanuma entrance), Reserva Madrigal del Podocarpus, Abra de Zamora and Bosque Protector Washapamba, in Loja and Zamora Chinchipe provinces (Fig 6). The presence of this species in the Azuay province [9] is yet to be confirmed. We consider this unlikely, since we do not have any valid records of this species in any inventoried similar ecosystems north of Saraguro (close to the interprovincial border). The species was encountered at an altitudinal range between 2750 and 3000 m a.s.l. in montane cloud forest and subpáramo ecosystems.

**Natural history**. Lynch (1979) described the species based on 15 females, and hypothesized that maybe this species might be “all-female”, or at the least that *P*. *vidua* exhibits a “preponderance of females” (female-biased sex ratio). We found males although most of the encountered individuals proved to be females. We thus agree that the species has a female-biased sex ratio; alternatively, the detectability might be different between the sexes, as usually the females were found perched on leaves (at 20 cm to 1.5 m above the ground), while the males were encountered somewhat hidden in the vegetation or calling from inside the moss at the base of the shrubs. This is a common species. Calling males were encountered all year round but more frequently on rainy nights.

**Conservation status**. *Pristimantis vidua* is currently categorized as Endangered [49] based on criteria B1 ab(iii). Even if this is a relatively abundant species at all localities, and its habitat does not face any major threats (it is encountered in one national protected area and two private and/or community protected areas), we suggest maintaining this category. This is due the fact that the species Extent of occurrence (EOO) and Area of occupancy (AOO) are estimated to be less than 200 km^2^, it is currently known only from 4 localities, and the existing populations are severely fragmented.

**Remarks**. Lynch [9] provides a detailed description of this species, including a brief description of the cranial osteology. Our diagnosis concurs with most of the morphological features described by the author (characters given in parentheses), but we also added descriptions of the characters that are more evident in life. Some of the differences that we observed are: skin on dorsum finely tuberculated (in contrast to the finely shagreened dorsum described by Lynch); dentigerous processes of vomers bearing 3 to 9 teeth (2 to 4 teeth); small, inconspicuous, ulnar tubercles present (no ulnar tubercles or fold); heel with 2 or 3 larger and several smaller tubercles (no heel tubercles); inner tarsal tubercles coalesced into a long tarsal fold (tarsus bearing ill-defined tubercle along inner edge). The diagnosis provided herein is based on 12 specimens from the original description (KU 120082–89, 120090–92, 141994), 3 adult females (MUTPL 295, 312, 805) and 6 adult males (MUTPL 296, 495–499, 501), collected from the type locality and 25 specimens collected from the other localities.

*Pristimantis vidua* is part of the *P*. *orestes* subgroup, and is most closely related to an undescribed species from Parque Nacional Podocarpus (Lagunas del Compadre). However, its exact position in the subgroup remains non-resolved (Fig 7). The genetic distance between *P*. *vidua* and its closest relatives from the *P*. *orestes* subgroup ranges between 4.6–9.2% (S3 Table).

***Pristimantis samaniegoi* sp. nov**. Székely, Eguiguren, Ordóñez-Delgado, Armijos-Ojeda, and Székely

**urn:lsid:zoobank.org:act:0B9E2517-1BE9-4361-BB7D-D5B34120C248**

(Figs 10–13)

**Fig 10:**
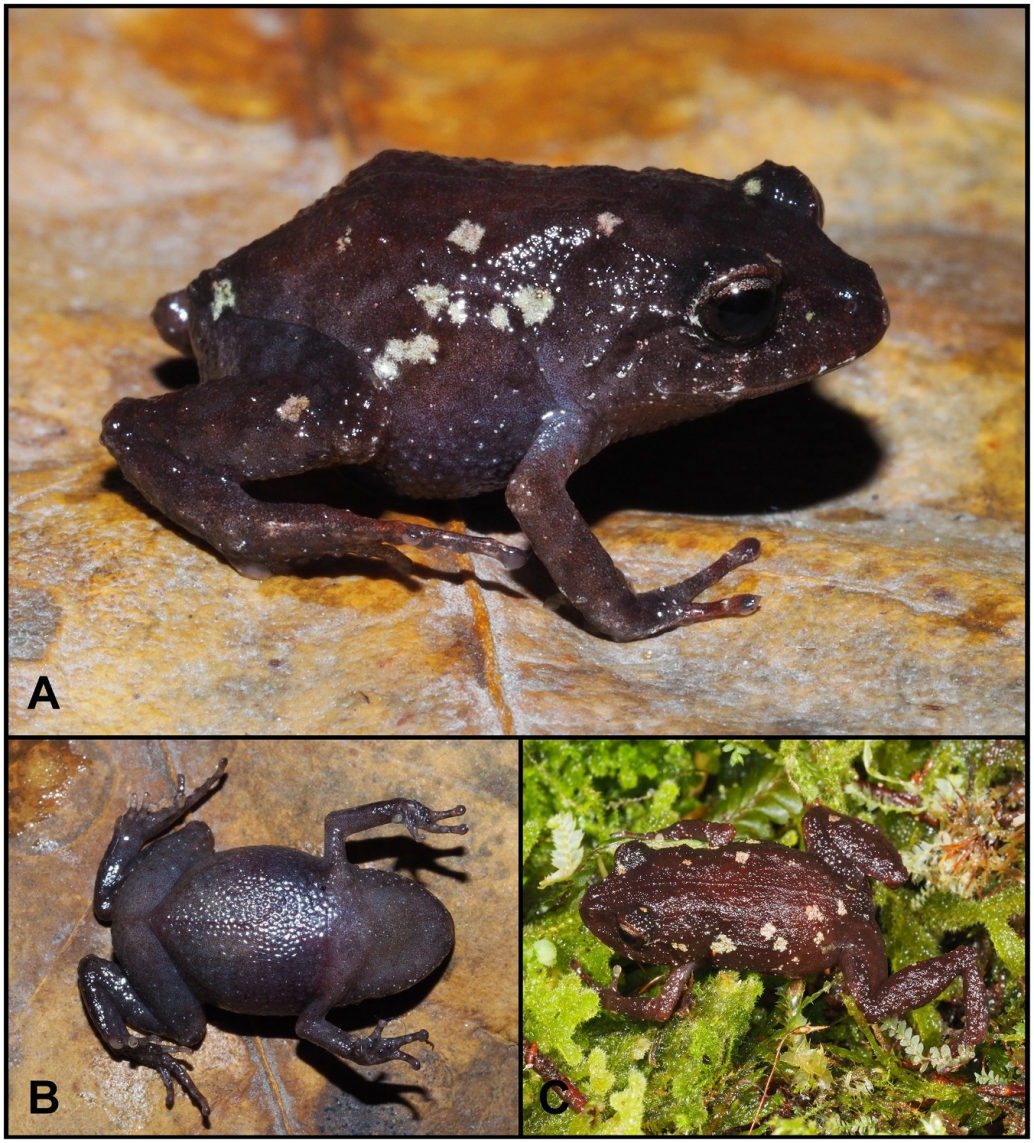
Holotype of *Pristimantis samaniegoi* sp. nov. (MUTPL 357, adult female), SVL 20.1 mm, in life. **A**. Lateral view; **B**. Ventral view; **C**. Dorsal view.

**Fig 11:**
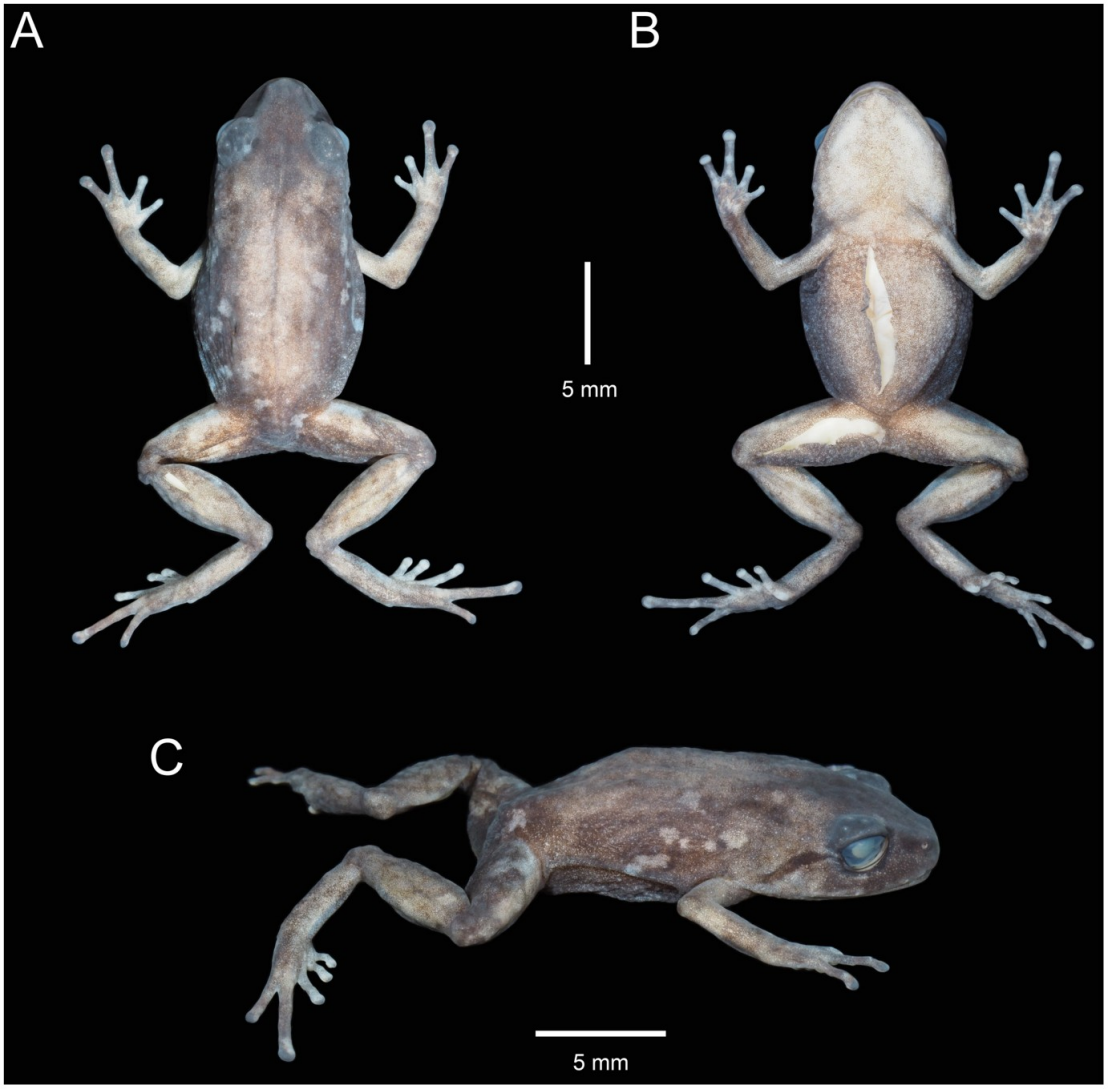
Holotype of *Pristimantis samaniegoi* sp. nov. (MUTPL 357, adult female) in preservative. **A**. Dorsal view; **B**. Ventral view; **C**. Lateral view.

**Fig 12:**
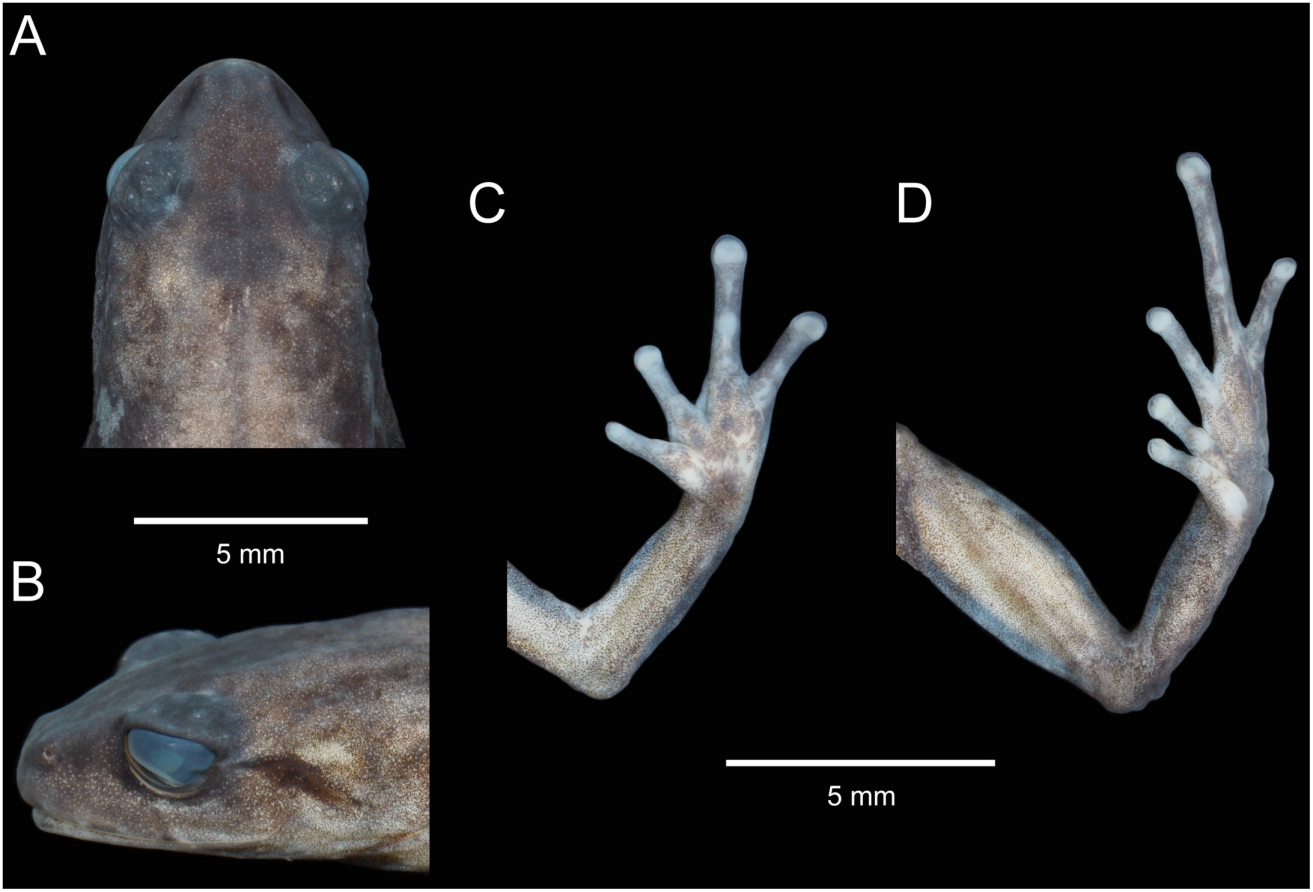
Holotype of *Pristimantis samaniegoi* sp. nov. (MUTPL 357, adult female) in preservative. **A**. Dorsal view of head; **B**. Profile view of head; **C**. Palmar view of hand: **D**. Plantar view of foot.

**Fig 13:**
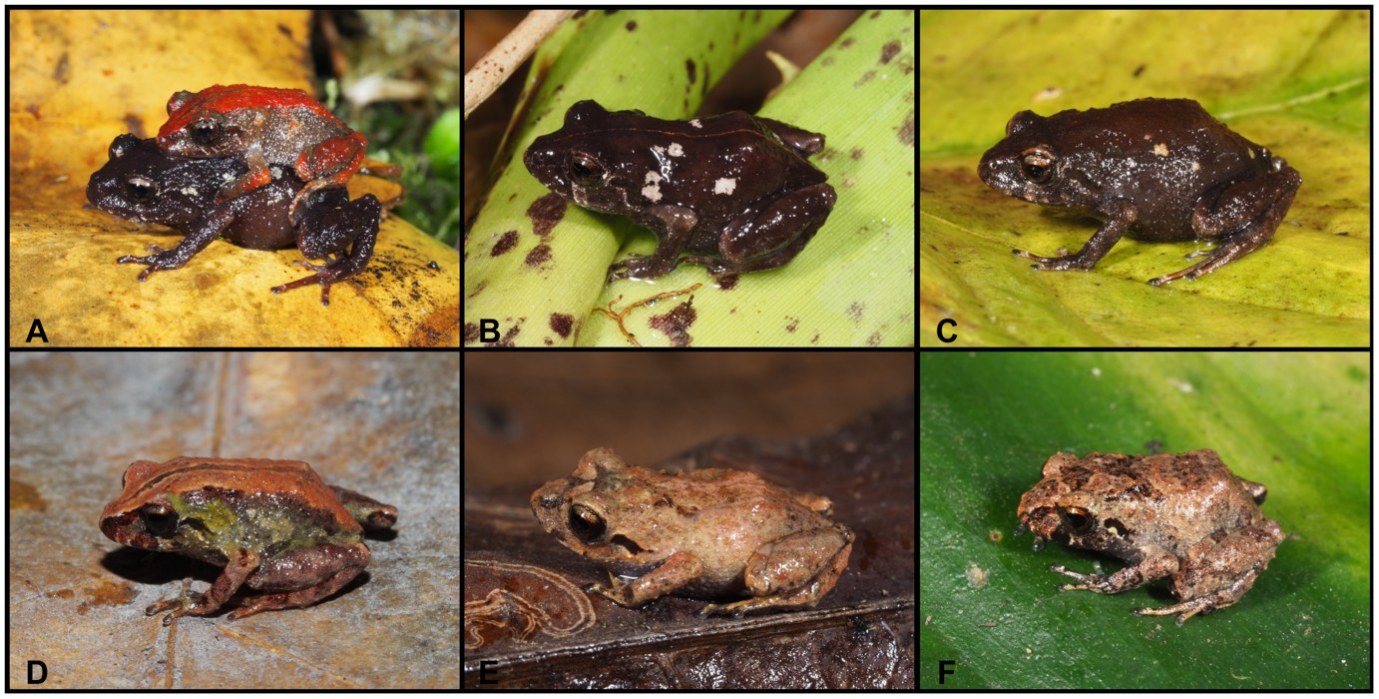
Color variation of *Pristimantis samaniegoi* sp. nov. in life. **A**. amplexus: female, holotype (MUTPL 357) and male, paratype (MUTPL 356); **B–C** females, paratypes: **B**. MUTPL 676, **C**. MUTPL 777; **D–E** males, paratypes: **D**. MUTPL 358, **E**. MUTPL 674; **F**. juvenile, paratype: MUTPL 660. Abra de Zamora (B, C, E, F), Parque Nacional Podocarpus—Cajanuma (A, D).

**Common English name**. Samaniego’s Rain Frog

**Common Spanish name**. Cutín de Samaniego

**Etymology**. The specific epithet is a noun in the genitive case and is a patronym for Dr. Gustavo Samaniego Rodríguez. The name is given as a tribute to his important contribution to the conservation of biodiversity in southern Ecuador. Through a generous donation of an important part of his estate, he contributed to the creation of the administrative and interpretation center in the Cajanuma sector of the Parque Nacional Podocarpus, which is one of the most important Ecuadorian sites of endemism and biodiversity. He was a university professor for over 40 years, and currently still maintains the "Reserva El Cristal" agro-ecological farm as an example of sustainable land management.

**Holotype**. MUTPL 357 (Figs 10–13A), field no. SC 170, an adult female from Ecuador, Loja Province, Cajanuma entrance to the Parque Nacional Podocarpus, on Los Miradores trail (4.1151° S, 79.1651° W; datum WGS84), 3258 m above sea level, collected by Paul Székely and Diana Székely on 28 June 2018.

**Paratypes (3 females, 3 males and 1 juvenile)**. Two specimens collected syntopically with the holotype: MUTPL 356 (SC 169) and MUTPL 358 (SC 171), both adult males (Fig 13A and 13D). Five specimens from Ecuador, Loja Province, Abra de Zamora: MUTPL 365 (SC 178), an adult female collected by Paul Székely (3.9912° S, 79.1460° W; datum WGS84), 2817 m, on 19 July 2018; MUTPL 660 (SC 253), a juvenile (Fig 13F) collected by Stalin Gómez, Alejandro Ochoa, Michelle Angamarca and Paul Székely (3.9836° S, 79.1307° W; datum WGS84), 2569 m, on 27 April 2019; MUTPL 674 (SC 993), an adult male (Fig 13E) collected by Paul Székely, Stalin Gómez, Alejandro Ochoa and Michelle Angamarca (3.9928° S, 79.1469° W; datum WGS84), 2865 m, on 17 May 2019; MUTPL 676 (SC 936), an adult female (Fig 13B) collected by Paul Székely, Stalin Gómez and Alejandro Ochoa (3.9869° S, 79.1443° W; datum WGS84), 2827 m, on 24 May 2019; MUTPL 777 (SC 1030) an adult female (Fig 13C) collected by Stalin Gómez and Alejandro Ochoa (3.9965° S, 79.1469° W; datum WGS84), 2891 m, on 22 September 2019.

**Diagnosis**. We assign this species to *Pristimantis* based on phylogenetic evidence (Fig 7) and on the general morphological similarity to other members of the genus. *Pristimantis samaniegoi* is a small species, distinguished by the following combination of traits: (1) skin on dorsum finely tuberculated (in life the skin tuberculated texture is more evident); skin on venter coarsely areolate; thoracic and discoidal folds evident (trait more visible in preservative); dorsolateral folds absent; low middorsal fold present; (2) tympanic annulus and tympanic membrane absent; supratympanic fold present; (3) snout short, rounded in dorsal view and in profile; canthus rostralis weakly concave in dorsal view, rounded in profile; (4) upper eyelid bearing several small tubercles (trait more visible in life), about 67% IOD in females and 70% IOD in males; cranial crests absent; (5) dentigerous processes of vomers prominent, oblique, triangular or oval in outline, separated medially by distance almost equal with the width of processes; each processes bearing 4 to 6 teeth; (6) males with large subgular vocal sac and vocal slits; nuptial pads absent; (7) Finger I shorter than Finger II; discs on fingers just slightly expanded, rounded; circumferential grooves present; fingers lacking lateral fringes; (8) subarticular tubercles prominent, round and subconical in section; supernumerary palmar tubercles rounded, large, just slightly smaller than subarticular tubercles; palmar tubercle completely divided into a larger (inner) and a smaller (outer) tubercles; thenar tubercle oval, smaller than the inner palmar tubercle; (9) ulnar tubercles absent or very small, inconspicuous (trait more visible in life); (10) heel with several small tubercles (trait more visible in life); outer edge of tarsus with a row of small tubercles (trait more visible in life); inner tarsal tubercles coalesced into a long tarsal fold; (11) inner metatarsal tubercle broadly ovoid, about 3x elongated and subconical outer metatarsal tubercle; supernumerary plantar tubercles ovoid, large, just slightly smaller than subarticular tubercles; (12) Toe V slightly longer than Toe III; discs on toes just slightly expanded, rounded, about same size as those on fingers; circumferential grooves present; toes lacking lateral fringes; webbing basal; (13) evident sexual dimorphism: in life, the females with dorsum, flanks, dorsal surfaces of hindlimbs and arms dark brown with various white, irregular spots and dark gray venter; males dorsum light brown, reddish brown or red, without white spots and much lighter venter, pinkish white or light gray; no markings in axilla, groin or on concealed limb surfaces; iris whitish gray with a reddish bronze, broad, median horizontal streak and with fine black reticulations; (14) SVL 17.1–20.7 mm in adult females (19.3 ± 1.58 SD, *n* = 4) and 14.7–16.9 mm in adult males (16.0 ± 1.17 SD, *n* = 3).

**Comparisons with similar species**. *Pristimantis samaniegoi* is morphologically similar to its closest relatives, the species from the *P*. *orestes* group, but it can be easily distinguished from all the resembling species. From the 14 species that are currently included in this group, only three species lack tympanic annulus and tympanic membrane: *P*. *samaniegoi*, *P*. *matildae* sp. nov. and *P*. *colodactylus*. However, *P*. *colodactylus* and *P*. *matildae* sp. nov. belong to the *P*. *colodactylus* subgroup of species (Fig 7), specialized to live inside bromeliads, that have a very distinct habitus, with longer snouts/heads, longer legs, head as wide or wider than the body and usually very short fingers and toes (Table 1). Additionally, *P*. *samaniegoi* can be easily distinguished from *P*. *matildae* sp. nov. and *P*. *colodactylus* as follows (characters of *P*. *samaniegoi* in parenthesis): discoidal fold absent (thoracic and discoidal folds evident), supratympanic fold absent (present), upper eyelid bearing one prominent tubercle (upper eyelid bearing only several small tubercles), dentigerous processes of vomers concealed in buccal mucosa (dentigerous processes of vomers prominent), Toe V much longer than Toe III (Toe V slightly longer than Toe III), and males lacking vocal sac and slits (males with large subgular vocal sac and vocal slits).

*Pristimantis samaniegoi* can be easily distinguished from other closely related species, which are somewhat similar, as follows (characters of *P*. *samaniegoi* in parenthesis): *P*. *quintanai*, *P*. *saturninoi*, *P*. *simonbolivari*, *P*. *bambu* and *P*. *mazar* have spots—black, white or yellow—in axilla, groin, concealed limb surfaces, or on venter (spots absent) and *P*. *tiktik* has tuberculated dorsum (finely tuberculated) and the venter, axillae and groins of the females is white with black reticulum (venter dark gray, without reticulum). From the sympatric species, *P*. *andinognomus* is significantly smaller, with females on average 16 mm, males 13 mm (larger, with females on average 19 mm, males 16 mm), has inguinal spots (no inguinal spots), and Toe V is much longer than Toe III (Toe V is slightly longer than Toe III), *P*. *cajanuma* has inguinal spots (spots absent) and *P*. *vidua* is larger, with females on average 22 mm, males 17 mm (smaller, with females on average 19 mm, males 16 mm) and has dorsolateral folds (dorsolateral folds absent). And finally, none of the other species have the typical dark coloration with white spots of the females of *P*. *samaniegoi*.

**Description of the holotype**. Adult female (MUTPL 357; Figs 10–13A), head narrower than body, wider than long, head length 92% of head width, head width 33% of SVL; head length 30% of SVL; snout short (snout to eye distance 13% of SVL), rounded in dorsal view and in profile (Fig 12A and 12B); canthus rostralis weakly concave in dorsal view, rounded in profile; loreal region flat; eye diameter notably greater than eye-nostril distance; nostrils not protuberant; lips not flared; cranial crests absent; upper eyelid bearing several small tubercles (two slightly larger than the others), width of upper eyelid 64% of IOD; tympanic annulus and tympanic membrane absent (Fig 12B); evident supratympanic fold present; one larger, rounded postrictal tubercle present; choanae large, oval, not concealed by palatal shelf of maxillary arch; dentigerous processes of vomers prominent, slightly larger than the choanae, oblique, situated posterior and median to choanae, triangular in outline, separated medially by distance almost equal to the width of processes, each processes bearing 4 to 5 teeth; tongue 1.5x as long as wide, slightly notched posteriorly, posterior half not adherent to floor of mouth.

Skin on dorsum finely tuberculated; thin, low middorsal fold starting at tip of snout and ending at cloaca; dorsolateral folds absent; skin on throat, chest, belly, and ventral surfaces of thighs coarsely areolate; thoracic and discoidal folds evident (trait more visible in preservative; Figs 10B and 11B); ornamentation in cloacal region absent.

Ulnar tubercles absent; outer palmar tubercle prominent, completely divided into a larger (inner) and a smaller (outer) tubercles; thenar tubercle oval, smaller than the inner palmar tubercle; subarticular tubercles prominent, round and subconical in section; supernumerary palmar tubercles rounded, large, just slightly smaller than subarticular tubercles; fingers lacking lateral fringes; relative length of fingers I < II < IV < III; discs on fingers just slightly expanded, rounded; all fingers bearing pads well defined by circumferential grooves (Fig 12C).

Hindlimbs short; tibia length 40% of SVL; foot length 38% of SVL; heel with several small tubercles; outer edge of tarsus with a row of small tubercles (trait more visible in life); inner edge of tarsus bearing a long fold; inner metatarsal tubercle broadly ovoid, about 3x elongated and subconical outer metatarsal tubercle; subarticular tubercles prominent, ovoid and subconical in section; plantar supernumerary tubercles ovoid, large, just slightly smaller than subarticular tubercles; toes lacking lateral fringes; webbing basal; discs on toes just slightly expanded, rounded, about same size as those on fingers; toes with ventral pads well defined by circumferential grooves (Fig 12D); relative length of toes I < II < III < V < IV; Toe V slightly longer than Toe III (tip of Toe III barely reaching the penultimate subarticular tubercle on Toe IV, tip of Toe V not reaching the proximal edge of distal subarticular tubercle on Toe IV).

**Coloration of holotype**. In life (Fig 10): dorsum, flanks, dorsal surfaces of hindlimbs and arms dark brown with various yellowish white, irregular spots; venter and throat gray, without spots; iris whitish gray with a dark bronze, broad, median horizontal streak and with fine black reticulations.

In preservative (Fig 11): the coloration of dorsum, flanks and dorsal surfaces of hindlimbs became dark gray and the spots light gray; dorsal surfaces of arms, venter and throat became light gray.

**Measurements of holotype (in mm)**: SVL 20.1; HW 6.6; HL 6.1; IOD 2.5; IND 2.0; EW 1.6; ED 2.2; EN 1.8; snout to eye distance 2.6; FL 7.7; TL 8.1; FoL 7.6; HaL 4.8; Finger I length 2.2.

**Body mass of holotype**: 0.80 g.

**Variation**. Morphometric variation is shown in Table 1. This species displays an evident sexual dimorphism. The females are larger, and are dark brown with various white, irregular spots and have a dark gray venter (Fig 13A, 13B and 13C). One female (MUTPL 676, Fig 13B) had a whitish orange low middorsal fold. The same individual had very distinctive white lines on the center of the venter and throat, ventral surfaces of hindlimbs and arms. The males have lighter colors of the dorsum that varies from uniform light brown (MUTPL 674, Fig 13E), to reddish brown dorsum and light green and gray flanks (MUTPL 358, Fig 13D). The male MUTPL 356 (Fig 13A) had a bright red coloration of the dorsum and dorsal surfaces of hindlimbs and arms and gray flanks. The venter coloration of the males varied from pinkish white to light gray. None of the males had the characteristic white spots of the females. In all specimens, the supratympanic fold was black.

**Fig 14:**
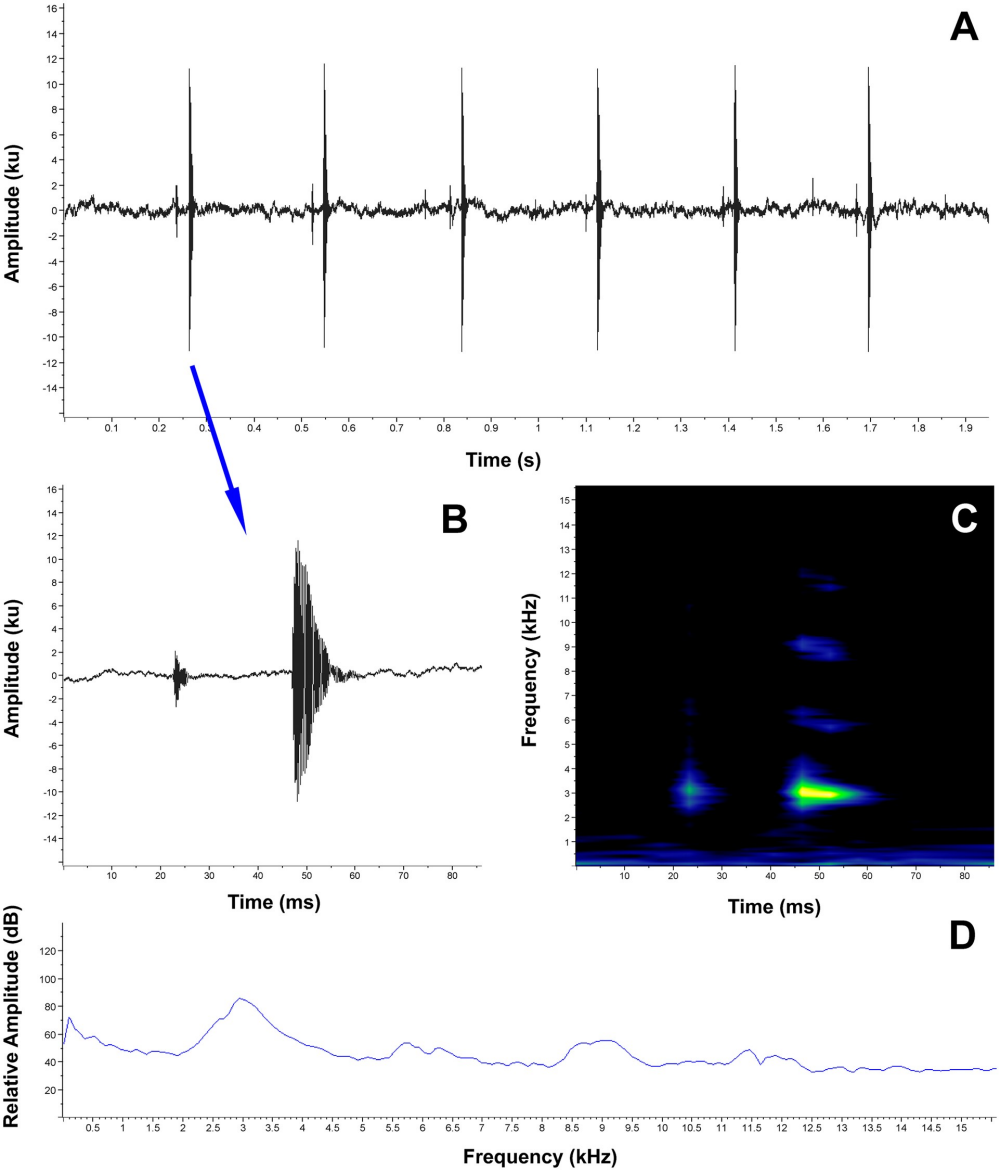
Advertisement call of *Pristimantis samaniegoi* sp. nov. (FUTPL-A 242). **A**. Oscillogram of a 6 calls section of the call series; **B**. Oscillogram of a single call; **C**. Spectrogram of a single call; **D**. Power spectrum of a single call.

**Advertisement call**. The advertisement call of one male from Abra de Zamora was recorded in in 2017 and of four males from Cajanuma in 2018. Descriptive statistics of the acoustic variables are provided in Table 2 (the detailed information of each of the separate recordings is presented in the S4 Table). *Pristimantis samaniegoi* has an advertisement call characterized by a call series composed by clicking calls repeated for long periods of time, similar to the calls of *P*. *tiktik* and *P*. *vidua* (Fig 14). The calls are composed by two short, single-pulsed notes (tonal sounds). The male MUTPL 356 (FUTPL-A-241) had the calls composed by only one note but it is possible that this is actually a recording artifact. Because the males can call continuously for long periods of time, the call series duration is unknown. The calls are characterized by a mean duration of 0.027 s (SD = 0.008), a mean inter-call interval of 0.272 s (SD = 0.027) and a mean call rate of 185.9 calls/min (SD = 25.398). The mean dominant frequency of the call was 3140.3 Hz (SD = 144.886), with a mean 90% bandwidth of 2994.4–3496.2 Hz. The fundamental frequency is not recognizable but 3 to 4 harmonics are visible.

The advertisement call of *P*. *samaniegoi* is most similar to that of *P*. *tiktik*, with similar inter-call intervals, call rates, dominant frequency and 90% bandwidth frequency (Table 2). However, the main difference is that *P*. *samaniegoi* has double-noted calls as opposed to the single-noted calls of *P*. *tiktik* (Fig 14). The call of *P*. *samaniegoi* is also similar to that of *P*. *vidua*, but has a longer call duration, longer inter-call interval, a lower call rate, longer inter-note interval and, most importantly, lower dominant and 90% bandwidth frequency (Table 2).

**Distribution**. *Pristimantis samaniegoi* is known only from the Cajanuma entrance to the Parque Nacional Podocarpus and about 13 km in the north, in Abra de Zamora (Fig 6). We did not encounter this species in any similar habitats located in the vicinity of the type locality, despite fieldwork being carried out regularly since 2016. The species was encountered at an altitudinal range between 2560 and 3300 m a.s.l., in evergreen upper montane forest (Fig 2C), subpáramo (Fig 2B) and shrub páramo ecosystems.

**Natural history**. This is a common species. All the specimens were encountered during the night, close to the ground (usually at 5–20 cm above the ground), at the base of the grassy vegetation, on the moss bed under the shrubs, or on shrub leaves. Individuals are hard to find, as they are well hidden at the base of the shrubs or inside the moss bed or grassy vegetation. However, the male’s characteristic advertisement call can be commonly heard. Calling males were encountered year round, more frequently on rainy nights. All the females were encountered in the immediate proximity of the calling males.

**Conservation status**. *Pristimantis samaniegoi* is known only from two close localities, from an estimated area of less than 50 km^2^. Nonetheless, we recommend that this species be categorized as Near Threatened following the IUCN criteria [47], due the fact that it is a relatively abundant species and its habitat currently does not face any major threats (most of the population is situated within a nationally protected area—Parque Nacional Podocarpus).

**Remarks**. *Pristimantis samaniegoi* is part of the *P*. *simonbolivari* subgroup, but its kinship to the other members of the subgroup remains non-resolved (Fig 7). In our phylogram *P*. *samaniegoi* is most closely related to *P*. *quintanai* and an undescribed species, but with only weak BI support. The genetic distance between *P*. *samaniegoi* and its closest relatives from the *P*. *simonbolivari* subgroup ranges between 4.8–6.0% (*P*. *tiktik*) and 6.7–7.3% (*P*. *bambu*) (S3 Table).

***Pristimantis colodactylus*** (Lynch, 1979)

(Fig 15)

**Fig 15:**
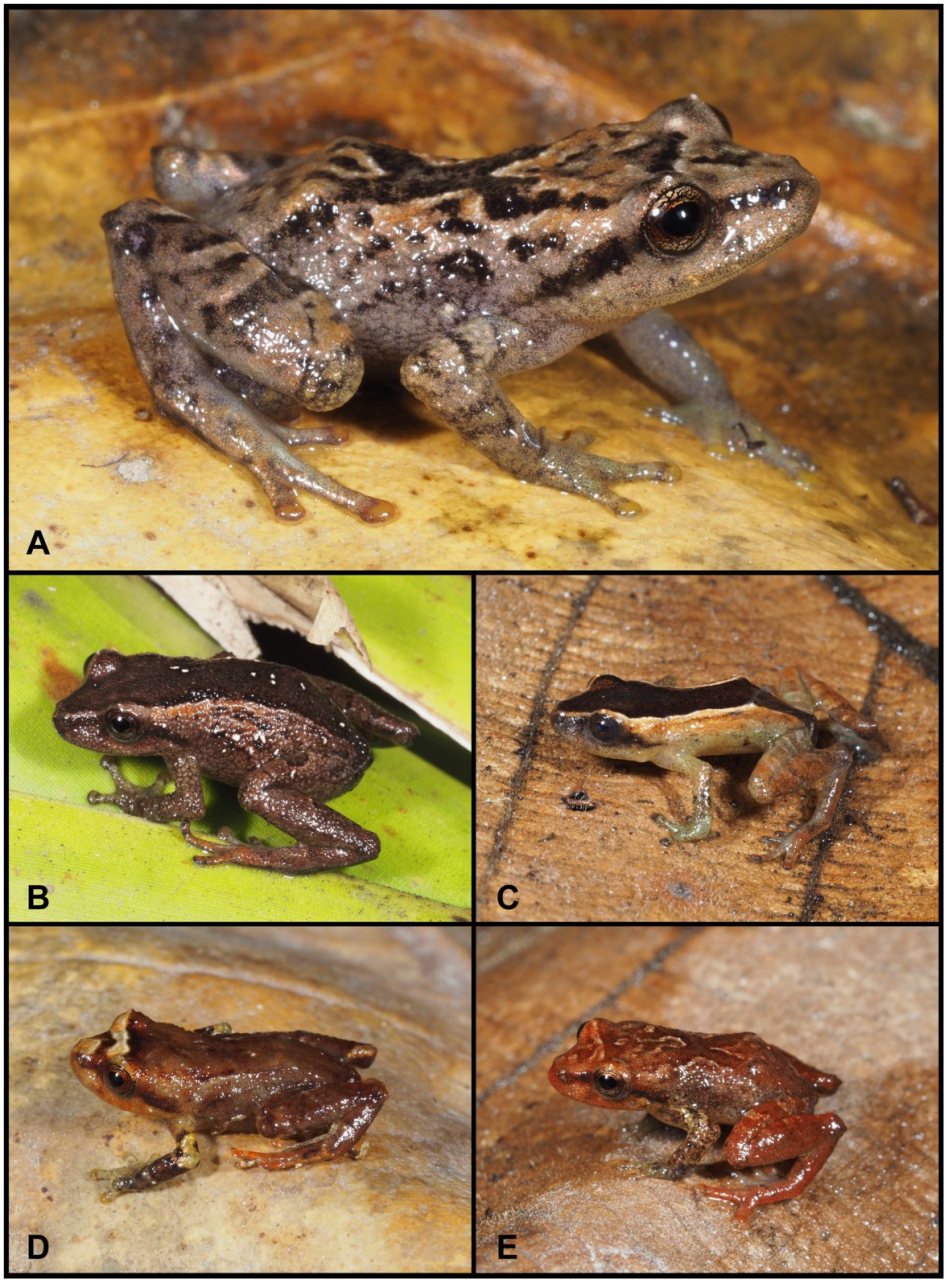
Color variation of *Pristimantis colodactylus* in life. **A–B** females: **A**. MUTPL 311, **B**. MUTPL 388; **C**. male: MUTPL 675; **D–E** juveniles: **D**. MZUA 2491, **E**. MUTPL 732. All specimens are from Abra de Zamora.

*Eleutherodactylus colodactylus* Lynch, 1979

*Eleutherodactylus* (*Eleutherodactylus*) *colodactylus*: Lynch, 1996; Lynch and Duellman, 1997

*Pristimantis colodactylus*: Heinicke, Duellman, and Hedges, 2007

*Pristimantis* (*Pristimantis*) *colodactylus*: Hedges, Duellman, and Heinicke, 2008

**Common English name**. Stubby Fingered Rain Frog (This is a newly proposed name as it is more appropriate. The previous one “Piura Robber Frog” makes no sense, as the species is known only from its type locality, Abra de Zamora, Ecuador).

**Common Spanish name**. Cutín de dedos cortos

**Etymology**. The specific name is derived from the Greek *kolos*, meaning “stubby” and the Greek *daktylo*, meaning “toes”, referring to the short fingers and toes of this species [25].

**Holotype**. KU 142151, an adult female from 13.5 km E Loja, at the crest (Abra de Zamora) on the frontier between Loja and Zamora-Chinchipe provinces, Ecuador, 2800 m, obtained on 22 July 1971 by William E. Duellman.

**Paratypes**. KU 142152–59 topotypes; KU 142160–61, 14 km E Loja, Provincia Zamora-Chinchipe, 2770 m; KU 142162–64, 15 km E Loja, Provincia Zamora-Chinchipe, 2710 m.

**Diagnosis**. *Pristimantis colodactylus* is a small species distinguished by the following combination of traits: (1) skin on dorsum areolate, almost tuberculate (in life the skin areolate texture is more evident); skin on venter areolate; discoidal fold and dorsolateral folds absent; (2) tympanic membrane and tympanic annulus absent; supratympanic fold absent; (3) snout rounded in dorsal view and in profile; canthus rostralis straight in dorsal view, rounded in profile; (4) upper eyelid bearing one prominent tubercle and several small tubercles, about 62% IOD in females and 72% IOD in males; cranial crests absent; (5) dentigerous processes of vomers concealed in buccal mucosa; each processes bearing 2 to 6 teeth; (6) males lacking vocal sac and slits; (7) fingers very short and stocky; Finger I shorter than Finger II; discs on fingers slightly expanded, rounded; circumferential grooves present; fingers bearing lateral fringes; (8) subarticular tubercles prominent; supernumerary palmar tubercles numerous, continuing onto fingers; palmar tubercle completely divided into a larger (inner) and a smaller (outer) tubercles; thenar tubercle oval, the same size or slightly smaller than the inner palmar tubercle; (9) ulnar tubercles absent; (10) heel bearing one moderate-sized conical tubercle and several small tubercles (trait more visible in life); inner and outer edges of tarsus lacking tubercles or folds; (11) inner metatarsal tubercle broadly ovoid, about 4 to 5x rounded outer metatarsal tubercle; supernumerary plantar tubercles numerous, continuing onto toes; (12) toes very short and stocky; Toe V much longer than Toe III; discs on toes slightly expanded, rounded, about same size as those on fingers; circumferential grooves present; toes bearing prominent lateral fringes; webbing basal; (13) in life, dorsum various shades of brown with or without pale dorsolateral stripes, interorbital bars, or dark X-shaped markings on the back; venter cream with dark flecks; no markings in axilla, groin or on concealed limb surfaces; iris light bronze with a reddish median horizontal streak and fine black reticulations; (14) SVL 16.5–25.8 mm in adult females [9] and 14.0–20.7 mm in adult males [9].

**Variation**. Morphometric variation is shown in Table 1. The five encountered specimens had variable coloration (Fig 15). Both female MUTPL 311 (Fig 15A) and juvenile MUTPL 732 (Fig 15E) had a dark X-shaped marking on their back (equivalent to what Lynch described as “hour-glass” [9]), but the female had a general grayish coloration with light and dark brown spots and stripes, while the juvenile had a general reddish brown coloration. Both female MUTPL 388 (Fig 15B) and male MUTPL 675 (Fig 15C) had a dark brown middorsal band, but the female had darker flanks and members than the male. The coloration of the male MUTPL 675 is very similar to that of the holotype KU 142151, where the dark brown middorsal band is bordered by light whitish dorsolateral stripes that continue onto the snout (Fig 15C). The coloration of juvenile MZUA 2491 (Fig 15D) is very similar with that of the male specimen KU 165219 (Fig 3D in [9] and Fig 139 in [25]), with dark brown dorsum and whitish yellow interorbital bar and snout.

**Advertisement call**. Unknown.

**Distribution**. For now, *P*. *colodactylus* presence is confirmed (with DNA samples) from only a small area in its type locality, Abra de Zamora (Fig 6). The majority of specimens were encountered outside of Parque Nacional Podocarpus borders. We did not encounter this species in the Cajanuma sector of the Parque Nacional Podocarpus (about 13 km to the south), which has a similar species composition to Abra de Zamora. The presence of this species in Azuay and Morona Santiago provinces [9] is yet to be confirmed. We consider this highly unlikely, and that it is more plausible that these are different, currently undescribed species. Also, it is almost certain that the animals from Peru [9, 25] are different, undescribed species, taking into consideration the great distance from the type locality. Even Lynch noted some morphological differences between the specimens from the type locality and the ones from central Ecuador and Peru (see Table 1 in [9]). The species was encountered at an altitudinal range between 2800 and 2820 m a.s.l. in a subpáramo ecosystem (Fig 2B).

**Natural history**. This species shares the same microhabitat (bromeliads) with *P*. *matildae* sp. nov., which dominates in term of abundance. It is a rare species. Between 2016 and 2020, in Abra de Zamora, we encountered only five specimens of *P*. *colodactylus*. This is a bromeliad specialist that most likely spends its entire life inside or nearby these plants. The short fingers of the species of the *P*. *colodactylus* subgroup (Fig 7) are probably adaptations for the life inside bromeliads, and possibly help to increase the adhesivity of the hands and feet [29]. Four specimens were found inside bromeliads (both terrestrial and arboreal) during the day or night. One specimen (MUTPL 675) was found outside, on the leaf of a bromeliad (probably foraging), and when it detected our presence rapidly retreated backwards to the water inside the plant. No calling males were heard.

**Conservation status**. *Pristimantis colodactylus* is currently categorized as Least Concern [50]. However, this assessment was done by considering the records from the provinces of Azuay and Morona Santiago and from Peru. Based on the available (revised) information we consider *P*. *colodactylus* to be Critically Endangered following the B1ab(iii,iv)+2ab(iii,iv) IUCN criteria [47] because: (1) its Extent of occurrence (EOO) and Area of occupancy (AOO) are estimated to be less than 10 km^2^; (2) it is known from only one location; and (3) its habitat could be severely affected in the near future by deforestation, as it is situated mostly outside a protected area and in the vicinity of a densely populated area.

**Remarks**. Lynch [9] provides a detailed description of this species, including a brief description of the cranial osteology. Our diagnosis concurs with the morphological features described by the author (one small difference would be the coloration of the iris that we found to be light bronze and not reddish bronze), and we add descriptions of the characters that are more evident in life. The diagnosis provided herein is based on 14 specimens from the original description (KU 142151–64), two adult females (MUTPL 311, 388) one adult male (MUTPL 675) and two juveniles (MUTPL 732, MZUA 2491) collected from the type locality.

*Pristimantis colodactylus* is part of the *P*. *colodactylus* subgroup, and as such it is closely related to *P*. *matildae* sp. nov., *P*. *muranunka* and an additional undescribed species from Reserva Tapichalaca (Fig 7). The genetic distance between *P*. *colodactylus* and its closest relatives from the subgroup ranges between 5.4–7.5% (S3 Table).

***Pristimantis matildae* sp. nov**. Székely, Eguiguren, Ordóñez-Delgado, Armijos-Ojeda, and Székely

**urn:lsid:zoobank.org:act:E2E120CF-C257-4A95-9052-879CA1387C89**

(Figs 16–19)

**Fig 16:**
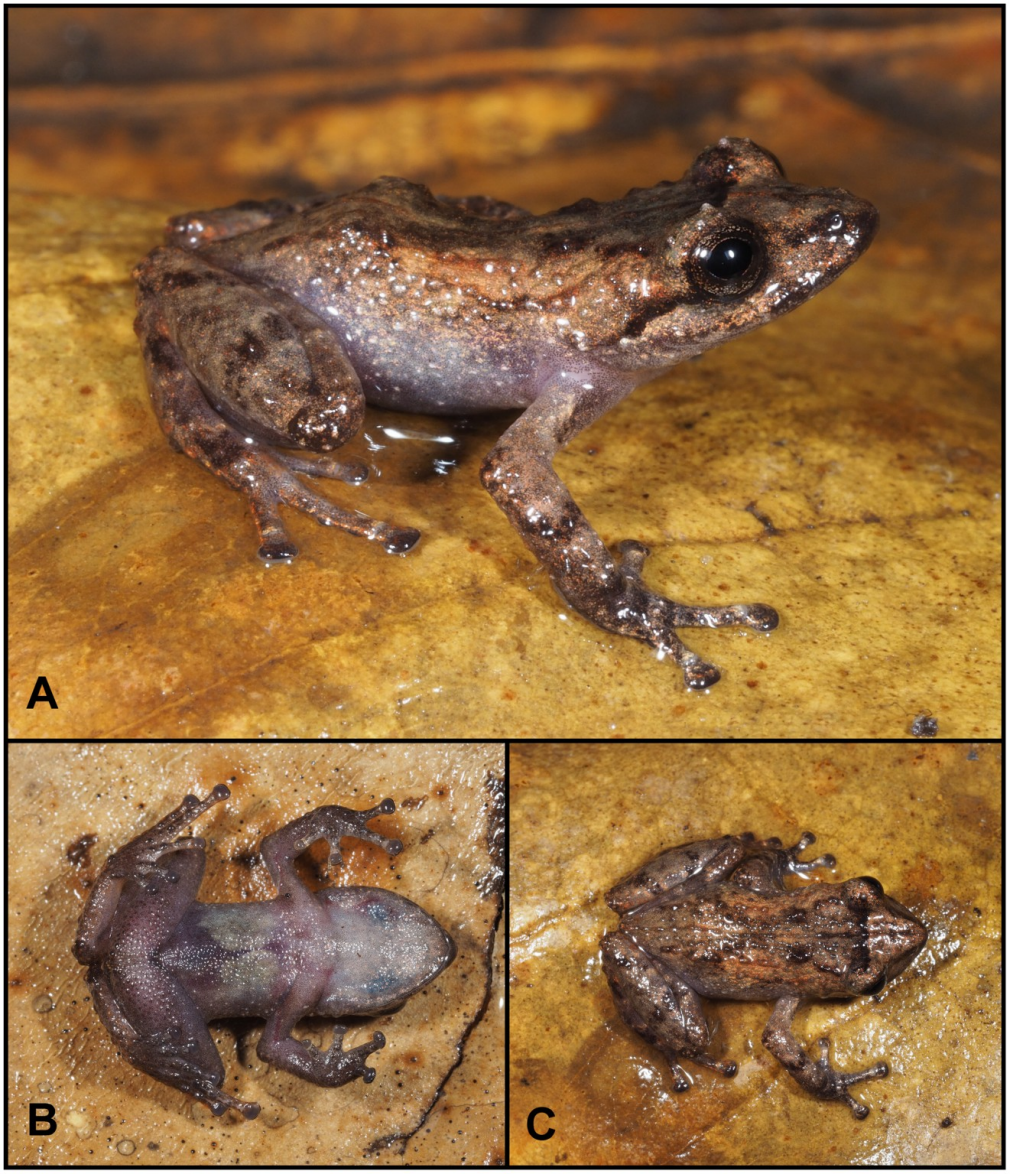
Holotype of *Pristimantis matildae* sp. nov. (MUTPL 731, adult male), SVL 20.2 mm, in life. **A**. Lateral view; **B**. Ventral view; **C**. Dorsal view.

**Fig 17:**
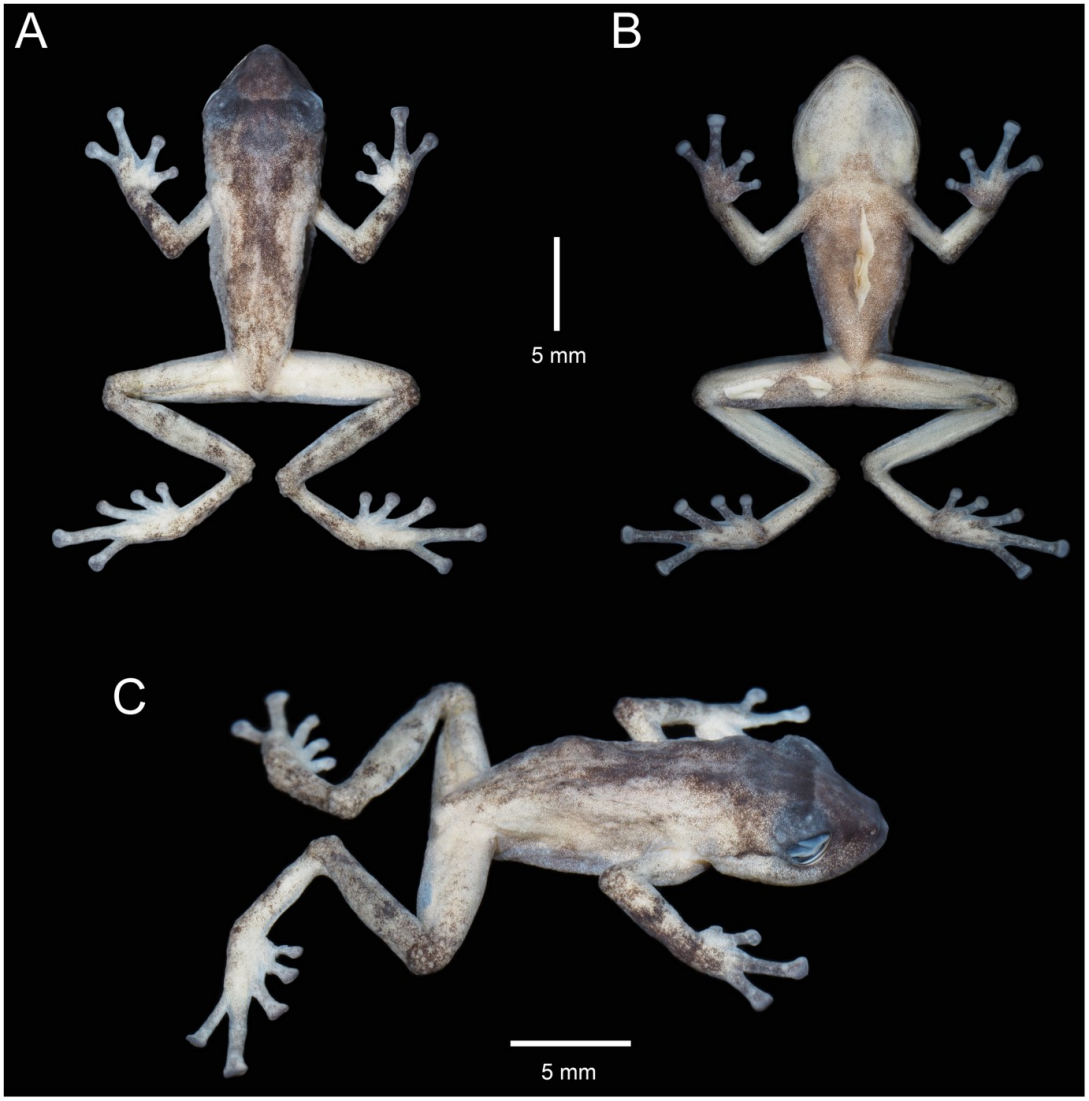
Holotype of *Pristimantis matildae* sp. nov. (MUTPL 731, adult male) in preservative. **A**. Dorsal view; **B**. Ventral view; **C**. Lateral view.

**Fig 18:**
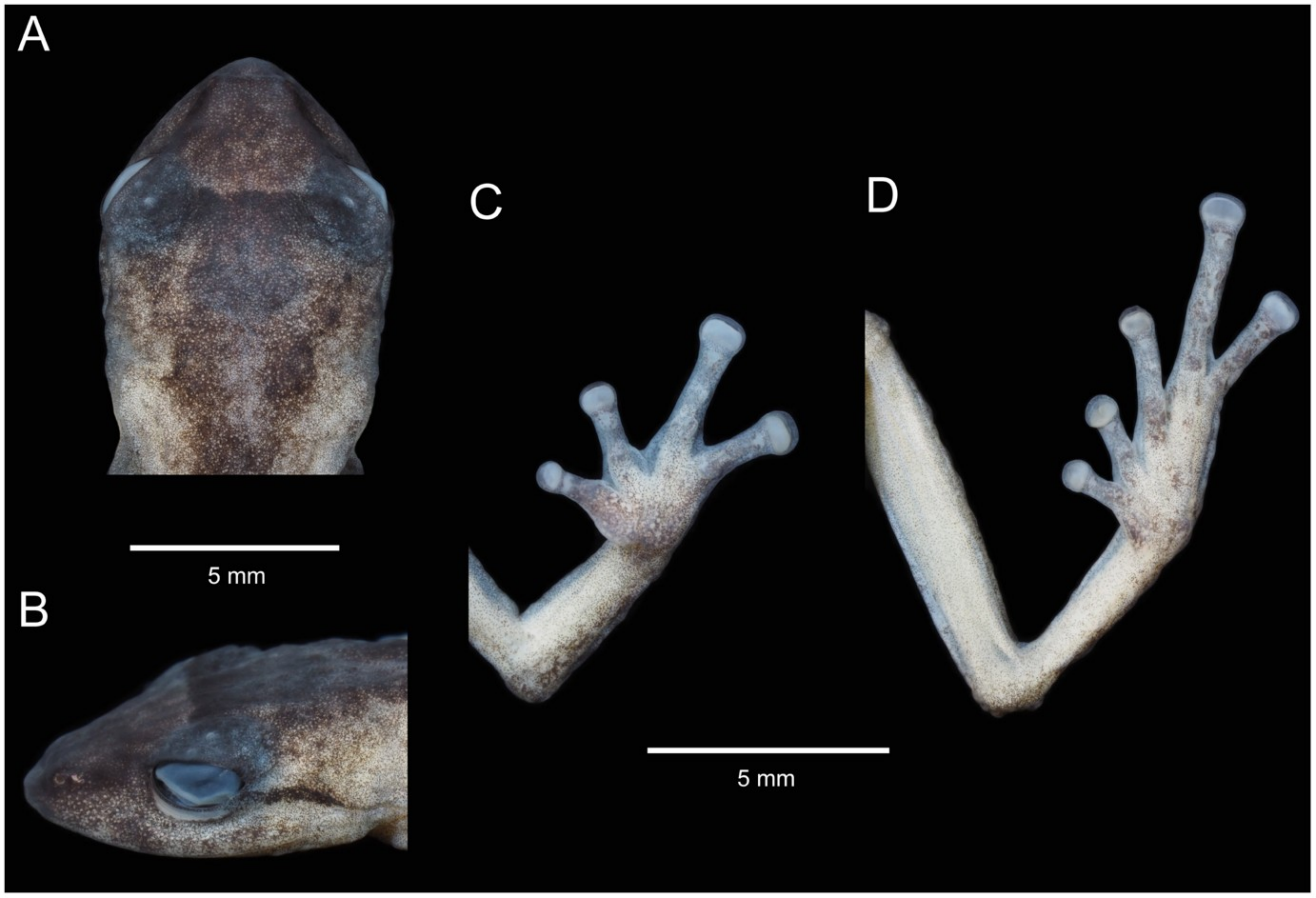
Holotype of *Pristimantis matildae* sp. nov. (MUTPL 731, adult male) in preservative. **A**. Dorsal view of head; **B**. Profile view of head; **C**. Palmar view of hand: **D**. Plantar view of foot.

**Fig 19:**
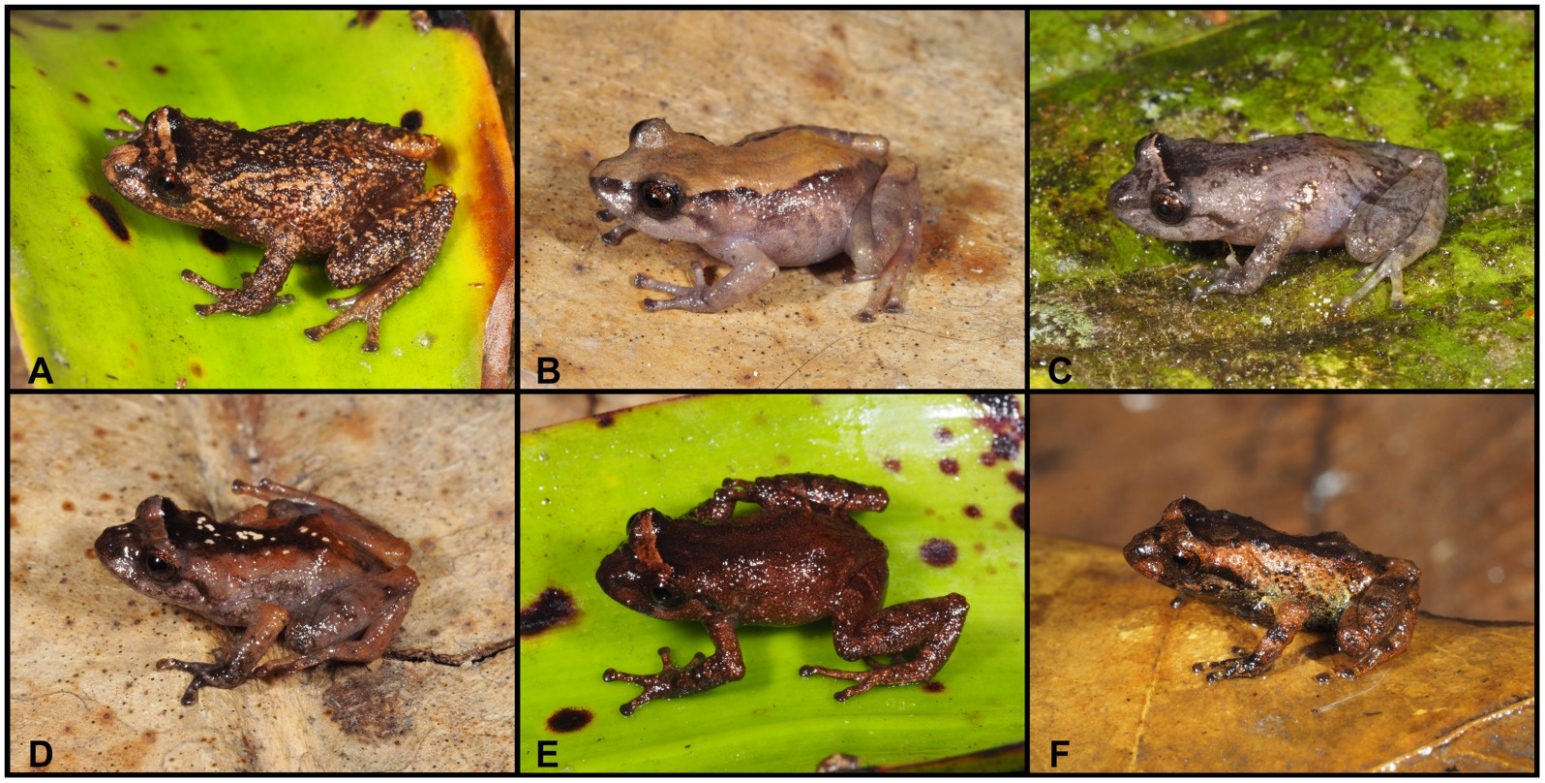
Color variation of *Pristimantis matildae* sp. nov. in life. **A–C** females, paratypes: **A**. MUTPL 361, **B**. MUTPL 394, **C**. MUTPL 813; **D–E** males, paratypes: **D**. MUTPL 362, **E**. MUTPL 360; **F**. juvenile, paratype: MUTPL 733. Abra de Zamora (B, F), Parque Nacional Podocarpus—Cajanuma (A, C, D, E).

**Common English name**. Matilde’s Rain Frog

**Common Spanish name**. Cutín de Matilde

**Etymology**. The specific epithet is a noun in the genitive case and is a patronym for Matilde Hidalgo Navarro (1889–1974). She was the first woman to obtain a medical degree in the country, and the first woman in Ecuador (and Latin America) to exercise the right to vote in a national election. A tireless fighter for women’s rights, she laid important foundations for the development of women in academia and science, nationally and in South America. In Ecuador, there are currently many women involved in biological sciences and with this species, in addition to being a tribute to Matilde, we honor their tireless work for the conservation of biological diversity.

**Holotype**. MUTPL 731 (Figs 16–18), field no. SC 958, an adult male from Ecuador, Loja Province, Abra de Zamora (3.9910° S, 79.1458° W; datum WGS84), 2815 m above sea level, collected by Paul Székely, Stalin Gómez and Alejandro Ochoa on 6 August 2019.

**Paratypes (4 females, 2 males and 1 juvenile)**. Three specimens collected in the type locality: MUTPL 733 (SC 960), a juvenile (Fig 19F) collected syntopically with the holotype; MUTPL 394 (SC 152), an adult female (Fig 19B) collected by Diana Székely, Veronica Urgiles, Anna Savage and Paul Székely (3.9910° S, 79.1455° W; datum WGS84), 2815 m, on 31 May 2018; MUTPL 366 (SC 179), an adult female collected by Paul Székely (3.9912° S, 79.1460° W; datum WGS84), 2817 m, on 19 July 2018; four specimens from Ecuador, Loja Province, Cajanuma sector, Parque Nacional Podocarpus: MUTPL 361 (SC 174) an adult female (Fig 19A) and MUTPL 360 (SC 173) and MUTPL 362 (SC 175) adult males (Fig 19D and 19E) collected by Diana Székely and Paul Székely (4.1141° S, 79.1627° W; datum WGS84), 3361 m, on 29 June 2018; MUTPL 813 (SC 1113), an adult female (Fig 19C) collected by Diana Székely and Paul Székely (4.1054° S, 79.1616° W; datum WGS84), 3347 m, on 23 November 2019.

**Diagnosis**. We assign this species to *Pristimantis* based on phylogenetic evidence (Fig 7) and on the general morphological similarity to other members of the genus. *Pristimantis matildae* is a small species distinguished by the following combination of traits: (1) skin on dorsum finely tuberculated (trait more visible in life); skin on venter areolate; discoidal fold and dorsolateral folds absent; low middorsal fold present; (2) tympanic annulus and tympanic membrane absent; supratympanic fold absent; (3) snout moderately long, subacuminate in dorsal view and subacuminate and slightly protruding in profile; canthus rostralis weakly concave in dorsal view, angular in profile; (4) upper eyelid bearing one prominent tubercle and several small tubercles, about 77% IOD in females and 79% IOD in males; cranial crests absent; (5) dentigerous processes of vomers concealed in buccal mucosa; each processes bearing 2 to 5 teeth; (6) males lacking vocal sac and slits; nuptial pads absent; (7) Finger I shorter than Finger II; discs on fingers slightly expanded, truncated; circumferential grooves present; fingers bearing lateral fringes; (8) subarticular tubercles prominent, round and rounded in section; supernumerary palmar tubercles prominent, rounded; palmar tubercle completely divided into a larger (inner) and a smaller (outer) tubercles; thenar tubercle ovoid, larger than the inner palmar tubercle; (9) ulnar tubercles absent; (10) heel with several small tubercles (trait more visible in life); outer edge of tarsus with a row of small, inconspicuous, tubercles (trait more visible in life); inner edge of tarsus bearing a long fold; (11) inner metatarsal tubercle broadly ovoid, about 3x elongated and rounded outer metatarsal tubercle; supernumerary plantar tubercles prominent, rounded; (12) Toe V much longer than Toe III; discs on toes slightly expanded, truncated, about same size as those on fingers; circumferential grooves present; toes bearing prominent lateral fringes; webbing basal; (13) in life, dorsum various shades of brown or gray with or without middorsal bands, interorbital bars, or dark X-shaped markings on the back; venter light gray or pinkish gray; no markings in axilla, groin or on concealed limb surfaces; iris bronze or reddish bronze with a dark median horizontal streak and fine black reticulations; (14) SVL 20.8–23.3 mm in adult females (22.5 ± 1.12 SD, *n* = 4) and 19.9–21.2 mm in adult males (20.4 ± 0.68 SD, *n* = 3).

**Fig 20:**
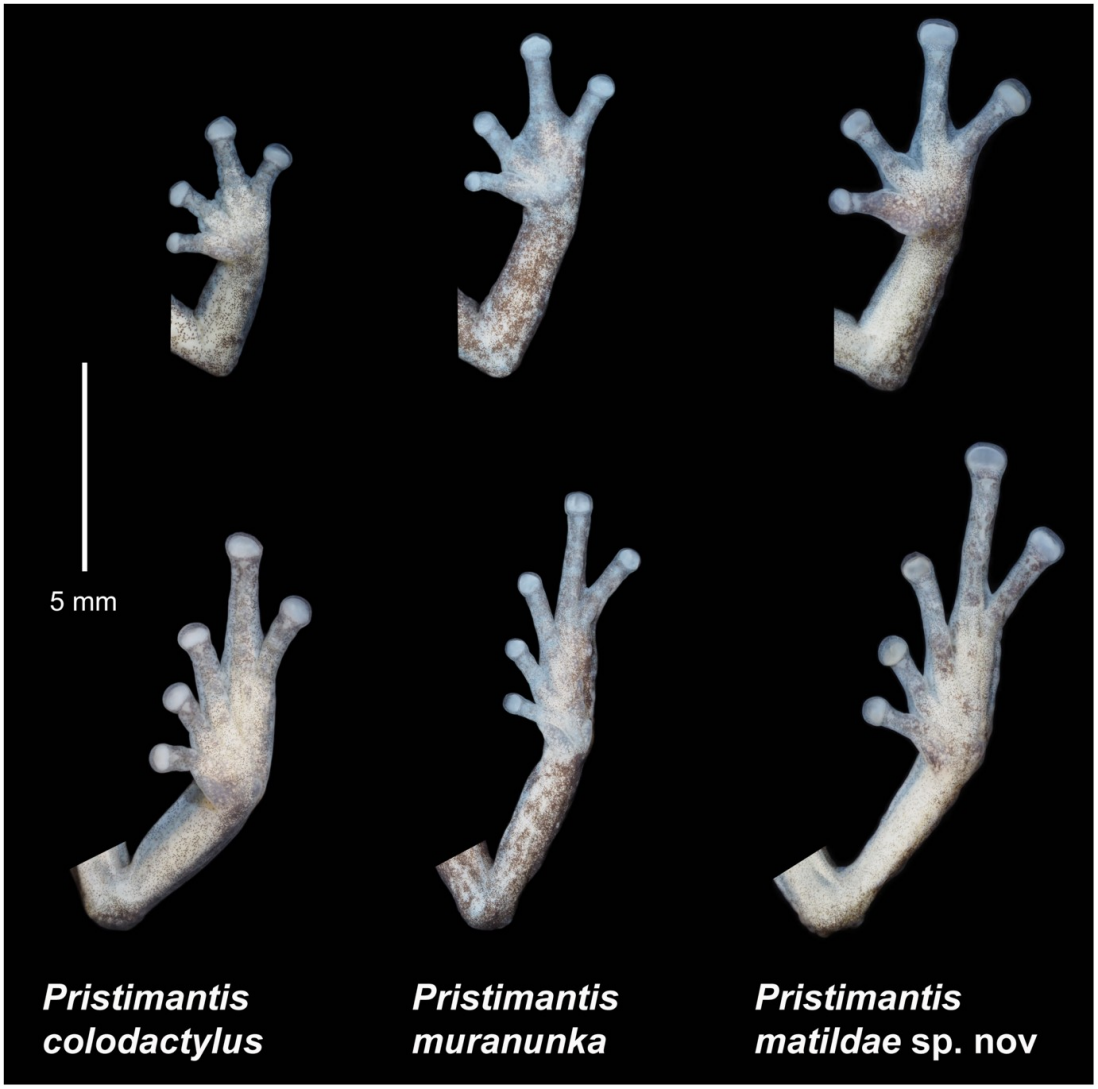
Relative size of the fingers and toes in *Pristimantis colodactylus* (MUTPL 388, SVL 20.5 mm), *P*. *muranunka* (MUTPL 652, SVL 20.1 mm) and *P*. *matildae* sp. nov. (Holotype, MUTPL 731, SVL 20.2 mm).

**Comparisons with similar species**. *Pristimantis matildae* is morphologically similar to its closest relatives, the species from the *P*. *orestes* group, and especially to the species from the *P*. *colodatylus* subgroup (Fig 7), *P*. *colodactylus* and *P*. *muranunka*. The difference between *P*. *matildae* and the species from the other subgroups were presented in the description of *P*. *samaniegoi*. *Pristimantis matildae* is easily distinguished from *P*. *muranunka* by the lack of tympanic annulus and tympanic membrane (tympanic annulus and tympanic membrane evident in *P*. *muranunka*). *Pristimantis matildae* best resembles *P*. *colodactylus*, but several morphological features can be used to distinguish between these two species. The most important feature is the relative size of the fingers and toes (Fig 20). From the three species currently included in this subgroup, *P*. *colodactylus* has the shortest fingers and toes (Hand length (length of Finger III) about 22% of SVL; Foot length (length Toe IV) about 36% of SVL), followed by *P*. *muranunka* (Hand length about 23% of SVL; Foot length about 38% of SVL), and finally, *P*. *matildae*, has the longest fingers and toes (Hand length about 25% of SVL; Foot length about 40% of SVL) (Fig 20, Table 1). Additionally, *P*. *matildae* can be distinguished from *P*. *colodactylus* as follows (characters of *P*. *matildae* in parenthesis): skin on dorsum and flanks areolate (finely tuberculate), low middorsal fold absent (present), heel with one moderate-sized conical tubercle and several small tubercles (without a larger tubercle, only with several small ones), ornamentation in cloacal region absent (cloacal region bordered ventrally by two large, oval, tubercles), thenar tubercle the same size or slightly smaller than the inner palmar tubercle (thenar tubercle larger than the inner palmar tubercle), inner edge of tarsus lacking tubercles (inner edge of tarsus bearing a long fold) and inner metatarsal tubercle about 4 to 5x outer metatarsal tubercle (inner metatarsal tubercle about 3x outer metatarsal tubercle). Also, the iris coloration in *P*. *matildae* is more reddish than in the case of *P*. *colodactylus*.

**Description of the holotype**. Adult male (MUTPL 731; Figs 16–18), head wider than body, just slightly wider than long, head length 99% of head width, head width 33% of SVL; head length 32% of SVL; snout moderately long (snout to eye distance 14% of SVL), subacuminate in dorsal view and subacuminate and slightly protruding in profile (Fig 18A and 18B); canthus rostralis weakly concave in dorsal view, angular in profile; loreal region slightly concave; eye diameter notably greater than eye-nostril distance; nostrils slightly protuberant, oriented posteriorly; lips not flared; cranial crests absent; upper eyelid bearing one prominent tubercle and several small tubercles, width of upper eyelid 77% of IOD; tympanic annulus and tympanic membrane absent (Fig 18B); supratympanic fold absent; one large, rounded postrictal tubercle present; choanae large, oval, not concealed by palatal shelf of maxillary arch; dentigerous processes of vomers concealed in buccal mucosa but each processes bearing two teeth; tongue 0.5x as long as wide, slightly notched posteriorly, posterior half not adherent to floor of mouth; vocal sac, vocal slits and nuptial pads absent.

Skin on dorsum and flanks finely tuberculated (trait more visible in life, Fig 16A and 16C); thin, low middorsal fold starting at tip of snout and ending at cloaca; dorsolateral folds absent; skin on chest, belly, and ventral surfaces of thighs areolate; thoracic and discoidal folds absent (Figs 16B and 17B); cloacal region bordered ventrally by two large, oval, tubercles.

Ulnar tubercles absent; outer palmar tubercle prominent, completely divided into a larger (inner) and a smaller (outer) tubercles; thenar tubercle ovoid, larger than the inner palmar tubercle; subarticular tubercles prominent, round and rounded in section; supernumerary palmar tubercles rounded, smaller than subarticular tubercles; fingers bearing lateral fringes; relative length of fingers I < II < IV < III; discs on fingers slightly expanded, truncated; all fingers bearing pads well defined by circumferential grooves (Fig 18C).

Hindlimbs long; tibia length 46% of SVL; foot length 39% of SVL; heel with several small tubercles; outer edge of tarsus with a row of small, inconspicuous, tubercles (trait more visible in life); inner edge of tarsus bearing a long fold; inner metatarsal tubercle broadly ovoid, about 3x elongated and rounded outer metatarsal tubercle; subarticular tubercles prominent, round and rounded in section; plantar supernumerary tubercles rounded, smaller than subarticular tubercles; toes bearing prominent lateral fringes; webbing basal; discs on toes slightly expanded, truncated, about same size as those on fingers; toes with ventral pads well defined by circumferential grooves (Fig 18D); relative length of toes I < II < III < V < IV; Toe V much longer than Toe III (tip of Toe III barely reaching the penultimate subarticular tubercle on Toe IV, tip of Toe V extends to the distal edge of distal subarticular tubercle on Toe IV).

**Coloration of holotype**. In life (Fig 16): dorsum, upper parts of flanks, dorsal surfaces of hindlimbs and arms grayish tan with dark markings; on the middle of the back a large dark X-shaped marking is visible; the head with dark brown interorbital bar, canthal and supratympanic stripes; lower parts of flanks, venter, throat and ventral surfaces of hindlimbs and arms pinkish gray; iris bronze with a dark median horizontal streak and fine black reticulations.

In preservative (Fig 17): the coloration of dorsum and dorsal surfaces of hindlimbs and arms dark gray, with the dark X-shaped marking evident; venter brownish gray; throat and ventral surfaces of hindlimbs and arms light gray.

**Measurements of holotype (in mm)**: SVL 20.2; HW 6.7; HL 6.6; IOD 2.2; IND 1.8; EW 1.7; ED 2.2; EN 1.8; snout to eye distance 2.9; FL 8.8; TL 9.3; FoL 7.9; HaL 5.1; Finger I length 2.4.

**Body mass of holotype**: 0.50 g.

**Variation**. Morphometric variation is shown in Table 1. Except for the female MUTPL 394 (Fig 19B), all specimens had a light interorbital bar flanked by a dark border. This female had the uniquely yellowish tan broad middorsal band, bordered by dark brown dorsolateral stripes that continue onto the snout and pinkish gray flanks, dorsal surfaces of hindlimbs and arms and venter. Female MUTPL 361 (Fig 19A) had a tan background coloration with dark brown reticulum and the female MUTPL 813 (Fig 19C) had a general grayish coloration with dark markings. Two individuals, MUTPL 813 and 362, additionally had yellowish white spots of different sizes on their dorsum and flanks (Fig 19C and 19D). Several individuals had a large dark X-shaped marking on their back which in case of the female MUTPL 361(Fig 19A), male MUTPL 362 (Fig 19D) and juvenile MUTPL 733 (Fig 19F) is more or less similar to an “hour-glass” shaped marking. The male MUTPL 360 (Fig 19E) had a general dark brown coloration.

**Advertisement call**. Unknown.

**Distribution**. *Pristimantis matildae* is known only from Abra de Zamora and the Cajanuma sector from the Parque Nacional Podocarpus, about 13 km to the south (Fig 6). We were not able to encounter this species in adjacent areas, with similar ecosystems. However, about 35 km south from the type locality, we found another bromeliad specialist from the *P*. *colodactylus* subgroup, a genetically and morphologically similar, undescribed species. *Pristimantis matildae* was encountered at an altitudinal range between 2800 and 3360 m a.s.l. in subpáramo (Fig 2B) and shrub páramo ecosystems.

**Natural history**. This is a common species and one of the most abundant species encountered inside of bromeliads. All the specimens were encountered in terrestrial or arboreal bromeliads or in their close proximity (perching on the leaves), during the day or night. Similarly to *P*. *colodactylus* and *P*. *muranunka*, this species displayed the same defensive behavior of rapidly retreating to the water from the bromeliads when threatened. No calling males were heard.

**Conservation status**. *Pristimantis matildae* is known from only two close localities, from a small area estimated to be less than 50 km^2^. Nonetheless, we recommend that this species to be categorized as Near Threatened following the IUCN criteria [47], due the fact that it is a relatively abundant species and its habitat currently does not face any major threats (because most of the population is situated within a national protected area—Parque Nacional Podocarpus).

**Remarks**. *Pristimantis matildae* is part of the *P*. *colodactylus* subgroup and is closely related to *P*. *colodactylus*, *P*. *muranunka* and an undescribed species (Fig 7). The genetic distance between *P*. *matildae* and its closest relatives from the *P*. *colodactylus* subgroup ranges between 5.4–5.8% (*P*. *colodactylus*) and 6.6–7.5% (*P*. *muranunka*) (S3 Table).

Subgenus ***Huicundomantis*** Páez and Ron, 2019

Four species of this subgenus were described from Abra de Zamora.

### Phylogeny

**Fig 21:**
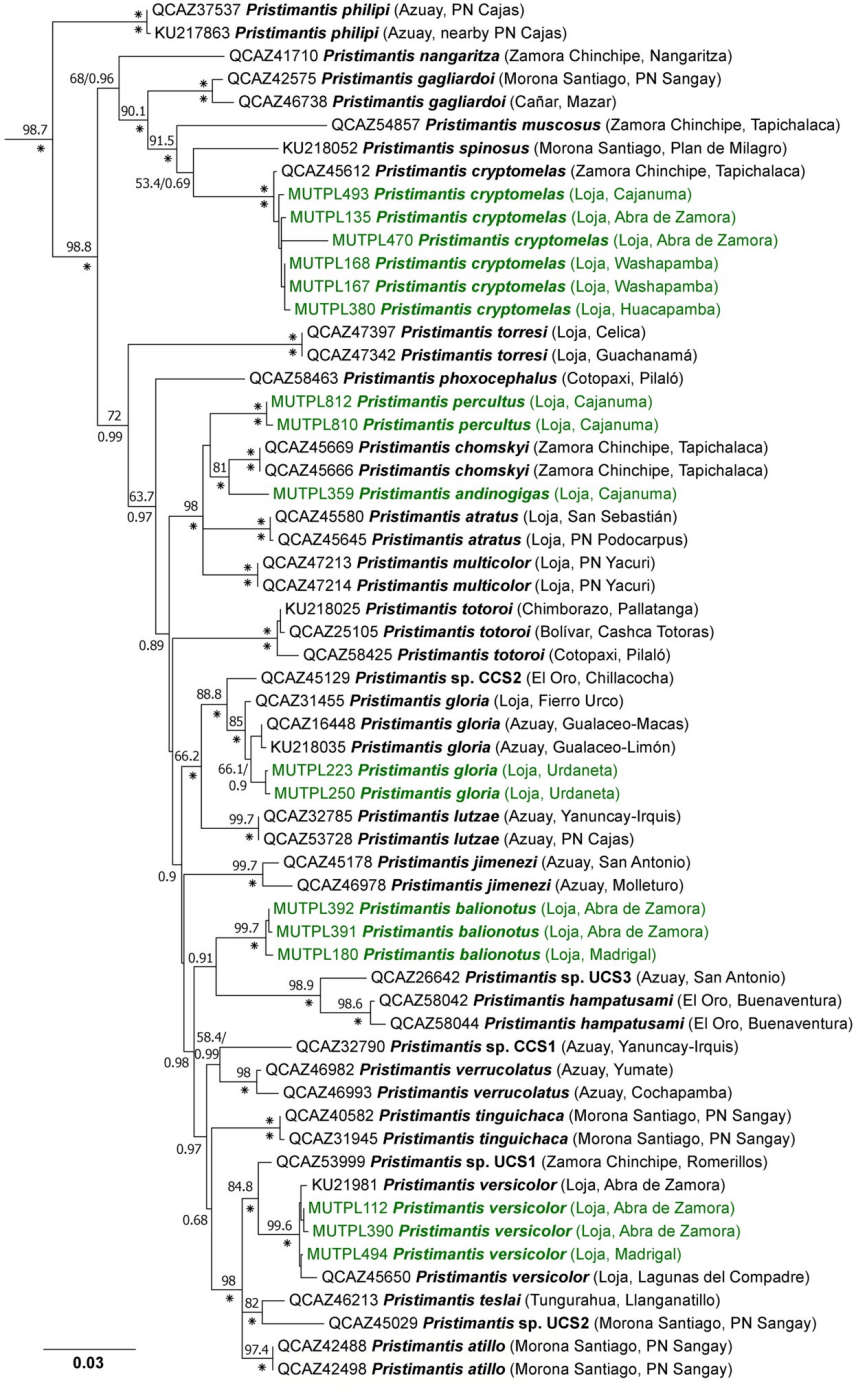
Maximum likelihood phylogram of the *Huicundomantis* subgenus of *Pristimantis* based on 2463 base pairs of concatenated DNA from *12S*, *16S*, and *RAG-1* gene fragments. Bootstrap values (%) and Bayesian posterior probabilities (decimal) are shown except when they are below 50 (bootstrap) or 0.5 (posterior probability); asterisks indicate support values of 100% or 1 (posterior probabilities). Outgroup is not shown; the tree was routed with *Pristimantis galdi*. With green are marked the newly generated sequences for the present study. The collection number, species name, province and short locality name of the samples are shown next to each terminal; all samples are from Ecuador (associated data listed in S2 Table). Abbreviations: CCS = confirmed candidate species, UCS = unconfirmed candidate species [30].

For the *Huicundomantis* subgenus PartitionFinder under AICc (for ML) identified three partition schemes as the best strategy (best model in parentheses): *12S* and *16S* (GTR+I+G), *RAG-1* 1^st^ position (HKY+G), and *RAG-1* 2^nd^ and 3^rd^ positions (TIM+I+G). Under BIC (for BI) PartitionFinder identified three partitions: *12S* and *16S* (GTR+I+G), *RAG-1* 1^st^ position (K80+G), and *RAG-1* 2^nd^ and 3^rd^ positions (K81UF+I). The phylogenetic trees constructed by Bayesian inference and Maximum likelihood showed mostly the same topology with differences in the position of some of the unresolved branches as *P*. *atratus*, *P*. *totoroi*, *P jimenezi*, and the *P*. *teslai*/*P*. UCS2 subgroup (Fig 21). In general, the branches had stronger support in the case of the Bayesian inference.

Similarly to the results of Páez & Ron [30], we recovered the *Huicundomantis* subgenus as monophyletic with strong support (bootstrap values = 98.7%; posterior probabilities = 1) in both Maximum likelihood and Bayesian analyses (Fig 21). However, in our analysis, the *P*. *cryptomelas* group had a strong support only in the case of the Bayesian inference, the ML analysis yielding moderate support. Overall, the phylogenetic tree of our analysis showed the same topology with the one constructed by Páez & Ron [30]. The main differences were in the position of several unresolved branches such as *P*. *jimenezi*, *P*. *phoxocephalus*, *P*. *totoroi* and *P*. *tinguichaca*. Additionally, with the inclusion of the newly generated *P*. *cryptomelas* sequences, this species position, relative to *P*. *spinosus*, became weakly supported (BI) or only moderately supported (ML) (Fig 21).

Our analysis confirmed the membership of *P*. *percultus*, *P*. *andinogigas*, and *P*. *balionotus* to the *P*. *phoxocephalus* species group. In the case of *P*. *andinogigas* the pairwise genetic distance from its closest congener, *P*. *chomskyi*, it was only 2.3% (S3 Table) thus a careful revision of these species statuses is needed.

### *Pristimantis cryptomelas* species group

One species of this species group was described from Abra de Zamora.

***Pristimantis cryptomelas*** (Lynch, 1979)

(Fig 22)

**Fig 22:**
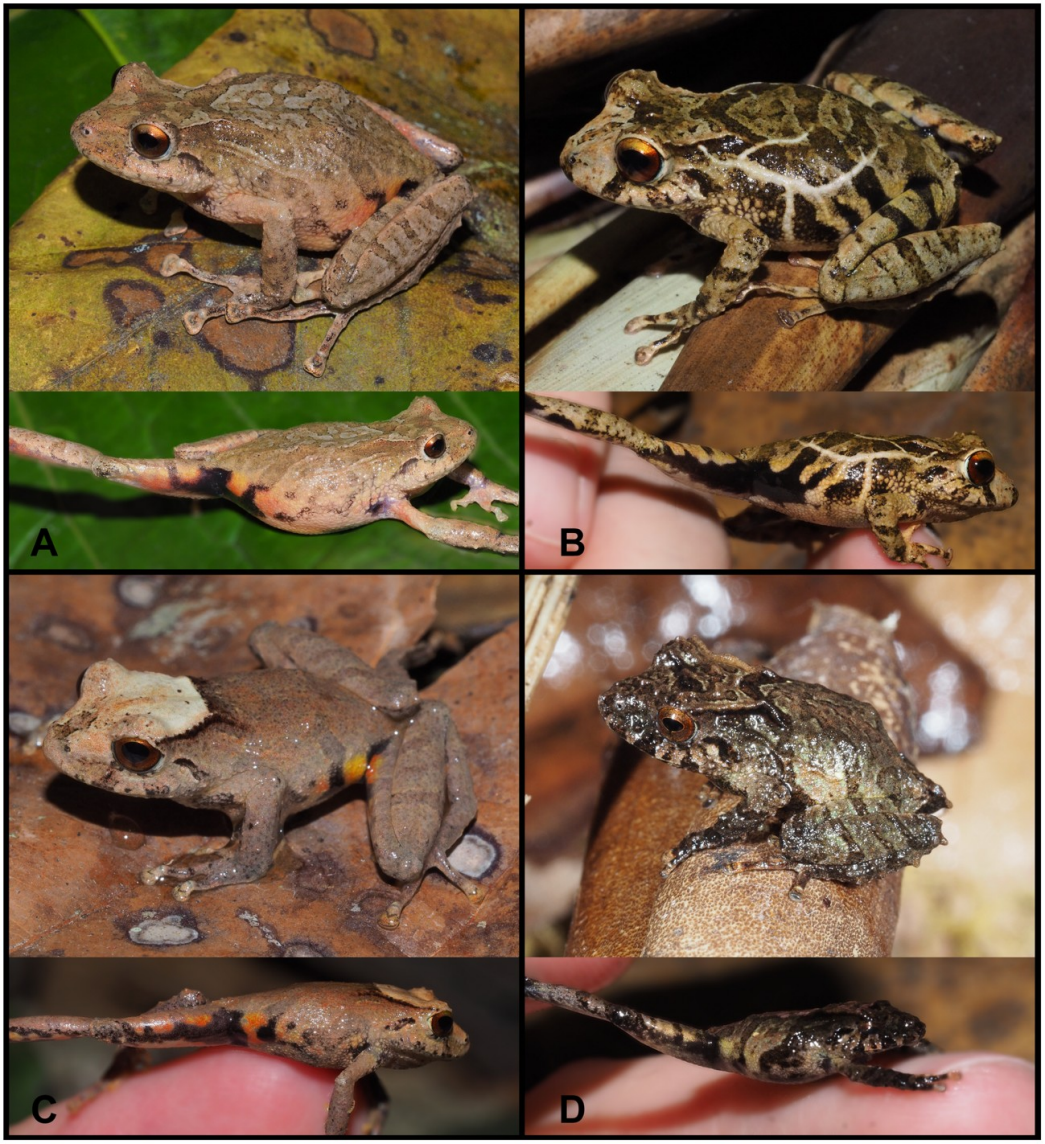
Color variation of *Pristimantis cryptomelas* in life. **A**. female: MUTPL 168; **B**. male: MUTPL 470; **C**. subadult: MUTPL 135; **D**. juvenile: MUTPL 493. Abra de Zamora (B, C), Bosque Protector Washapamba (A), Parque Nacional Podocarpus—Cajanuma (D). Most common coloration of this species is presented in A.

*Eleutherodactylus cryptomelas* Lynch, 1979

*Eleutherodactylus* (*Eleutherodactylus*) *cryptomelas*: Lynch, 1996; Lynch and Duellman, 1997

*Pristimantis cryptomelas*: Heinicke, Duellman, and Hedges, 2007

*Pristimantis* (*Pristimantis*) *cryptomelas*: Hedges, Duellman, and Heinicke, 2008

*Pristimantis* (*Huicundomantis*) *cryptomelas*: Páez and Ron, 2019

**Common English name**. Cryptic Robber Frog

**Common Spanish name**. Cutín críptico

**Etymology**. The specific name is derived from the Greek *kryptos*, meaning “hidden” and the Greek *melas*, meaning “black” and refers to the black areas in the axilla and groin and on the hidden surfaces of the limbs [25].

**Holotype**. KU 141992, an immature female, taken 15 km E Loja, Provincia Zamora-Chinchipe, Ecuador, 2710 m, May 1971 by William E. Duellman.

**Paratypes**. KU 141993, taken syntopically with holotype; KU 120095–96, 8–9 km N San Lucas, Provincia Loja, Ecuador, 3000 m; USNM 198480–82, Sapote, Provincia Morona-Santiago, Ecuador, 2470 m; USNM 198483, 2 km W Sapote, Provincia Morona-Santiago, Ecuador, 2560 m.

**Diagnosis**. *Pristimantis cryptomelas* is a medium sized species distinguished by the following combination of traits: (1) skin on dorsum shagreened; skin on venter areolate; discoidal fold weak; dorsolateral folds absent; low middorsal fold present; (2) tympanic membrane absent but tympanic annulus evident, oval, its length about 30% of the length of eye; supratympanic fold present; (3) snout subacuminate in dorsal view, rounded in profile; canthus rostralis straight in dorsal view, rounded in profile; (4) upper eyelid bearing 1 to 3 larger tubercles (trait more visible in life), about 84% IOD; cranial crests absent but prominent) (or > < -shaped scapular folds are present (trait more visible in life); (5) dentigerous processes of vomers prominent, oblique, ovoid, separated medially by distance equal or lower than the width of processes; each processes bearing 3 to 5 teeth; (6) males lacking vocal sac and slits; nuptial pads absent; (7) Finger I shorter than Finger II; discs on fingers broadly expanded, truncated; circumferential grooves present; fingers bearing narrow lateral fringes; (8) subarticular tubercles prominent; supernumerary palmar tubercles evident; palmar tubercle partially (sometimes completely) divided into a larger (inner) and a smaller (outer) tubercles; thenar tubercle elliptical, smaller than the palmar tubercle but larger than either palmar tubercles (inner or outer); (9) prominent ulnar tubercles present (trait more visible in life); (10) heel with one large, conical and several smaller tubercles; outer edge of tarsus bearing a row of enlarged spine like tubercles (trait more visible in life); inner tarsal tubercles coalesced into a short tarsal fold; (11) inner metatarsal tubercle elongated, not compressed, about 4 to 6x ovoid, subconical (in profile), outer metatarsal tubercle; supernumerary plantar tubercles present; (12) Toe V much longer than Toe III; discs on toes broadly expanded, truncated, slightly smaller than those of fingers; circumferential grooves present; toes bearing narrow lateral fringes; webbing basal; (13) in life, dorsum gray to brown with sparse darker markings or chevrons; venter white to cream with brown reticulations; hindlimbs usually with dark transverse bars; axilla, groin, anterior and posterior surfaces of thighs, and concealed surfaces of shanks black bordered by white, yellow or orange blotches; iris dark bronze with a reddish median horizontal streak; (14) SVL 28.9–45.7 mm in adult females (35.3 ± 9.06 SD, *n* = 3) and 25.6–31.2 mm in adult males (*n* = 2).

**Variation**. Morphometric variation is shown in Table 3. The females are significantly larger than the males, with one female (MUTPL 168, Fig 22A) having 45.7 mm SVL. All encountered specimens had the groin (but frequently also the axilla, anterior and posterior surfaces of thighs, and concealed surfaces of shanks) black, with the bordering blotches (or stripes) varying from reddish orange (Fig 22A and 22C), yellow (Fig 22B) to whitish yellow (Fig 22D). Manny encountered individuals had the coloration of the female MUTPL 168 (Fig 22A), with brown or light brown dorsum and whitish, light markings. A few individuals had some white, branch-like white markings on their flanks and dorsum like in the case of the male MUTPL 470 (Fig 22B), and several individuals had the head and snout distinctly colored (white or beige) compared to the rest of the brownish body (MUTPL 135, Fig 22C). The juveniles had a more rugged general appearance and it seems that they lack the intense yellow or orange coloration of the inguinal and axial blotches (MUTPL 493, Fig 22D).

**Table 3:** Body mass (in grams), measurements (in mm) and morphological proportions (in percentages) of adult females and males of the species from the Subgenus *Huicundomantis* described from Abra de Zamora.

Character	*Pristimantis cryptomelas*		*Pristimantis percultus*		*Pristimantis balionotus*		*Pristimantis versicolor*	
	females (*n* = 3)	males (*n* = 2)	females* (*n* = 3)	males* (*n* = 1)	females* (*n* = 2)	males (*n* = 6)	females (*n* = 3)	males (*n* = 4)
Body mass (BM)	3.39 ± 2.69 (1.68–6.49)	1.19–1.84	2.08–2.52	-	2.48	1.12 ± 0.21 (0.88–1.44)	2.13 ± 0.69 (1.62–2.92)	1.05 ± 0.13 (0.96–1.24)
Snout-vent length (SVL)	35.3 ± 9.06 (28.9–45.7)	25.6–31.2	33.6 ± 4.15 (30.1–38.2)	29.8	28.4–32.4	24.7 ± 0.83 (23.6–25.8)	30.8 ± 1.40 (29.4–32.2)	24.0 ± 1.08 (22.9–25.4)
Head width (HW)	12.5 ± 3.38 (10.5–16.4)	9.6–11.6	13.1 ± 1.71 (11.7–15.0)	12.1	11.3–11.5	9.0 ± 0.47 (8.4–9.8)	11.5 ± 1.26 (10.3–12.8)	8.8 ± 0.39 (8.4–9.2)
Head length (HL)	11.6 ± 3.15 (9.7–15.2)	7.6–9.5	11.9 ± 1.30 (10.6–13.2)	11.0	10.4–10.7	8.7 ± 0.50 (7.9–9.3)	10.3 ± 0.95 (9.4–11.3)	8.0 ± 0.84 (7.2–8.9)
Interorbital distance (IOD)	4.0 ± 1.00 (3.2–5.1)	2.8–3.4	4.0 ± 0.30 (3.7–4.3)	3.9	2.8–4.0	3.0 ± 0.49 (2.6–3.9)	3.3 ± 0.27 (3.1–3.6)	2.5 ± 0.19 (2.4–2.8)
Internarial distance (IND)	2.7 ± 0.46 (2.4–3.2)	2.1–2.5	2.4–2.6	-	2.4	2.0 ± 0.09 (1.9–2.1)	2.4 ± 0.17 (2.2–2.5)	1.9 ± 0.08 (1.8–2.0)
Upper eyelid width (EW)	3.3 ± 0.78 (2.7–4.2)	2.3–2.9	3.0 ± 0.21 (2.8–3.2)	3.4	2.6–3.1	2.5 ± 0.09 (2.4–2.6)	2.8 ± 0.10 (2.7–2.9)	2.5 ± 0.06 (2.4–2.5)
Eye diameter (ED)	4.0 ± 1.19 (3.3–5.4)	3.0–3.6	3.8 ± 0.55 (3.4–4.4)	3.5	3.3	2.9 ± 0.14 (2.8–3.1)	3.5 ± 0.15 (3.3–3.6)	2.9 ± 0.06 (2.8–2.9)
Eye-nostril distance (EN)	3.6 ± 1.29 (2.5–5.0)	2.4–3.5	3.6 ± 0.58 (3.3–4.3)	3.4	3.6–4.0	2.7 ± 0.15 (2.5–2.9)	3.1 ± 0.15 (3.0–3.3)	2.6 ± 0.22 (2.3–2.8)
Tympanum diameter (TD)	1.1 ± 0.21 (0.9–1.3)	0.9–1.1	1.8 ± 0.20 (1.6–2.0)	1.5	1.7–1.8	1.4 ± 0.11 (1.3–1.6)	1.6 ± 0.15 (1.4–1.7)	1.3 ± 0.08 (1.2–1.4)
Femur length (FL)	16.1 ± 4.16 (13.6–20.9)	12.3–14.7	15.2–17.3	-	13.3	10.6 ± 0.30 (10.2–10.8)	14.5 ± 1.05 (13.5–15.6)	11.0 ± 0.44 (10.4–11.4)
Tibia length (TL)	18.3 ± 3.92 (15.5–22.8)	14.4–15.2	18.6 ± 2.33 (16.7–21.2)	15.5	12.8–14.3	11.5 ± 0.24 (11.2–11.9)	16.4 ± 0.72 (15.8–17.2)	12.6 ± 0.72 (11.8–13.5)
Foot length (FoL)	17.4 ± 4.77 (14.1–22.9)	12.4–15.3	16.0–16.6	-	13.1	9.8 ± 0.30 (9.3–10.2)	14.6 ± 1.23 (13.7–16.0)	11.4 ± 0.81 (10.4–12.3)
Hand length (HaL)	11.2 ± 3.71 (8.5–15.4)	8.2–9.3	9.6–10.8	-	7.7	6.4 ± 0.33 (6.0–6.9)	9.8 ± 1.15 (8.9–11.1)	7.3 ± 0.43 (6.9–7.9)
HW/SVL	33.8–36.3	37.2–37.5	38.7–39.3	40.6	34.9–40.5	34.1–38.1	35.0–39.8	36.2–37.0
HL/SVL	31.2–33.6	29.7–30.4	34.6–36.8	36.9	32.1–37.7	31.7–36.8	32.0–35.1	31.1–35.0
HL/HW	92.4–92.7	79.2–81.9	88.0–95.2	90.9	92.0–93.0	88.8–98.9	88.3–91.3	85.9–96.7
EN/HL	25.8–32.9	31.6–36.8	27.5–32.6	30.9	33.6–38.5	30.1–34.2	29.2–31.9	31.5–34.2
ED/HL	33.7–35.5	37.9–39.5	29.2–33.3	31.8	30.8–31.7	31.1–36.7	31.9–35.1	32.6–38.9
EW/IOD	82.4–86.1	82.1–85.3	72.5–75.7	87.2	65.0–110.7	66.7–96.2	80.6–87.5	89.3–104.2
EN/ED	73.5–97.0	80.0–97.2	94.3–97.7	97.1	109.1–121.2	87.1–103.6	88.6–91.7	82.1–96.6
TD/ED	22.2–39.4	30.0–30.6	45.5–51.4	42.9	51.5–54.5	44.8–51.6	42.4–47.2	42.9–48.3
FL/SVL	43.9–47.1	47.1–48.0	50.5–53.1	-	41.0	41.9–43.9	45.9–48.4	44.3–47.2
TL/SVL	49.9–53.6	48.7–56.3	54.9–55.5	52.0	44.1–45.1	44.6–49.8	52.4–53.7	50.2–53.7
FoL/SVL	48.7–50.1	48.4–49.0	50.9–53.2	-	40.4	38.4–42.7	45.6–49.7	45.4–48.4
HaL/SVL	29.4–33.7	29.8–32.0	31.9–33.1	-	23.8	24.1–26.7	30.3–34.5	30.0–31.1

Values are given as mean ± SD (range). Female body mass includes eggs. *—indicates that some measurements were included from the original descriptions [9].

**Advertisement call**. Unknown.

**Fig 23:**
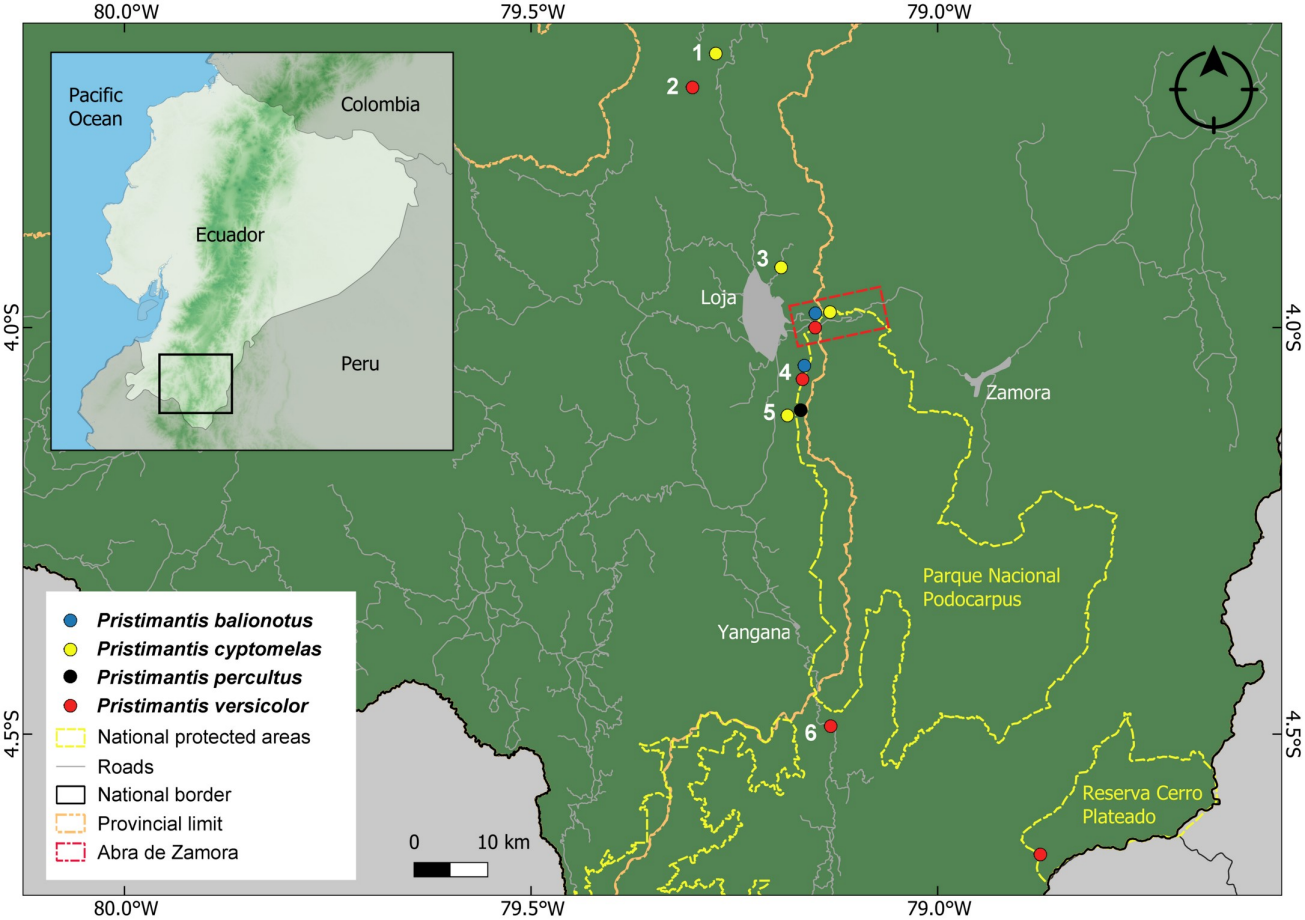
Distribution of the species of the *Huicundomantis* subgenus described from Abra de Zamora. Records are based on specimens (and DNA sequences) deposited at the Museo de Zoología, Universidad Técnica Particular de Loja (MUTPL). 1. Bosque Protector Washapamba, 2. Ramos Urcu, 3. Huacapamba, 4. Reserva Madrigal del Podocarpus, 5. Parque Nacional Podocarpus (Cajanuma sector), 6. Reserva Tapichalaca.

**Distribution**. *Pristimantis cryptomelas* presence is confirmed (with DNA samples) from Reserva Tapichalaca, Parque Nacional Podocarpus (Cajanuma entrance), Abra de Zamora, Huacapamba (5 km north to the city of Loja) and Bosque Protector Washapamba, in Loja and Zamora Chinchipe provinces (Fig 23). The species has been reported from Morona Santiago province [9] and Peru [25]; however, due to the large distance between the sites, we recommend genetic analyses to confirm that these populations in fact belong to *P*. *cryptomelas*. The species was encountered at an altitudinal range between 2480 and 2960 m a.s.l. in montane cloud forest, evergreen upper montane forest (Fig 2C) and subpáramo ecosystems.

**Natural history**. This is a common species, even in habitats with anthropic disturbance. All specimens were encountered during the night, perching on the vegetation (usually at 30 cm–2 m above the ground), sometimes in the vicinity of streams. No calling males were heard.

**Conservation status**. *Pristimantis cryptomelas* is currently categorized as Near Threatened following the IUCN criteria [51]. We consider this category as adequate due the fact that it is a relatively abundant species and some of its populations are inside protected areas (Parque Nacional Podocarpus, Bosque Protector Washapamba), but also because it is currently known from only 5 localities, the existing populations are severely fragmented and there is a continuing decline in the extent and quality of its habitat due to cattle grazing and deforestation.

**Remarks**. Lynch [9] provides a detailed description of this species. Our diagnosis concurs with most of the morphological features described by the author, and we add descriptions of the characters that are more evident in life. Some of the differences that we observed are: tympanum oval (in contrast to the round tympanum described by Lynch); discoidal fold weak (discoidal folds prominent); upper eyelid bearing 1 to 3 larger tubercles (upper eyelids tuberculate, no tubercle greatly enlarged). The diagnosis provided herein is based on 8 specimens from the original description (KU 141992, 141993, 120095, 120096, 198480–83), one adult male (MUTPL 470) and two subadults (MUTPL 135, 471) collected from the type locality and 10 specimens collected from the other localities.

### *Pristimantis phoxocephalus* species group

Three species of this species group were described from Abra de Zamora.

***Pristimantis percultus*** (Lynch, 1979)

(Fig 24)

**Fig 24:**
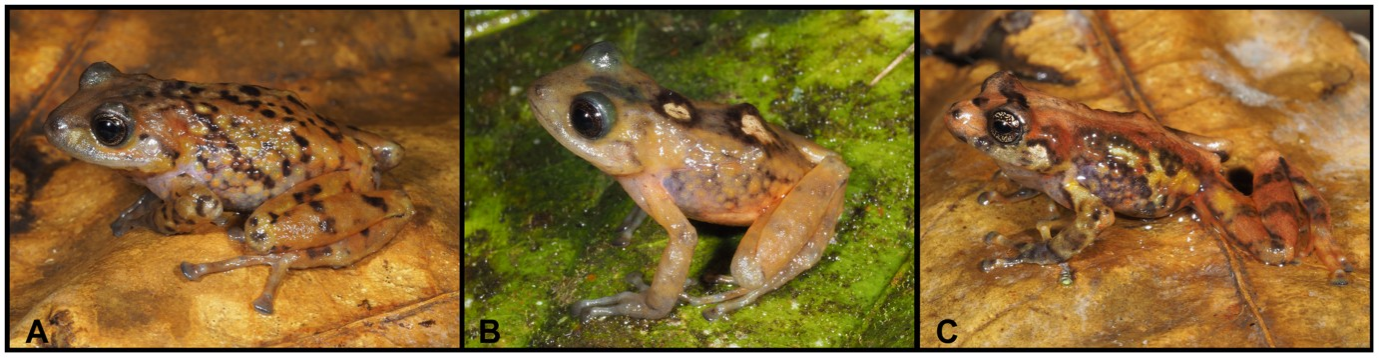
Color variation of *Pristimantis percultus* in life. **A–B** females: **A**. MUTPL 810, **B**. MUTPL 812; **C**. subadult: MUTPL 811. All specimens are from Parque Nacional Podocarpus—Cajanuma.

*Eleutherodactylus percultus* Lynch, 1979

*Eleutherodactylus* (*Eleutherodactylus*) *percultus*: Lynch, 1996; Lynch and Duellman, 1997

*Pristimantis percultus*: Heinicke, Duellman, and Hedges, 2007

*Pristimantis* (*Huicundomantis*) *percultus*: Páez and Ron, 2019

**Common English name**. Zamora Robber Frog

**Common Spanish name**. Cutín de páramo bajo

**Etymology**. Latin, meaning highly adorned, in reference to the orange lip stripes.

**Holotype**. KU 166058, an adult male, found in Abra de Zamora, Provincia Zamora-Chinchipe, Ecuador, 2850 m, on 8 March 1975 by Dana K. Duellman.

**Paratype**. KU 166057, an adult female topotype, obtained on 7 March 1975.

**Diagnosis**. *Pristimantis percultus* is a medium sized species distinguished by the following combination of traits: (1) skin on dorsum and flanks pustulated, covered with large flat warts or pustules (in life the pustulated skin texture is more evident); skin on venter coarsely areolate; discoidal fold weak; dorsolateral folds absent; (2) tympanic annulus evident and tympanic membrane differentiated, its length about 47% of the length of eye; supratympanic fold present; (3) snout subacuminate in dorsal view, rounded in profile, with pointed tip; canthus rostralis weakly concave in dorsal view, rounded in profile; (4) upper eyelid bearing one larger and several small tubercles (trait more visible in life), about 74% IOD in females and 87% IOD in males; barely visible, low cranial crests present; (5) dentigerous processes of vomers prominent, oblique, triangular, separated medially by distance lower than the width of processes; each processes bearing 4 to 8 teeth; (6) males lacking vocal sac and vocal slits; (7) Finger I shorter than Finger II; discs on fingers broadly expanded, rounded; circumferential grooves present; fingers bearing narrow lateral fringes; (8) subarticular tubercles prominent, round, non-conical; supernumerary palmar tubercles present; palmar tubercle bifid, partially divided into a larger (inner) and a smaller (outer) tubercles; thenar tubercle elliptical, smaller than the palmar tubercle, but larger than either palmar tubercles (inner or outer); (9) small, inconspicuous, ulnar tubercles present (trait more visible in life); (10) heel with one large and several smaller rounded tubercles; outer edge of tarsus with a row of large conical tubercles; inner tarsal tubercles coalesced into a short tarsal fold; (11) inner metatarsal tubercle broadly ovoid, about 4x ovoid, outer metatarsal tubercle; supernumerary plantar tubercles present; (12) Toe V much longer than Toe III; discs on toes broadly expanded, rounded, about same size as those on fingers; circumferential grooves present; toes bearing narrow lateral fringes; webbing basal; (13) in life, dorsum brown or beige with or without dark brown flecking and marbling or large white spots; lips sometimes with orange strips; venter cream with or without dark flecking; no markings in axilla, groin or on concealed limb surfaces; iris bronze with dense black reticulations; (14) SVL 30.1–38.2 mm in adult females (33.6 ± 4.15 SD, *n* = 3; [9]) and 29.8 mm in one adult male [9].

**Variation**. Morphometric variation is shown in Table 3. The coloration of female MUTPL 810 (Fig 24A) is similar with that of the holotype KU 166058, with the exception of the orange strips on the lips. Neither of the encountered specimens had the lips colored. The female MUTPL 812 (Fig 24B) had the similar large white spots bordered by dark brown borders like the specimen KU 202427. The subadult MUTPL 811 (Fig 24C) had a peculiar coloration with dorsum light brown and flanks multicolored with yellow, green, brown and pinkish gray blotches. Also, this specimen was the only one that had dark interorbital bars. In all encountered specimens the dorsum and especially the flanks pustulated texture it was very evident. Most of the pustules had the yellowish orange color similar with those of the holotype. Additionally, all three specimens presented some scar-like wrinkles on their flanks (more evident in the case of MUTPL 812, Fig 24b).

**Advertisement call**. Unknown.

**Distribution**. Despite intensive searches carried out since 2016 we did not find any individuals in the type locality, Abra de Zamora. *Pristimantis percultus* was reported from Parque Nacional Podocarpus (Cajanuma sector) in 2010 [52], in a grass páramo ecosystem [44], from about 12 km south to Abra de Zamora (Fig 23). We also encountered there in 2019. The species was recorded at an altitudinal range between 2850 and 3380 m a.s.l. in subpáramo and grass páramo ecosystems.

**Natural history**. This is a rare or hard to detect species. Lynch [9] described the species with only two specimens and only four more specimens were collected afterwards, in 1984 by W.E. Duellman, all from the type locality. Aguirre Mendoza et al. [52] found only seven individuals through 21 sampling days, from December 2009 to April 2010, in Parque Nacional Podocarpus (Cajanuma sector). We encountered two females and a subadult, all during one night of our ongoing fieldwork carried out in this area since 2018. All specimens were encountered during the night, perching on the vegetation (on leaves of *Neurolepis* sp., *Valeriana* sp. or of bromeliads) at 10 to 40 cm above the ground. No calling males were heard.

**Conservation status**. *Pristimantis percultus* is currently categorized as Endangered based on criteria B1ab(iii) [53]. We suggest maintaining this category because, its Extent of occurrence (EOO) and Area of occupancy (AOO) are estimated to be less than 50 km^2^, it is known from only two locations and it is possible that there is a decline in the number of locations or subpopulations.

**Remarks**. Lynch [9] provides a detailed description of this species. Our diagnosis concurs with all the morphological features described by the author, to which we add descriptions of the characters that are more evident in life. The diagnosis provided herein is based on two specimens from the original description (KU 166057, 166058), two adult females (MUTPL 810, 812) and one subadult (MUTPL 811) collected from about 12 km south to the type locality.

*Pristimantis percultus* is part of the *P*. *phoxocephalus* group, and is closely related to *P*. *chomskyi*, *P*. *andinogigas*, *P*. *atratus* and *P*. *multicolor*. However, its exact kinship to these species remains non-resolved (Fig 21). The genetic distance between *P*. *percultus* and its closest relatives ranges between 3.3–3.7% (*P*. *chomskyi*) and 4% (*P*. *multicolor*) (S3 Table).

***Pristimantis balionotus*** (Lynch, 1979)

(Fig 25)

**Fig 25:**
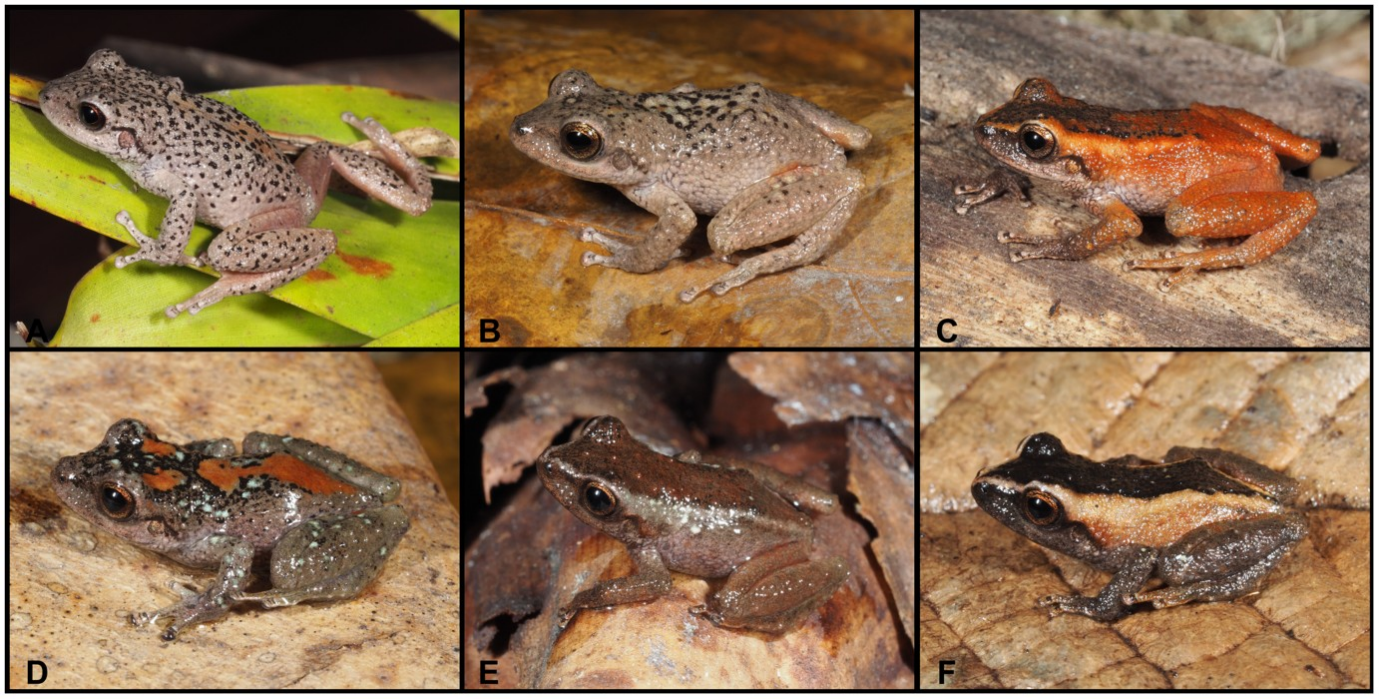
Color variation of *Pristimantis balionotus* in life. **A**. female: MUTPL 392; **B–C** males: **B**. MUTPL 677, **C**. MUTPL 487; **D–F** juveniles: **D**. MUTPL 489, **E**. MUTPL 490, **F**. MUTPL 491. Abra de Zamora (A, B), Reserva Madrigal del Podocarpus (C, D, E, F). Most common coloration of this species is presented in B.

*Eleutherodactylus balionotus* Lynch, 1979

*Eleutherodactylus* (*Eleutherodactylus*) *balionotus*: Lynch and Duellman, 1997

*Pristimantis balionotus*: Heinicke, Duellman, and Hedges, 2007

*Pristimantis* (*Pristimantis*) *balionotus*: Hedges, Duellman, and Heinicke, 2008

*Pristimantis* (*Huicundomantis*) *balionotus*: Páez and Ron, 2019

**Common English name**. Speckled Rain Frog (This is a newly proposed name as it is more appropriate than the previous one, “Crest Robber Frog”).

**Common Spanish name**. Cutín de lomo manchado

**Etymology**. Greek, meaning speckled back, in reference to the predominant color pattern.

**Holotype**. KU 142136, an adult female from 13.5 km E Loja, at the crest of the Cordillera (Abra de Zamora) between Provincia Loja and Provincia Zamora-Chinchipe, Ecuador, 2800 m, obtained on 22 July 1971 by William E. Duellman.

**Paratypes**. KU 142135, KU 142137–44, collected syntopically with the holotype.

**Diagnosis**. *Pristimantis balionotus* is a medium sized species distinguished by the following combination of traits: (1) skin on dorsum tuberculated (in life the skin tuberculated texture is more evident); skin on venter coarsely areolate; discoidal fold weak; dorsolateral folds absent; (2) tympanic annulus evident and tympanic membrane differentiated, its length about 49% of the length of eye; supratympanic fold present; (3) snout subacuminate in dorsal view, rounded in profile; canthus rostralis weakly concave in dorsal view, rounded in profile; (4) upper eyelid bearing several small tubercles, similar in size and shape with the ones from the dorsum, about 88% IOD in females and 84% IOD in males; cranial crests absent; (5) dentigerous processes of vomers prominent, oblique, ovoid, separated medially by distance equal or lower than the width of processes; each processes bearing 2 to 4 teeth; (6) males with a subgular vocal sac and vocal slits; nuptial pads absent; (7) Finger I shorter than Finger II; discs on fingers broadly expanded, rounded; circumferential grooves present; fingers bearing narrow lateral fringes; (8) subarticular tubercles prominent; supernumerary palmar tubercles present; palmar tubercle partially divided into a larger (inner) and a smaller (outer) tubercles; thenar tubercle elliptical, smaller than the inner palmar tubercle; (9) small, inconspicuous, ulnar tubercles present (trait more visible in life); (10) heel with several small, rounded tubercles; outer edge of tarsus with a row of small tubercles; inner tarsal tubercles present, sometimes coalesced into a tarsal fold; (11) inner metatarsal tubercle broadly ovoid, about 4x ovoid, subconical (in profile), outer metatarsal tubercle; supernumerary plantar tubercles present; (12) Toe V slightly longer than Toe III; discs on toes broadly expanded, rounded, about same size as those on fingers; circumferential grooves present; toes bearing narrow lateral fringes; webbing absent; (13) in life, dorsum gray or brown, with black flecks; venter light gray without markings; no markings in axilla, groin or on concealed limb surfaces; iris bronze with a reddish median horizontal streak and fine black reticulations; (14) SVL 28.4–32.4 mm in adult females (*n* = 2; [9]) and 23.6–25.8 mm in adult males (24.7 ± 0.83 SD, *n* = 6).

**Variation**. Morphometric variation is shown in Table 3. Most of the encountered individuals had a generally gray coloration, as was the case of the female MUTPL 392 (Fig 25A) and male MUTPL 677 (Fig 25B). The male MUTPL 677 (Fig 25B) had the same coloration as the holotype KU 142136 (Fig 3B in [9]). Male MUTPL 487 (Fig 25C) was reddish brown, the juvenile MUTPL 489 (Fig 25D) had a reddish brown back and gray flanks, juvenile MUTPL 490 (Fig 25E) had a brown broad middorsal band and gray flanks and juvenile MUTPL 491 (Fig 25F) had a dark brown broad middorsal band and beige flanks. All the encountered individuals had the characteristic black flecks but some had only a few (MUTPL 490, 491), others had many flecks especially on the center of their backs (MUTPL 677, 487, 489), and in some cases all their dorsum and dorsal surfaces of hindlimbs and arms were covered with flecks (MUTPL 392).

**Fig 26:**
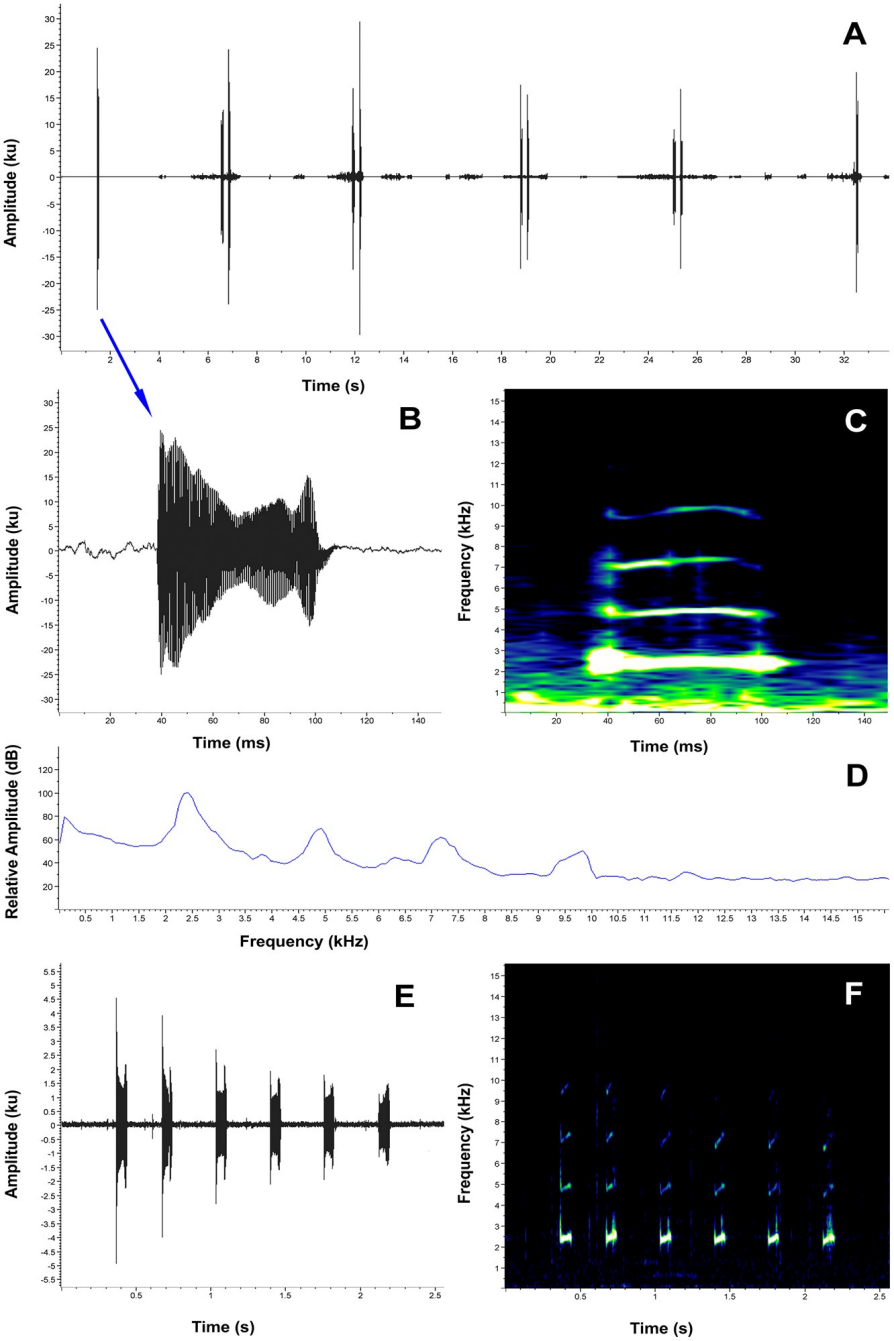
Advertisement call of *Pristimantis balionotus*. **A–D** MUTPL 677, FUTPL-A 232: **A**. Oscillogram of a 6 calls section of the call series, with single and double-noted calls; **B**. Oscillogram of a single-noted call; **C**. Spectrogram of a single-noted call; **D**. Power spectrum of a single-noted call; **E–F** FUTPL-A 230: **E**. Oscillogram of a 6 notes section of a 8-noted call; **F**. Spectrogram of a 6 notes section of a 8-noted call.

**Advertisement call**. The advertisement call of one male from Reserva Madrigal del Podocarpus was recorded in 2018 and of two males from Abra de Zamora were recorded in 2019. Descriptive statistics of the acoustic variables are provided in Table 2 (the detailed information of each of the separate recordings is presented in the S4 Table). *Pristimantis balionotus* has an advertisement call characterized by a call series composed by whistles repeated over a period of time, somewhat similar to the calls of *P*. *jimenezi* and *P*. *phoxocephalus* (Fig 26). The calls are composed usually by one or two notes (frequency modulated tonal sounds), but sometimes (probably in case of social interactions, triggered by the nearby presence of a female or a competitive male) the males can emit calls with up to 8 notes (FUTPL-A 230, Fig 26E and 26f). The calls are characterized by a mean duration of 0.069 s (SD = 0.005), a mean inter-call interval of 6.075 s (SD = 2.371) and a mean call rate of 10.0 calls/min (SD = 1.610). In the case of the double-noted calls the call duration was on average 0.355 s (SD = 0.110) and had a mean note rate of 3.56 notes/s (SD = 0.126) (S4 Table). The mean dominant frequency of the call was 2431.7 Hz (SD = 59.371), with a mean 90% bandwidth of 2331.8–2542.8 Hz (Table 2). The fundamental frequency is not recognizable, but usually 4 harmonics are visible.

**Distribution**. *Pristimantis balionotus* is known only from Abra de Zamora and about 9 km to the south, in Reserva Madrigal del Podocarpus, Loja province (Fig 23). We encountered most of the specimens, from Abra de Zamora, outside of Parque Nacional Podocarpus. The species was encountered at an altitudinal range between 2770 and 2960 m a.s.l., in subpáramo ecosystems (Fig 2B).

**Natural history**. This is an uncommon species. Lynch [9] described the species based on 10 specimens, and only one more was collected afterwards, in 1984 by W.E. Duellman. *Pristimantis balionotus* is a bromeliad specialist, all the individuals being found inside or on the leaves of terrestrial bromeliads. The call of this species was recorded infrequently in the months of February, May, July, August, September, November and December, only on nights with high humidity (rain or dense fog). All calling males were found on bromeliads leaves.

**Conservation status**. *Pristimantis balionotus* is currently categorized as Endangered [54] based on criteria B1 ab(iii). We suggest maintaining this category because its Extent of occurrence (EOO) and Area of occupancy (AOO) are estimated to be less than 30 km^2^, it is known from only two locations, with part of its populations found outside protected areas which could be severely affected in the near future by habitat destruction.

**Remarks**. Lynch [9] provides a detailed description of this species. Our diagnosis concurs with all the morphological features described by the author, to which we add descriptions of the characters that are more evident in life. The diagnosis provided herein is based on 10 specimens from the original description (KU 142135–44), one adult female (MUTPL 392) and 4 adult males (MUTPL 292, 297, 391, 677) collected from the type locality, and 5 specimens collected from Reserva Madrigal del Podocarpus.

*Pristimantis balionotus* is part of the *P*. *phoxocephalus* group, but its kinship to the other members of the group remains non-resolved (Fig 21). In our phylogram *P*. *balionotus* is closely related to *P*. *hampatusami* and an undescribed species (UCS3) but with only moderate BI support. The genetic distance between *P*. *balionotus* and its relatives from the *P*. *phoxocephalus* group ranges between 2.3–4.4% (*P*. *gloria*), 2.5–3.5% (*P*. *lutzae*), 3.7–3.9% (*P*. *verrucolatus*), 3.6–4.3% (*P*. *tinguichaca*), 3.8–5.8% (*P*. *hampatusami*) and up to 6.5–8.0% (*P*. *torresi*) (S3 Table).

***Pristimantis versicolor*** (Lynch, 1979)

(Fig 27)

**Fig 27:**
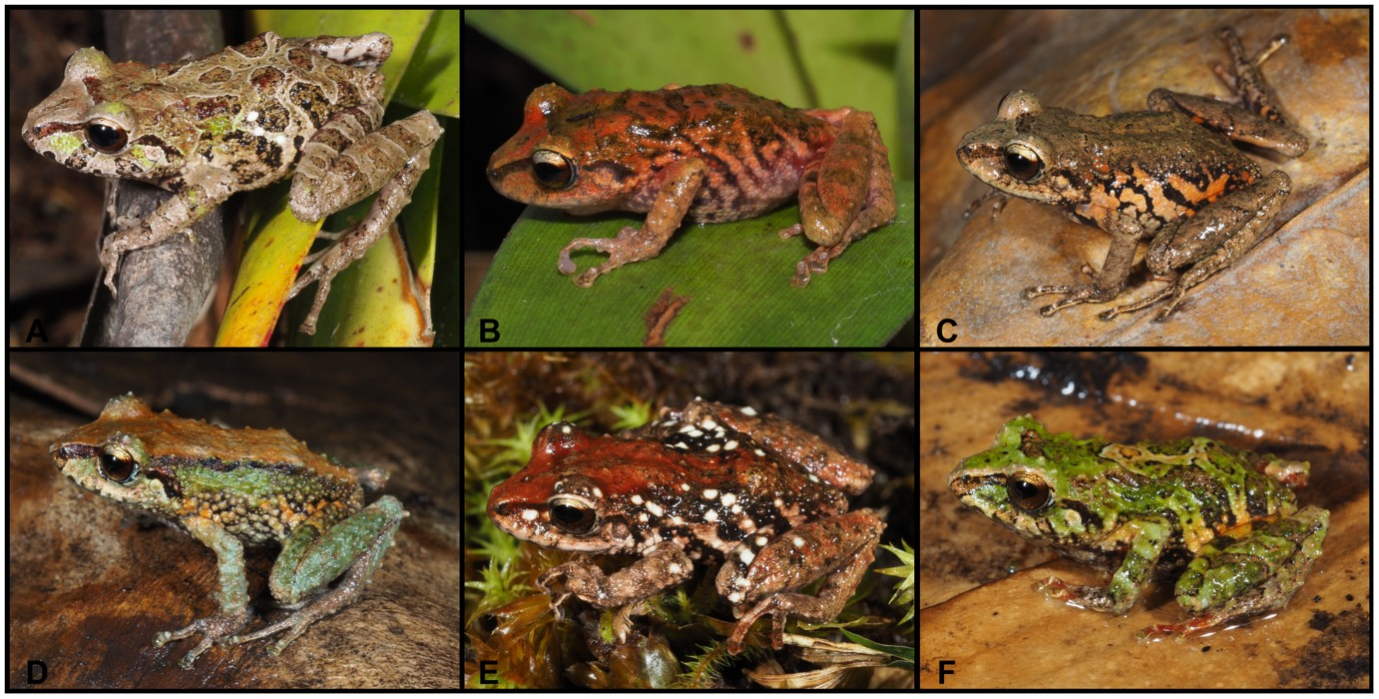
Color variation of *Pristimantis versicolor* in life. **A**. female: MUTPL 390; **B–C** males: **B**. MUTPL 112, **C**. MUTPL 740; **D–E** subadults: **D**. MUTPL 497, **E**. MUTPL 293; **F**. juvenile: MUTPL 313. Abra de Zamora (A, B, D, E, F), Reserva Tapichalaca (C). Most common coloration of this species is presented in A and F.

*Eleutherodactylus versicolor* Lynch, 1979

*Eleutherodactylus* (*Eleutherodactylus*) *versicolor*: Lynch, 1996; Lynch and Duellman, 1997

*Pristimantis versicolor*: Heinicke, Duellman, and Hedges, 2007

*Pristimantis* (*Pristimantis*) *versicolor*: Hedges, Duellman, and Heinicke, 2008

*Pristimantis* (*Huicundomantis*) *versicolor*: Páez and Ron, 2019

**Common English name**. Variegated Rain Frog (This is a newly proposed name as it is more appropriate than the previous one, “Loja Robber Frog”).

**Common Spanish name**. Cutín versicolor

**Etymology**. The specific name is a Latin adjective meaning of different colors or variegated [25].

**Holotype**. KU 119858, an adult female [25] obtained 13.5 km E. Loja, 2800 m, a locality just east of the crest on the mountain range dividing Loja and Zamora-Chinchipe provinces, Ecuador, on 10 June 1968 by Robert W. Henderson and J. D. Lynch.

**Paratypes**. KU 119859–71 and KU 119911–44, topotypes collected on 10 June and 13 June 1968 by R. W. Henderson and J. D. Lynch.

**Diagnosis**. *Pristimantis versicolor* is a medium sized species, distinguished by the following combination of traits: (1) skin on dorsum shagreen with fine tubercles; skin on venter areolate; discoidal fold visible, thoracic fold prominent; dorsolateral folds absent; (2) tympanic membrane and tympanic annulus prominent, its length about 45% of the length of eye; supratympanic fold present; (3) snout subacuminate in dorsal view, rounded in profile; canthus rostralis angular, straight or slightly concave in dorsal view, angular in profile; (4) upper eyelid usually bearing several larger tubercles (trait more visible in life), about 85% IOD in females and 97% IOD in males; cranial crests absent; (5) dentigerous processes of vomers prominent, oblique, ovoid, separated medially by distance equal to twice than the width of processes; each processes bearing 3 to 4 teeth; (6) males lacking vocal sac and slits; nuptial pads present; (7) Finger I shorter than Finger II; discs on fingers broadly expanded, elliptical; circumferential grooves present; fingers bearing weak lateral fringes or none; (8) subarticular tubercles prominent, round; supernumerary palmar tubercles evident; palmar tubercle partially divided into a larger (inner) and a smaller (outer) tubercles; thenar tubercle oval, equal in size with the inner palmar tubercle; (9) ulnar tubercles low, diffuse; (10) heel with one large and several smaller tubercles (trait more visible in life); outer edge of tarsus bearing a row of small, conical tubercles; inner tarsal tubercles coalesced into a short tarsal fold; (11) inner metatarsal tubercle ovoid, about 3 to 4x round, conical (in profile), outer metatarsal tubercle; supernumerary plantar tubercles present, conical; (12) Toe V much longer than Toe III; discs on toes broadly expanded, elliptical, slightly smaller than those of fingers; circumferential grooves present; toes lacking lateral fringes; webbing absent; (13) in life, dorsum brown or green, with dark markings (chevrons, interorbital bar, canthal and supratympanic stripes); flanks with vertical bars or spotted; limbs usually with transverse bars; venter cream with black or dark brown reticulations; no markings in axilla, groin or on concealed limb surfaces; iris whitish bronze with a broad reddish median horizontal streak, that almost covers all the lower part of the iris and fine black reticulations; (14) SVL 29.4–32.2 mm in adult females (30.8 ± 1.40 SD, *n* = 3) and 22.9–25.4 mm in adult males (24.0 ± 1.08 SD, *n* = 4).

**Variation**. Morphometric variation is shown in Table 3. The “versicolor” (variegated) name is very fitting because this species displays an incredible color variation. Many individuals have a general brown coloration with green blotches and whitish markings like female MUTPL 390 (Fig 27A) or a general green coloration (MUTPL 313, Fig 27F). In some individuals the dorsum is reddish brown, with some pale green markings (MUTPL 112, Fig 27B), or have brown dorsum with orange or almost red flanks (MUTPL 112, Fig 27B). The specimen MUTPL 497 (Fig 27D) had a very special coloration with a brown broad middorsal band, bordered by dark brown dorsolateral strips, green upper part of the flanks and of the dorsal surfaces of limbs and orange with black reticulations lower part of the flanks. Many individuals have several white spots of various sizes (like MUTPL 390) but in some cases, the entire body is covered with white spots, like MUTPL 293 (Fig 27E). Almost all encountered individuals had on their flanks larger dark bars or spots. The subadults and juveniles have a coarser texture of their dorsum, flanks and of the dorsal surfaces of limbs (Fig 27D, 27E and 27F).

**Fig 28:**
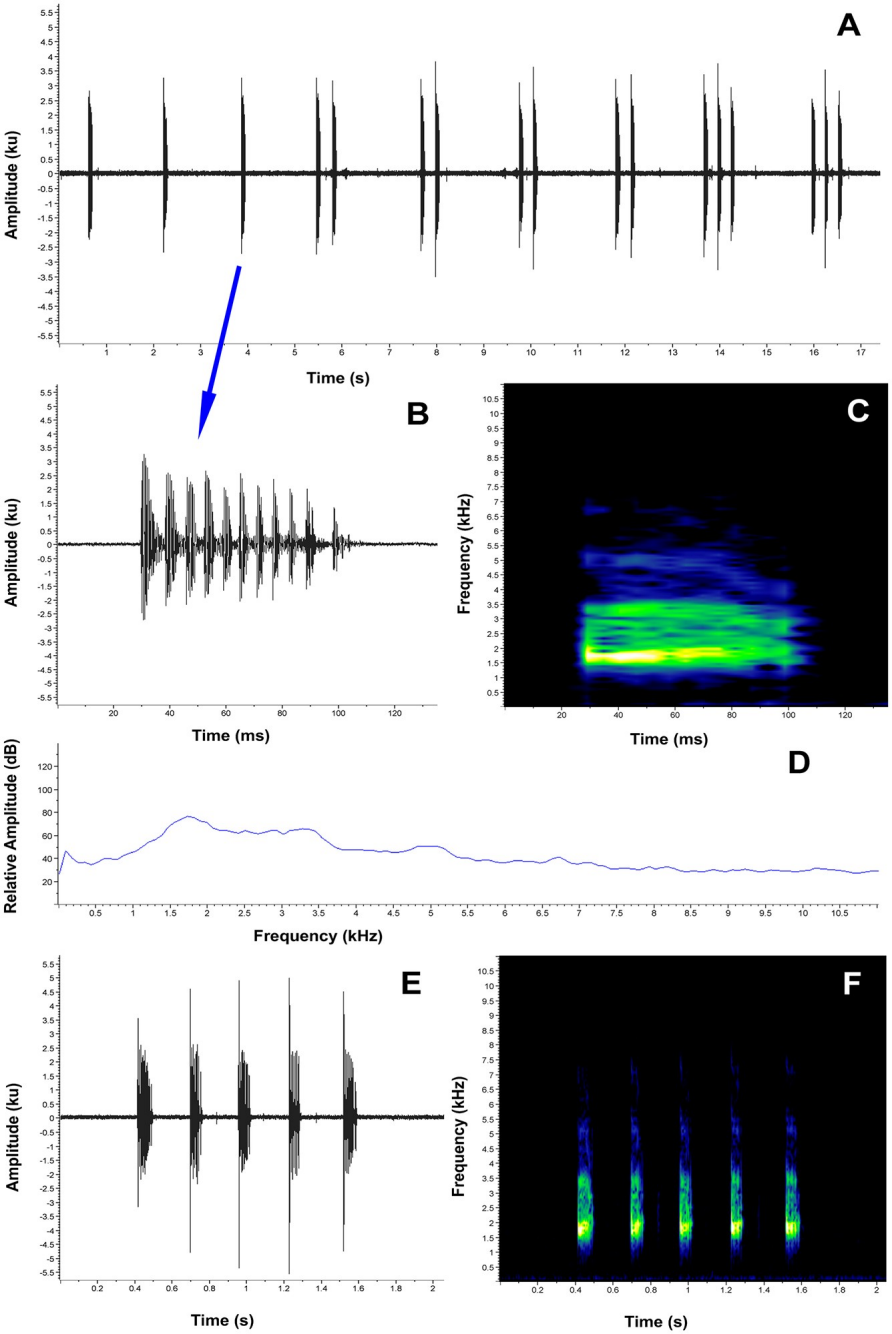
Advertisement call of *Pristimantis versicolor* (MUTPL 112, FUTPL-A 234). **A**. Oscillogram of a 9 calls section of the call series, with single, double and triple-noted calls; **B**. Oscillogram of a pulsed, single-noted call; **C**. Spectrogram of a pulsed, single-noted call; **D**. Power spectrum of a pulsed, single-noted call; **E**. Oscillogram of a 5-noted call; **F**. Spectrogram of a 5-noted call.

**Advertisement call**. The advertisement call of this species has been overlooked for a long time, probably because it resembles more a sound produced by insects. The advertisement calls of four males from Abra de Zamora were recorded in 2016 and 2018. Descriptive statistics of the acoustic variables are provided in Table 2 (the detailed information of each of the separate recordings is presented in the S4 Table). *Pristimantis versicolor* has a peculiar advertisement call, characterized by a call series composed by low squeak/chirp-like calls repeated for long periods of time. (Fig 28). The calls are composed usually by one note (pulsed sound) but sometimes (probably in case of social interactions, where there is a female or a competitive male nearby) the males can emit calls with up to 5 notes (MUTPL 112, FUTPL-A 234, Fig 28E and 28f). The barely detectable, low advertisement call might be related to the lack of vocal sac and slits. Because the males can call continuously for long periods of time, the call series duration is unknown. The calls are characterized by a mean duration of 0.084 s (SD = 0.006), a mean inter-call interval of 1.804 s (SD = 0.315) and a mean call rate of 30.3 calls/min (SD = 3.138). In the case of the double-noted calls, the call duration was on average 0.403 s (SD = 0.013), for the 5-noted calls was on average 1.211 s (SD = 0.068) and had a mean note rate of 3.48 notes/s (SD = 0.259) (S4 Table). The calls had 6 to 14 pulses/note, with a mean pulse rate of 114.58 pulses/s (SD = 21.640). The mean dominant frequency of the call was 1753.6 Hz (SD = 80.323), with a mean 90% bandwidth of 1542.3–3269.2 Hz (Table 2). The fundamental frequency is not recognizable, but sometimes 3 to 5 harmonics are visible.

**Distribution**. *Pristimantis versicolor* presence is confirmed (with DNA samples) from Reserva Cerro Plateado, Reserva Tapichalaca, Parque Nacional Podocarpus (Lagunas del Compadre), Reserva Madrigal del Podocarpus, Abra de Zamora and Ramos Urcu (at about 5 km west to San Lucas), in Loja and Zamora Chinchipe provinces (Fig 23). Very probable the animals from Peru [25] are in fact a different, currently undescribed species, taking into consideration the great distance from the type locality. The species was encountered at an altitudinal range between 2440 and 3380 m a.s.l. in montane cloud forest, evergreen upper montane forest (Fig 2C), subpáramo (Fig 2B) and shrub páramo ecosystems.

**Natural history**. This is one of the most common species from the higher part of Abra de Zamora (together with *P*. *andinognomus*). All specimens were encountered during the night, perching on the vegetation (usually at 30 cm–1.5 m above the ground) or in bromeliads. Calling males were encountered year round, but more frequently on rainy nights.

**Conservation status**. *Pristimantis versicolor* is currently categorized as Least Concern [55] following the IUCN criteria. We suggest maintaining this category due the fact that it is an abundant species (in all encountered localities), some of its populations are inside protected areas (Reserva Cerro Plateado, Reserva Tapichalaca, Parque Nacional Podocarpus and Reserva Madrigal del Podocarpus), and no major threats are predictable.

**Remarks**. Lynch [9] provides a detailed description of this species. Our diagnosis concurs with almost all of the morphological features described by the author (the only difference was the presence of a large and several small tubercles on the heel in contrast with the lack of tubercles described by Lynch); we added descriptions of the characters that are more evident in life. The diagnosis provided herein is based on 48 specimens from the original description (KU 119858–71, 119911–44), one adult female (MUTPL 390), three adult males (MUTPL 112, 294, 389) and two subadults (MUTPL 313, 497) collected from the type locality, and 8 specimens collected from the other localities.

The populations from Reserva Cerro Plateado and Reserva Tapichalaca need a careful revision because the genetic distance between specimens collected in these reserves and the ones from the type locality and nearby areas (northern populations) is around 2.0%.

Family Telmatobiidae Fitzinger, 1843

Genus ***Telmatobius*** Wiegmann, 1834

***Telmatobius cirrhacelis*** Trueb, 1979

**Common English name**. Loja Water Frog

**Common Spanish name**. Uco de Loja

**Etymology**. The specific name is derived from the Greek *kirrhos*, meaning “orange-colored, yellow, tawny”, and *kelis*, meaning “spot”, with reference to the bright orange spots that characterize this species.

**Holotype**. KU 165991, an adult female from 13 km E Loja (Abra de Zamora), 2850 m, Provincia Loja, Ecuador (latitude 03°59'S, longitude 79°08'W), taken on 8 March 1975 by William E. Duellman.

**Paratypes**. KU 165989 (male skeleton) and KU 165990 (subadult female) from 15 km E Loja, 2700 m, Provincia Zamora-Chinchipe, Ecuador (latitude 03°59'S, longitude 79°07'W).

**Remarks**. Despite numerous searching efforts carried out in the last 40 years [56] the species was not re-encountered. The last sighting of *Telmatobius cirrhacelis* dates from 1984, from Parque Nacional Podocarpus (Lagunas del Compadre), and the species is considered probably extinct.

### Other amphibian species present in Abra de Zamora

Besides the 11 species (13, with the two new species presented herein) described from Abra de Zamora, 16 other amphibian species were recorded (S5 Table).

Family Bufonidae

***Rhinella margaritifera*** (Laurenti, 1768)

(Fig 29A)

**Fig 29:**
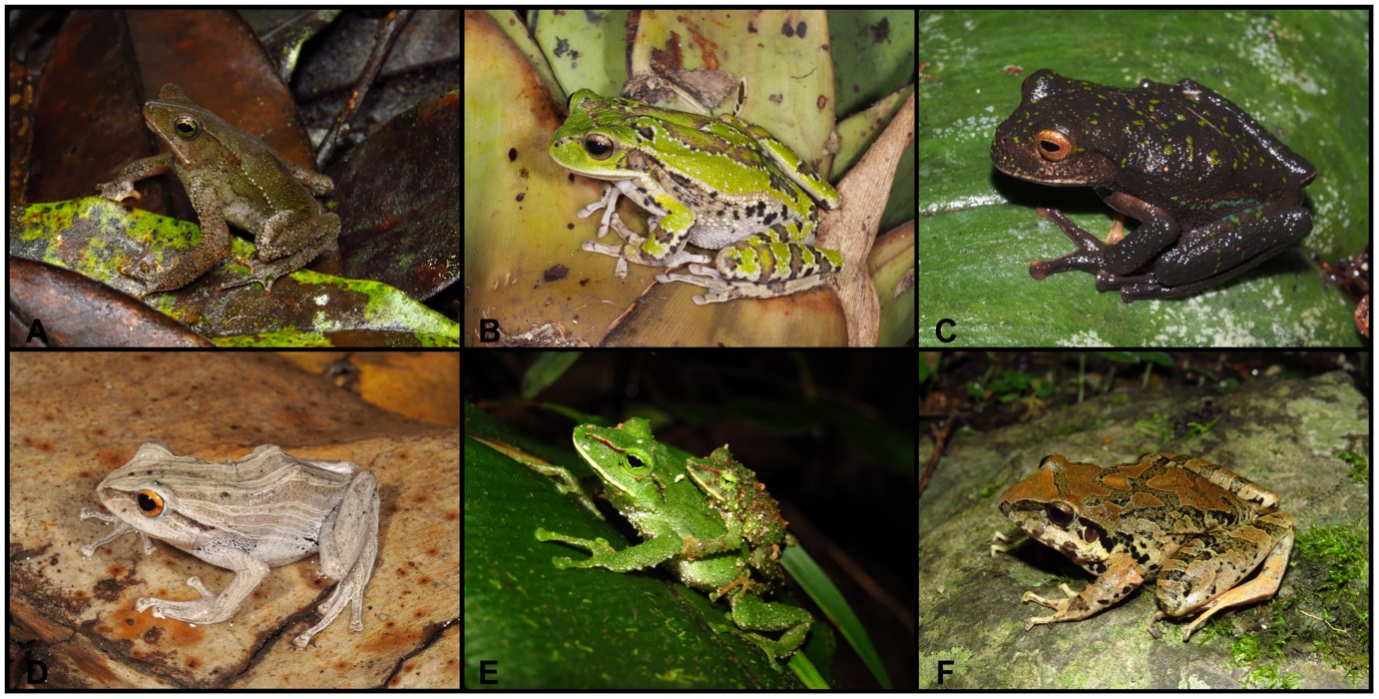
Other amphibian species present in Abra de Zamora. **A**. *Rhinella margaritifera*, from Parque Nacional Podocarpus (Bombuscaro entrance); **B**. *Gastrotheca elicioi*, from Loja city; **C**. *Gastrotheca testudínea*, from Abra de Zamora; **D**. *Pristimantis atratus*, from Abra de Zamora; **E**. *Pristimantis galdi*, from Reserva Tapichalaca; **F**. *Pristimantis lymani*, from Loja city.

**Common English name**. South American Common Toad

**Common Spanish name**. Sapo común sudamericano

**Identification**. It is easily distinguished from all other species present in Abra de Zamora by the presence of developed canthal, antorbital, and supra-orbital crests (larger in females), small, triangular parotoid glands, and granulose skin on dorsum with many small tubercles [57].

**Remarks**. This is an uncommon species in Abra de Zamora. Can be encountered on the eastern slope, in evergreen lower montane forest (Fig 2D), bellow 2000 m, near the El Tambo sector. Its precise identity has yet to be confirmed, as it is part of a species complex.

Family Hemiphractidae

***Gastrotheca elicioi*** Carvajal-Endara, Coloma, Morales-Mite, Guayasamin, Székely, and Duellman, 2019

(Fig 29B)

**Common English name**. Elicio’s Marsupial Frog

**Common Spanish name**. Rana marsupial de Elicio

**Identification**. For now, it is the only *Gastrotheca* species identified on the western slope of Abra de Zamora. Despite our intensive searches, we did not find *G*. *lojana*, the other species reported from the area. Based on morphological characters, *G*. *elicioi* can be difficult to distinguish from *G*. *lojana*, the main differences being the color pattern (characters of *G*. *lojana* in parenthesis): distinct dark canthal stripes present (absent), groin and anterior and posterior surfaces of the thighs slightly mottled (heavily mottled), and the dark bars on the limbs, when present, are shorter, thinner and less defined than in *G*. *lojana* [58]. However, the unique advertisement call of *G*. *elicioi* (the long, pulsed notes are produced after the short ones, whereas the other Southern Ecuadorian *Gastrotheca* species tend to produce the long, pulsed notes before the short ones) make it very easy to detect and identify.

**Remarks**. This is a common species in Abra de Zamora and nearby Loja city. Its distribution is restricted to the western slope, close to the city, in evergreen upper montane forest bellow 2600 m. The individuals are usually difficult to encounter, as they are very well camouflaged in the vegetation; however, the males’ characteristic call can be heard frequently, especially on rainy days.

***Gastrotheca testudinea*** (Jiménez de la Espada, 1870)

(Fig 29C)

**Common English name**. Espada’s Marsupial Frog

**Common Spanish name**. Rana marsupial de Jimenez de la Espada

**Identification**. For now, it is the only *Gastrotheca* species recorded on the eastern slope of Abra de Zamora. It is easily distinguished from all the other species present in Abra de Zamora by its large size, robust body, wide head, flat head top, and characteristic advertisement call [8].

**Remarks**. This is an uncommon species in Abra de Zamora. It can be encountered on the eastern slope in evergreen lower montane forest (Fig 2D) bellow 2200 m, near the El Tambo sector. Individuals are difficult to find, especially in dense vegetation, but the males’ characteristic call can be heard frequently, especially on rainy days.

Family Strabomantidae

***Pristimantis atratus*** (Lynch, 1979)

(Fig 29D)

**Common English name**. Santiago Robber Frog

**Common Spanish name**. Cutín del Suro Rancho

**Identification**. This small sized species is easily distinguished from all the other *Pristimantis* species present in Abra de Zamora by its whitish, yellowish coloration with pale brown strips, yellowish orange iris, skin on dorsum with low ridges, and black and white (sometimes yellow or orange) markings in axilla, groin and concealed limb surfaces [9].

**Remarks**. This is an uncommon species in Abra de Zamora. Can be encountered on the eastern slope in evergreen upper montane forest (Fig 2C), between 2700 and 2500 m.

***Pristimantis galdi*** Jiménez de la Espada, 1870

(Fig 29E)

**Common English name**. Espada’s Robber Frog

**Common Spanish name**. Cutín verde amazónico

**Identification**. This small sized species is easily distinguished from all other *Pristimantis* species present in Abra de Zamora by the bright green dorsum, green iris, acuminate snout, flared lips, serrated cranial crests (especially in females), a conical tubercle on the upper eyelid and greatly expanded, truncate discs on Fingers II–IV [25].

**Remarks**. This is an uncommon species in Abra de Zamora. Can be encountered on the eastern slope in evergreen lower montane forest (Fig 2D), around 1900 m, near the El Tambo sector.

***Pristimantis lymani*** (Barbour and Noble, 1920)

(Fig 29F)

**Common English name**. Lyman’s Robber Frog

**Common Spanish name**. Cutín de Lyman

**Identification**. This species is easily distinguished from all the other *Pristimantis* species present in Abra de Zamora by its large size, prominent tympanum, smooth skin on the venter, reddish brown dorsum, canthal and postorbital dark stripes, chevrons on the back, and transverse bars on the limbs [25]. It is the only member of the *Pristimantis conspicillatus* species group, and the only terrestrial *Pristimantis* species from the area.

**Remarks**. This is a common species in Abra de Zamora and the most common species in the nearby Loja city. Can be encountered on the western slope in evergreen upper montane forest bellow 2600 m, and in the pasturelands neighboring the city.

Additionally, 10 potential new species were encountered in Abra de Zamora:

- from the western slope, in the vicinity of Loja city, *Noblela* aff. *heyeri* and two *Pristimantis* species (*P*. aff. *cajamarcensis* and *P*. aff. *torresi*);
- from the eastern slope, heading to El Tambo, an undescribed species of salamander (*Bolitoglossa* sp.), *Atelopus* aff. *halihelos*, two undescribed species of glass frogs (*Centrolene* sp.), *Noblela* aff. *heyeri*, and three undescribed species of *Pristimantis*.

Unfortunately, from the 29 species registered (belonging to six families, Plethodontidae, Bufonidae, Centrolenidae, Hemiphractidae, Strabomantidae and Telmatobiidae), one (*A*. aff. *halihelos*) was not encountered in the past ten years and three others (*A*. *podocarpus*, *G*. *psychrophila*, and *T*. *cirrhacelis*) were never seen again for over twenty years.

## Discussion

### Systematics

Already in 1979, Lynch [9] observed the similarity between *P*. *vidua* and *P*. *orestes* and hypothesized a close relationship; this was later recognized as a distinct assemblage in 1997 by Lynch and Duellman [59], composed by three Ecuadorian species (*P*. *orestes*, *P*. *simonbolivari* and *P*. *vidua*). We now have the phylogenetic confirmation that indeed *P*. *vidua* is a member of the resurrected *P*. *orestes* group (sensu [29]), being closely related to the species of the *P*. *orestes* subgroup: *P*. *andinognomus*, *P*. *orestes*, *P*. *cajanuma* and an undescribed species (Fig 7).

The situation was different for *P*. *colodactylus*, which, due to its particular morphological features, was included in a different group. In the original description [9], Lynch hypothesized that *P*. *colodactylus* may be related to *P*. *proserpens* and *P*. *celator*, and in 1980 Lynch and Duellman [59] assigned it to the *P*. *celator* assemblage of the *P*. *unistrigatus* species group. The membership to the *P*. *unistrigatus* species group was maintained by Duellman and Lehr [25] and Hedges et al. [41]. With the description of *P*. *muranunka*, Brito et al. [29] recognized the morphological similarities of this species with *P*. *colodactylus* (and also with *P*. *proserpens*, *P*. *paquishae*, and *P*. *tinajillas*). In 2019, Urgilles et al. [16] recognized *P*. *colodactylus* (together with *P*. *vidua* and *P*. *tinajillas*) as a potential member of the *P*. *orestes* group. We provide genetic evidence that *P*. *colodactylus* is a member of the *P*. *orestes* group, which, together with *P*. *muranunka*, *P*. *matildae* and an undescribed species, forms a subgroup of closely related, morphologically distinct, bromeliad specialist species (Fig 7).

In the original description of the species Lynch [9] observed several similarities between *P*. *balionotus* and *P*. *percultus* and “very tentatively” associated them with the *balionotus*-*riveti*-*ruidus* series. In 1980, Lynch and Duellman [59] assigned both species to the *P*. *unistrigatus* species group, although to two different assemblages: *P*. *balionotus* to the *P*. *unistrigatus* assembly and *P*. *percultus* to the *P*. *glandulosus* assembly. Hedges et al. [41] assigned both species to the *P*. *unistrigatus* species group. In 2019, Páez and Ron [30] assigned *P*. *balionotus* and *P*. *percultus*, based on their similar morphology, to the *P*. *phoxocephalus* species group of the newly proposed *Huicundomantis* subgenus. Our analysis support their assumption and show that both species are members of the *P*. *phoxocephalus* species group. In our phylogram, *P*. *balionotus* kinship to the other members of the group remains non-resolved (with suggested relationship to *P*. *hampatusami* and an undescribed species), but *P*. *percultus* is part of a strongly supported subgroup composed of *P*. *chomskyi*, *P*. *andinogigas*, *P*. *atratus* and *P*. *multicolor* (Fig 21).

In 2019 Yánez-Muñoz et al. [60] described a new, “giant”, *Pristimantis* species, *P*. *andinogigas*, from the Cajanuma sector of Parque Nacional Podocarpus. As their description was not based on a phylogenetic analysis, the authors refrained from assigning it to any species-group although they noted its morphological similarity to members of the former *P*. *orcesi* group. In our analysis, *P*. *andinogigas* is closely related to *P*. *chomskyi* and to other three species (*P*. *percultus*, *P*. *atratus* and *P*. *multicolor*) of a strongly supported subgroup of the *P*. *phoxocephalus* species group. However, a careful revision is needed for *P*. *andinogigas* and *P*. *chomskyi* because the genetic distance between the two species is around 2% and it seems that the description of *P*. *chomskyi* was based on subadult specimens, as the individuals from the type locality and nearby areas are in fact large, morphologically very similar animals to *P*. *andinogigas* (pers. obs.).

### Conservation remarks

Even though most of Abra de Zamora is well preserved and about 50% of its total area is protected, being inside Parque Nacional Podocarpus, the amphibians of this area face several threats. Among the main threats we can mention are the loss and degradation of habitats, due to cattle farming, and the introduction of exotic species (Rainbow Trout, *Oncorhynchus mykiss* and Bullfrog, *Lithobates catesbeianus*), species that have already been proven to have a direct negative effect on Andean amphibian populations [61]. On the other hand, forest fires are also a potential threat, since they occur frequently in nearby sectors intended for agriculture or livestock.

As for the infectious diseases that are affecting the Ecuadorian amphibian populations, we have limited information from the study area. The presence of chytrid fungus (*Batrachochytrium dendrobatidis*) is documented from the Andes of central and northern Ecuador [62, 63]. A recent survey conducted by researchers from University of Central Florida, yielded negative results for samples collected from Abra de Zamora, (Urgiles et al., pers. com.). However, it is possible that the decline of several species in Abra de Zamora could be caused by the chytrid fungus [62].

To assure an adequate protection of the areas that currently are not included in Parque Nacional Podocarpus, we are working on a proposal, in collaboration with the local governments and Ecuadorian Ministry of Environment. Additionally, by the end of 2020, the Action Plan for the conservation of the amphibians of Abra de Zamora will be available, generated by the EcoSs Lab group of Universidad Técnica Particular de Loja and supported by funding from the Critical Ecosystem Partnership Fund (CEPF).

## Supporting information

S1 Appendix Additional specimens examined. (DOCX)

S1 Table Primers used for PCR amplification of *12S* rRNA, *16S* rRNA, and *RAG1* nDNA. (DOCX)

S2 Table Voucher, GenBank accession numbers and locality for the specimens used in the phylogenetic analysis. (DOCX)

S3 Table Uncorrected pairwise distances (%), for the mitochondrial gene *16S* fragment, for *Lynchius*, *Pristimantis orestes* group and *Huicundomantis* subgenus of *Pristimantis*. (XLSX)

S4 Table Information regarding the call recordings and the bioacoustic measurements for each of the recorded males. Values are given as average ± SD (range) and *n* = sample size.
(XLSX)

S5 Table Amphibian species recorded in Abra de Zamora. (XLSX)
