# Supplementary material for: Fifty years after: A taxonomic revision of the amphibian species from the Ecuadorian biodiversity hotspot Abra de Zamora, with description of two new Pristimantis species
Source: PLoS One. 2020 Sep 10;15(9):e0238306. doi: 10.1371/journal.pone.0238306 (PMC7482940; doi:10.1371/journal.pone.0238306)
Supplement: S1 Appendix — (DOCX) [file pone.0238306.s001.docx]

**S1 Appendix. Additional specimens examined.**

*Pristimantis andinogigas.* ECUADOR: LOJA PROVINCE, Parque Nacional Podocarpus – Cajanuma (MUTPL 359).

*Pristimantis andinognomus*. ECUADOR: ZAMORA CHINCHIPE PROVINCE, Palanda, Valladolid, Reserva Tapichalaca (QCAZ 26964, 26965, 27033, 29248, 35374, 35375, 35377, 35378, 45614, 45621, 45622, 45623, 45656, 45659, 45661, 45662).

*Pristimantis bambu*. ECUADOR: CAÑAR PROVINCE, La Libertad, Reserva Mazar (MZUTI 3373, 3378, 3420; QCAZ 68126, 68127, 68131, 68133–68143, 68145, 68147, 68151, 68154, 68161, 68329, 68335, 68342, 68374, 68375, 68402); ZAMORA CHINCHIPE PROVINCE, Yacuambi, Imbana (QCAZ 54356).

*Pristimantis gloria.* ECUADOR: LOJA PROVINCE, Vía Urdaneta-Tutupali (MUTPL 222–238, 250); ZAMORA CHINCHIPE PROVINCE, Belen, Bosque Protector Shincata (MUTPL 801).

*Pristimantis mazar*. ECUADOR: CAÑAR PROVINCE, El Tambo (QCAZ 2569); La Libertad, Reserva Mazar (MZUTI 3418, 3488; QCAZ 27493, 27503–27505, 27507, 27508, 27511, 27514, 27519, 27553–27555, 27560, 27563, 27565, 32619, 49750, 49764, 68334, 68344, 68353, 68368, 68383, 68386, 68387, 68391, 68392, 68394–68397, 68405, 68409, 68413).

*Pristimantis muranunka*. ECUADOR: ZAMORA CHINCHIPE PROVINCE, Cerro Plateado (MUTPL 652); Reserva Cerro Plateado (MUTPL 605, 606, 643)

*Pristimantis orestes*. ECUADOR: AZUAY PROVINCE, Vía Sigsig-Gualaquiza (QCAZ 40783); LOJA PROVINCE, Vía Urdaneta-Tutupali (MUTPL 242, 248, 249).

*Pristimantis* aff. *orestes*. ECUADOR: AZUAY PROVINCE, Cooperativa Bellarica (QCAZ 52584); El Guillan, Hacienda de la Universidad del Azuay (QCAZ 59019–59021, 61872–61874, 61877); En el camino entre Gualaceo y Plan de Milagro (QCAZ 27056); Matanga (QCAZ 29134); Vía Susudel-Cuenca, a aproximadamente 1 km de Susudel (MZUTI 706); Yumate, Shoupshe (QCAZ 46991); 8 km sur de La Paz en Panamericana (QCAZ 45935); CAÑAR PROVINCE, La Libertad, Reserva Mazar (QCAZ 27482, 27498, 27513, 27559, 27563, 27606–27608, 28136; 32611, 32613–32615, 49753, 68129, 68358); CHIMBORAZO PROVINCE, Río Atillo (QCAZ 3341); EL ORO PROVINCE, Chillacocha (QCAZ 45093, 45096); LOJA PROVINCE, Bosque Protector Washapamba (QCAZ 40804; MUTPL 136–142, 172); Parque Nacional Podocarpus (QCAZ 4974); Saraguro (58567–58570); MORONA SANTIAGO PROVINCE, Límite Provincial entre Azuay y Morona Santiago, vía Sigsig-Cutchil-Oriente, 25 km este de Sigsig (QCAZ 3443–3446).

*Pristimantis simonbolivari*. ECUADOR: AZUAY PROVINCE, Cuenca, Baños (QCAZ 37665–37670); BOLÍVAR PROVINCE, Bosque Protector Cashca Totoras (QCAZ 932, 939, 942, 12932, 13755, 13759, 13763, 16819, 16823, 16830, 16832, 16925, 25107, 25108, 25113, 25120, 25121, 25123, 30893, 35747-35749, 36669, 37665–37670, 42591, 42594, 42598, 49361); 12 km este de Guaranda, carretera Guaranda-Riobamba (QCAZ 1497).
