# Supplementary material for: Fifty years after: A taxonomic revision of the amphibian species from the Ecuadorian biodiversity hotspot Abra de Zamora, with description of two new Pristimantis species
Source: PLoS One. 2020 Sep 10;15(9):e0238306. doi: 10.1371/journal.pone.0238306 (PMC7482940; doi:10.1371/journal.pone.0238306)
Supplement: S1 Table — (DOCX) [file pone.0238306.s002.docx]

**S1 Table.** **List of primers used for PCR amplification of *12S* rRNA, *16S* rRNA, and *RAG1* nDNA.**

| **Locus** | **Primer name** | **Direction** | **Sequence** | **Annealing Temperature** | **Reference** |
| --- | --- | --- | --- | --- | --- |
| *12S* rRNA | 12L29E | forward | AAAGCRTAGCACTGAAAATGCTAAGA | 55° C | Heinicke et al., 2007 |
|  | 12H10 | reverse | CACYTTCCRGTRCRYTTACCRTGTTACGACTT |  | Heinicke et al., 2007 |
| *12S* rRNA | 12L29E | forward | AAAGCRTAGCACTGAAAATGCTAAGA | 55° C | Heinicke et al., 2007 |
|  | 12H46E | reverse | GCTGCACYTTGACCTGACGT |  | Heinicke et al., 2007 |
| *16S* rRNA | 16SC | forward | GTRGGCCTAAAAGCAGCCAC | 55° C | Darst & Cannatella, 2004 |
|  | 16SD | reverse | CTCCGGTCTGAACTCAGATCACGTAG |  | Darst & Cannatella, 2004 |
| *16S* rRNA | 16Sar-L | forward | CGCCTGTTTATCAAAAACAT | 55° C | Palumbi et al., 1991 |
|  | 16Sbr-H | reverse | CCGGTCTGAACTCAGATCACGT |  | Palumbi et al., 1991 |
| *RAG1* nDNA | R182 | forward | GCCATAACTGCTGGAGCATYAT | 52° C | Cannatella, D. pers. comm. |
|  | R270 | reverse | AGYAGATGTTGCCTGGGTCTTC |  | Cannatella, D. pers. comm. |

Darst, C. R., and D. C. Cannatella. (2004). Novel relationships among hyloid frogs inferred from 12S and 16S mitochondrial DNA sequences. Molecular Phylogenetics and Evolution 31: 462–475.

Heinicke, M. P., Duellman, W. E., & Hedges, S. B. (2007). Major Caribbean and Central American frog faunas originated by ancient oceanic dispersal. *Proceedings of the National Academy of Sciences*, *104*(24), 10092-10097.

Palumbi, S., Martin, A., Romano, S., McMillan, W. O., Stice, L., Grabowski, G., & MacMillan, W. O. (2002). The simple fool's guide to PCR, version 2.0. Honolulu, University of Hawaii.
