# Supplementary material for: Fifty years after: A taxonomic revision of the amphibian species from the Ecuadorian biodiversity hotspot Abra de Zamora, with description of two new Pristimantis species
Source: PLoS One. 2020 Sep 10;15(9):e0238306. doi: 10.1371/journal.pone.0238306 (PMC7482940; doi:10.1371/journal.pone.0238306)
Supplement: S2 Table — (DOCX) [file pone.0238306.s003.docx]

**S2 Table.** **Voucher, GenBank accession numbers and locality for the specimens used in the phylogenetic analysis.**

| **Species** | **Voucher number** | **GenBank accession no.** | | | **Locality** |
| --- | --- | --- | --- | --- | --- |
|  |  | ***12S*** | ***16S*** | ***RAG1*** |  |
| *Lynchius flavomaculatus*? | KU218210 | EU186667 | EU186667 | EU186745 | Ecuador: Loja, 19.4 km S Yangana |
| *Lynchius flavomaculatus* | MUTPL342 | MT764337 | MT742799 | - | Ecuador: Loja, Parque Nacional Podocarpus, Cajanuma |
| *Lynchius megacephalus* | QCAZ63882 | - | MK423938 | - | Ecuador: Morona Santiago, Tinajillas |
| *Lynchius nebulanastes* | KU181408 | EU186704 | EU186704 | - | Peru: Piura, El Tambo, 31.5 km E Canchaque |
| *Lynchius oblitus* | MHNC8614 | KX470776 | KX470783 | KX470792 | Peru: Cajamarca, San Ignacio, Santuario Nacional Tabaconas Namballe, Laguna Victoria |
| *Lynchius oblitus* | MHNC8652 | KX470775 | KX470782 | KX470791 | Peru: Cajamarca, San Ignacio, Santuario Nacional Tabaconas Namballe, Quebrada del Vino |
| *Lynchius oblitus* | MHNC8677 | KX470779 | KX470786 | KX470795 | Peru: Cajamarca, San Ignacio, Santuario Nacional Tabaconas Namballe, Quebrada del Vino |
| *Lynchius parkeri* | KU181307 | EU186705 | EU186705 | - | Peru: Piura, 31 km SW Huancabamba |
| *Lynchius parkeri* | QCAZ31466 | MK423935 | - | MK423933 | Ecuador: Loja, Parque Nacional Yacuri, Laguna de Negra de Jimbura |
| *Lynchius parkeri* | QCAZ61015 | MK423936 | MK423937 | MK423934 | Ecuador: Loja, Parque Nacional Yacuri, Vía  a Jimbura |
| *Lynchius simmonsi* | MUTPL504 | MT764338 | - | MT810304 | Ecuador: Zamora Chinchipe, Alto Nangaritza, Las Orquídeas, Tepuy |
| *Lynchius simmonsi* | QCAZ41639 | JF809940 | JF810004 | JF809915 | Ecuador: Zamora Chinchipe, Alto Miazi, Cordillera del Condor |
| *Lynchius tabaconas* | MHNC8637 | KX470773 | KX470780 | KX470789 | Peru: Cajamarca, San Ignacio, Santuario Nacional Tabaconas Namballe, Quebrada del Vino |
| *Lynchius tabaconas* | MHNC8650 | KX470774 | KX470781 | KX470790 | Peru: Cajamarca, San Ignacio, Santuario Nacional Tabaconas Namballe, Quebrada del Vino |
| *Pristimantis andinogigas* | MUTPL359 | MT764339 | MT756022 | MT810305 | Ecuador: Loja, Parque Nacional Podocarpus, Cajanuma |
| *Pristimantis andinognomus* | QCAZ45534 | - | KY967669 | KY967688 | Ecuador: Loja, Parque Nacional Podocarpus, guardianía Cajanuma |
| *Pristimantis andinognomus* | QCAZ45661 | - | KY967671 | KY967690 | Ecuador: Zamora Chinchipe, Reserva Tapichalaca |
| *Pristimantis atillo* | QCAZ42488 | - | MK881440 | MK881340 | Ecuador: Morona Santiago, Parque Nacional Sangay, Lagunas de Atillo |
| *Pristimantis atillo* | QCAZ42498 | - | MK881444 | MK881344 | Ecuador: Morona Santiago, Parque Nacional Sangay |
| *Pristimantis atratus* | QCAZ45580 | - | MK881471 | MK881364 | Ecuador: Loja, San Sebastián, Cajanuma |
| *Pristimantis atratus* | QCAZ45645 | - | MK881473 | MK881366 | Ecuador: Loja, Parque Nacional Podocarpus, Cajanuma |
| *Pristimantis balionotus* | MUTPL180 | MT778069 | MT756023 | MT810306 | Ecuador: Loja, Reserva Madrigal del Podocarpus |
| *Pristimantis balionotus* | MUTPL391 | MT778070 | MT756024 | MT810307 | Ecuador: Loja, Abra de Zamora |
| *Pristimantis balionotus* | MUTPL392 | MT778071 | MT756025 | MT810308 | Ecuador: Loja, Abra de Zamora |
| *Pristimantis bambu* | QCAZ46743 | - | KY967674 | KY967692 | Ecuador: Cañar, Reserva Mazar |
| *Pristimantis bambu* | QCAZ46744 | - | KY967659 | KY967693 | Ecuador: Cañar, Reserva Mazar |
| *Pristimantis cajanuma* | MUTPL344 | MK993331 | MK604535 | - | Ecuador: Loja, Parque Nacional Podocarpus, Cajanuma |
| *Pristimantis cajanuma* | MUTPL345 | MK993331 | MK604535 | MK602184 | Ecuador: Loja, Parque Nacional Podocarpus, Cajanuma |
| *Pristimantis chomskyi* | QCAZ45666 | - | MK881476 | MK881369 | Ecuador: Zamora Chinchipe, Reserva Tapichalaca |
| *Pristimantis chomskyi* | QCAZ45669 | - | MK881477 | MK881370 | Ecuador: Zamora Chinchipe, Reserva Tapichalaca |
| *Pristimantis colodactylus* | MUTPL311 | MT764340 | MT764804 | MT810309 | Ecuador: Loja, Abra de Zamora |
| *Pristimantis colodactylus* | MUTPL388 | MT778072 | MT762200 | MT810310 | Ecuador: Loja, Abra de Zamora |
| *Pristimantis cryptomelas* | MUTPL135 | MT778073 | MT756026 | MT810311 | Ecuador: Loja, Abra de Zamora |
| *Pristimantis cryptomelas* | MUTPL167 | MT778074 | MT756027 | MT810312 | Ecuador: Loja, Bosque Protector Washapamba |
| *Pristimantis cryptomelas* | MUTPL168 | MT778075 | MT756028 | MT810313 | Ecuador: Loja, Bosque Protector Washapamba |
| *Pristimantis cryptomelas* | MUTPL380 | MT778076 | MT756029 | MT810314 | Ecuador: Loja, Loja, Huacapamba |
| *Pristimantis cryptomelas* | MUTPL470 | MT778077 | MT756030 | MT810315 | Ecuador: Loja, Abra de Zamora |
| *Pristimantis cryptomelas* | MUTPL493 | MT778078 | MT756031 | MT810316 | Ecuador: Loja, Parque Nacional Podocarpus, Cajanuma |
| *Pristimantis cryptomelas* | QCAZ45612 | - | MK881472 | MK881365 | Ecuador: Zamora Chinchipe, Reserva Tapichalaca |
| *Pristimantis gagliardoi* | QCAZ42575 | - | MK881456 | MK881355 | Ecuador: Morona Santiago, Parque Nacional Sangay, Ranger Station, Tinguichaca river |
| *Pristimantis gagliardoi* | QCAZ46738 | - | MK881480 | MK881372 | Ecuador: Cañar, Reserva Mazar |
| *Pristimantis gloria* | KU218035 | EF493348 | EF493348 | - | Ecuador: Azuay, 8.1 km W Morona Santiago border, Gualaceo-Limón road |
| *Pristimantis gloria* | MUTPL223 | MT778079 | MT756032 | MT810317 | Ecuador: Loja, 21 km E Urdaneta |
| *Pristimantis gloria* | MUTPL250 | MT778080 | MT756033 | MT810318 | Ecuador: Loja, 10 km E Urdaneta |
| *Pristimantis gloria* | QCAZ16448 | - | MK881402 | MK881316 | Ecuador: Azuay, Gualaceo-Macas road |
| *Pristimantis gloria* | QCAZ31455 | - | MK881415 | - | Ecuador: Loja, Fierro Urco |
| *Pristimantis hampatusami* | QCAZ58042 | - | MK881504 | MK881387 | Ecuador: El Oro, Reserva Buenaventura |
| *Pristimantis hampatusami* | QCAZ58044 | - | KX525478 | KX525472 | Ecuador: El Oro, Reserva Buenaventura |
| *Pristimantis jimenezi* | QCAZ45178 | - | MK881468 | MK881362 | Ecuador: Azuay, San Antonio, Parque Nacional Cajas border |
| *Pristimantis jimenezi* | QCAZ46978 | - | MK881482 | MK881374 | Ecuador: Azuay, Molleturo, Zadracay river |
| *Pristimantis lutzae* | QCAZ32785 | - | MK881421 | MK881326 | Ecuador: Azuay, Bosque Protector Yanuncay-Irquis, Páramo de Quimsacocha |
| *Pristimantis lutzae* | QCAZ53728 | - | MK881495 | - | Ecuador: Azuay, Parque Nacional Cajas, El Capo, Laguna Toreadora |
| *Pristimantis matildae* | MUTPL360 | MT778081 | MT762201 | MT810319 | Ecuador: Loja, Parque Nacional Podocarpus, Cajanuma |
| *Pristimantis matildae* | MUTPL362 | MT778082 | MT764805 | MT810320 | Ecuador: Loja, Parque Nacional Podocarpus, Cajanuma |
| *Pristimantis matildae* | MUTPL366 | MT778083 | MT764806 | MT810321 | Ecuador: Loja, Abra de Zamora |
| *Pristimantis matildae* | MUTPL394 | MT778084 | MT762202 | MT810322 | Ecuador: Loja, Abra de Zamora |
| *Pristimantis mazar* | QCAZ27559 | - | KY967664 | KY967683 | Ecuador: Cañar, Reserva Mazar, La Libertad |
| *Pristimantis mazar* | QCAZ27572 | JF906315 | KY967666 | KY967685 | Ecuador: Cañar, Reserva Mazar, La Libertad |
| *Pristimantis multicolor* | QCAZ47213 | - | MK881488 | - | Ecuador: Loja, Parque Nacional Yacuri, Laguna Negra |
| *Pristimantis multicolor* | QCAZ47214 | - | MK881489 | - | Ecuador: Loja, Parque Nacional Yacuri, Laguna Negra |
| *Pristimantis muranunka* | MEPN14722 | - | KY967660 | KY967679 | Ecuador: Zamora Chinchipe, Reserva Cerro Plateado |
| *Pristimantis muranunka* | MEPN14737 | - | KY967661 | KY967680 | Ecuador: Zamora Chinchipe, Reserva Cerro Plateado |
| *Pristimantis muranunka* | MUTPL605 | MT778085 | MT764807 | MT810323 | Ecuador: Zamora Chinchipe, Reserva Cerro Plateado |
| *Pristimantis muranunka* | MUTPL652 | MT778086 | MT764808 | MT810324 | Ecuador: Zamora Chinchipe, Reserva Cerro Plateado |
| *Pristimantis muscosus* | QCAZ54857 | - | MK881501 | MK881386 | Ecuador: Zamora Chinchipe, Reserva Tapichalaca |
| *Pristimantis nangaritza* | QCAZ41710 | - | MK881436 | MK881336 | Ecuador: Zamora Chinchipe, Alto Nangaritza PF, Las Orquídeas, Tepuy forest |
| *Pristimantis orestes* | KU218257 | EF493388 | EF493388 | - | Ecuador: Azuay, 7 km E Sigsig |
| *Pristimantis orestes* | MUTPL242 | MT778087 | MK604538 | MK602185 | Ecuador: Loja, 11 km NE Urdaneta |
| *Pristimantis orestes* | MUTPL248 | MK993330 | MK604539 | MK602186 | Ecuador: Loja, 11 km NE Urdaneta |
| *Pristimantis percultus* | MUTPL810 | MT778088 | MT756034 | MT810325 | Ecuador: Loja, Parque Nacional Podocarpus, Cajanuma |
| *Pristimantis percultus* | MUTPL812 | MT778089 | MT756035 | MT810326 | Ecuador: Loja, Parque Nacional Podocarpus, Cajanuma |
| *Pristimantis philipi* | KU217863 | EF493672 | EF493672 | - | Ecuador: Azuay, 4 km W Laguna Toreadora, nearby Parque Nacional Cajas |
| *Pristimantis philipi* | QCAZ37537 | - | MK881426 | MK881331 | Ecuador: Azuay, Parque Nacional Cajas |
| *Pristimantis phoxocephalus* | QCAZ58463 | - | MK881507 | MK881390 | Ecuador: Cotopaxi, Pilaló surroundings |
| *Pristimantis quintanai* | MZUA.AN.1881 | MK993335 | MK604541 | MK602188 | Ecuador: Cañar, Comunidad Guangras |
| *Pristimantis quintanai* | MZUA.AN.1900 | MK993336 | MK604544 | MK602189 | Ecuador: Cañar, Llavircay |
| *Pristimantis samaniegoi* | MUTPL356 | MT778090 | MT764809 | MT810327 | Ecuador: Loja, Parque Nacional Podocarpus, Cajanuma |
| *Pristimantis samaniegoi* | MUTPL357 | MT778091 | MT764810 | MT810328 | Ecuador: Loja, Parque Nacional Podocarpus, Cajanuma |
| *Pristimantis samaniegoi* | MUTPL365 | MT778092 | MT764811 | MT810329 | Ecuador: Loja, Abra de Zamora |
| *Pristimantis saturninoi* | DHMECN12214 | MK993328 | MK604532 | - | Ecuador: Morona Santiago, Parque Nacional Sangay |
| *Pristimantis saturninoi* | DHMECN12232 | MK993327 | MK604533 | - | Ecuador: Morona Santiago, Parque Nacional Sangay |
| *Pristimantis simonbolivari* | KU218254 | EF493671 | EF493671 | - | Ecuador: Bolívar, Bosque Protector Cashca Totoras |
| *Pristimantis simonbolivari* | QCAZ56567 | - | KY967676 | KY967695 | Ecuador: Bolívar, Bosque Protector Cashca Totoras |
| *Pristimantis spinosus* | KU218052 | EF493673 | EF493673 | - | Ecuador: Morona Santiago, 10.6 km W Plan de Milagro |
| *Pristimantis teslai* | QCAZ46213 | - | MK881478 | - | Ecuador: Tungurahua, Llanganatillo, Parque Nacional Llanganates border |
| *Pristimantis tiktik* | MUTPL239 | MH668274 | MH668275 | MH708575 | Ecuador: Loja, 21 km E Urdaneta |
| *Pristimantis tiktik* | MUTPL247 | MH668161 | MH668276 | MH708576 | Ecuador: Loja, 14 km E Urdaneta |
| *Pristimantis tinguichaca* | QCAZ31945 | - | MK881418 | MK881323 | Ecuador: Morona Santiago, Parque Nacional Sangay, San Vicente |
| *Pristimantis tinguichaca* | QCAZ40582 | - | MK881433 | MK881334 | Ecuador: Morona Santiago, Parque Nacional Sangay, Lagunas de Atillo |
| *Pristimantis torresi* | QCAZ47342 | - | MK881490 | - | Ecuador: Loja, Guachanamá, military antennas |
| *Pristimantis torresi* | QCAZ47397 | - | MK881492 | MK881380 | Ecuador: Loja, Celica-Alamor road |
| *Pristimantis totoroi* | KU218025 | EF493349 | EF493349 | - | Ecuador: Chimborazo, 70 km W Riobamba via Pallatanga |
| *Pristimantis totoroi* | QCAZ25105 | - | MK881406 | MK881319 | Ecuador: Bolívar, Bosque Protector Cashca Totoras |
| *Pristimantis totoroi* | QCAZ58425 | - | MK881505 | MK881388 | Ecuador: Cotopaxi, Pilaló surroundings |
| *Pristimantis verrucolatus* | QCAZ46982 | - | MK881483 | MK881375 | Ecuador: Azuay, Yumate, Shoupshe |
| *Pristimantis verrucolatus* | QCAZ46993 | - | MK881485 | MK881377 | Ecuador: Azuay, Cochapamba |
| *Pristimantis versicolor* | KU21981 | EF493389 | EF493389 | EF493431 | Ecuador: Loja, Abra de Zamora (1.7 km E Zamora Chinchipe-Loja border) |
| *Pristimantis versicolor* | MUTPL112 | MT778093 | MT756036 | MT810330 | Ecuador: Loja, Abra de Zamora |
| *Pristimantis versicolor* | MUTPL390 | MT778094 | MT756037 | MT810331 | Ecuador: Loja, Abra de Zamora |
| *Pristimantis versicolor* | MUTPL494 | MT778095 | MT756038 | MT810332 | Ecuador: Loja, Reserva Madrigal del Podocarpus |
| *Pristimantis versicolor* | QCAZ45650 | - | MK881474 | MK881367 | Ecuador: Loja, Parque Nacional Podocarpus, Lagunas del Compadre |
| *Pristimantis vidua* | MUTPL148 | MT778096 | MT764812 | MT810333 | Ecuador: Loja, Bosque Protector Washapamba |
| *Pristimantis vidua* | MUTPL156 | - | MT764813 | MT810334 | Ecuador: Loja, Bosque Protector Washapamba |
| *Pristimantis vidua* | MUTPL296 | MT778097 | MT764814 | MT810335 | Ecuador: Loja, Abra de Zamora |
| *Pristimantis vidua* | MUTPL312 | MT778098 | MT764815 | MT810336 | Ecuador: Loja, Abra de Zamora |
| *Pristimantis vidua* | MUTPL492 | MT778099 | MT764816 | MT810337 | Ecuador: Loja, Parque Nacional Podocarpus, Cajanuma |
| *Pristimantis vidua* | MUTPL495 | MT778100 | MT764817 | MT810338 | Ecuador: Loja, Abra de Zamora |
| *Pristimantis vidua* | MUTPL496 | MT778101 | MT764818 | MT810339 | Ecuador: Loja, Abra de Zamora |
| *Pristimantis vidua* | MUTPL498 | MT778102 | MT764819 | MT810340 | Ecuador: Loja, Abra de Zamora |
| *Pristimantis vidua* | MUTPL499 | MT778103 | MT764820 | MT810341 | Ecuador: Loja, Abra de Zamora |
| *Pristimantis vidua* | MUTPL501 | MT778104 | MT764821 | MT810342 | Ecuador: Loja, Abra de Zamora |
| *Pristimantis vidua* | MUTPL581 | MT778105 | MT764822 | MT810343 | Ecuador: Loja, Parque Nacional Podocarpus, Cajanuma |
| *Pristimantis vidua* | MUTPL586 | MT778106 | MT764823 | MT810344 | Ecuador: Loja, Parque Nacional Podocarpus, Cajanuma |
| *Pristimantis vidua* | MUTPL587 | MT778107 | MT764824 | MT810345 | Ecuador: Loja, Parque Nacional Podocarpus, Cajanuma |
| *Pristimantis* sp. | DHMECN12237 | MK993329 | MK604534 | - | Ecuador: Morona Santiago, Parque Nacional Sangay |
| *Pristimantis* sp. | DHMECN9656 | - | KY967658 | KY967677 | Ecuador: Zamora Chinchipe, Reserva Tapichalaca |
| *Pristimantis* sp. | QCAZ45556 | - | KY967670 | KY967689 | Ecuador: Loja, Parque Nacional Podocarpus, Lagunas del Compadre |
| *Pristimantis* sp. | QCAZ56535 | - | KY967675 | KY967694 | Ecuador: Azuay, Laguna Patococha |
| *Pristimantis* sp. CCS1 | QCAZ32790 | - | MK881423 | MK881328 | Ecuador: Azuay, Bosque Protector Yanuncay-Irquis, Páramo de Quimsacocha |
| *Pristimantis* sp. CCS2 | QCAZ45129 | - | MK881462 | MK881358 | Ecuador: El Oro, Chillacocha |
| *Pristimantis* sp. UCS1 | QCAZ53999 | - | MK881496 | - | Ecuador: Zamora Chinchipe, Yacuambi, Romerillos |
| *Pristimantis* sp. UCS2 | QCAZ45029 | - | MK881461 | - | Ecuador: Morona Santiago, Parque Nacional Sangay, Etén, Rio Culebrillas |
| *Pristimantis* sp. UCS3 | QCAZ26642 | - | MK881409 | - | Ecuador: Azuay, San Antonio de Chaucha |
